# Flexible, Functional, and Familiar: Characteristics of SARS-CoV-2 Spike Protein Evolution

**DOI:** 10.3389/fmicb.2020.02112

**Published:** 2020-09-17

**Authors:** Dianita S. Saputri, Songling Li, Floris J. van Eerden, John Rozewicki, Zichang Xu, Hendra S. Ismanto, Ana Davila, Shunsuke Teraguchi, Kazutaka Katoh, Daron M. Standley

**Affiliations:** ^1^Department of Genome Informatics, Research Institute for Microbial Diseases, Osaka University, Suita, Japan; ^2^Immunology Frontier Research Center, Osaka University, Suita, Japan

**Keywords:** flexibility, host like, molecular evolution, phylogenetics, SARS-CoV-2, spike protein, structural modeling, structure alignment

## Abstract

The SARS-CoV-2 S protein is a major point of interaction between the virus and the human immune system. As a consequence, the S protein is not a static target but undergoes rapid molecular evolution. In order to more fully understand the selection pressure during evolution, we examined residue positions in the S protein that vary greatly across closely related viruses but are conserved in the subset of viruses that infect humans. These “evolutionarily important” residues were not distributed evenly across the S protein but were concentrated in two domains: the N-terminal domain and the receptor-binding domain, both of which play a role in host cell binding in a number of related viruses. In addition to being localized in these two domains, evolutionary importance correlated with structural flexibility and inversely correlated with distance from known or predicted host receptor-binding residues. Finally, we observed a bias in the composition of the amino acids that make up such residues toward more human-like, rather than virus-like, sequence motifs.

## Introduction

**FIGURE 1:**
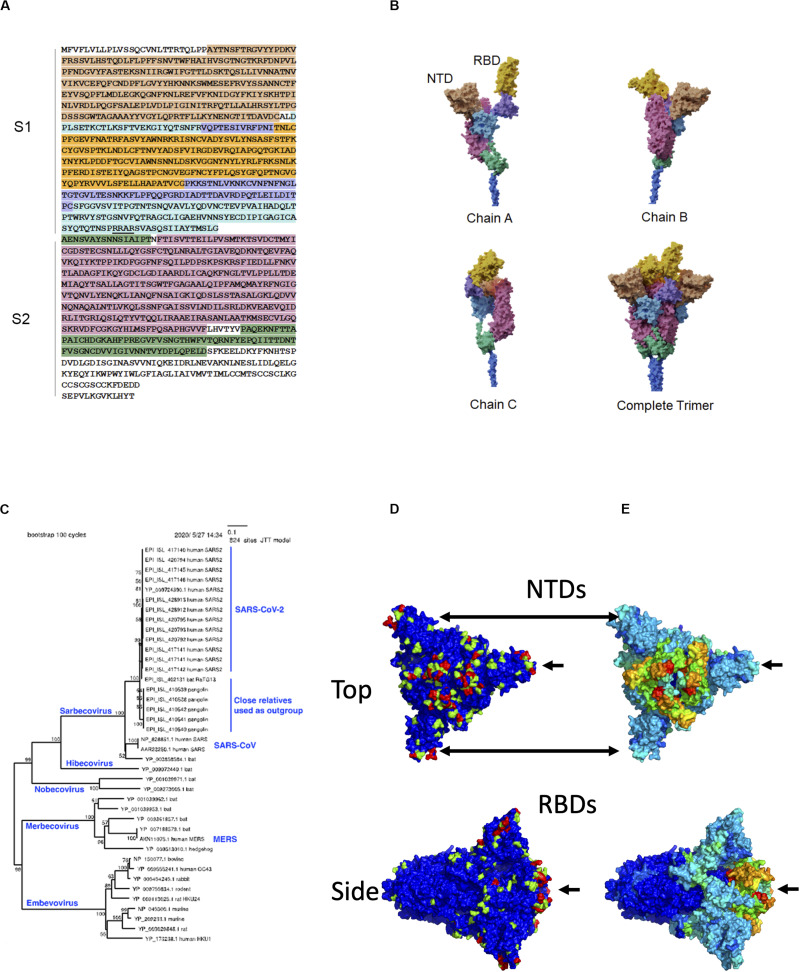
Sequence, structure, and evolution of SARS-CoV-2 spike protein. **(A)** The sequence of the ectodomain is obtained from Uniprot with accession number P0DTC2 and is shaded according to six structural domains that correspond to those of **(B)**. The trimeric ectodomain can be divided into S1 and S2 subunits. In this figure, S1 is defined as residues 27–700 and S2 as 701–1,146. The S1 subunit can be further divided into the N-terminal domain (NTD, residues 27–291) and C-terminal domain (CTD, residues 294–700). The S1 CTD can further be divided into smaller structural domains, including the receptor-binding domain (RBD), in magenta, which is composed of residues 333–526 in this figure. The furin “RRAR” cleavage site at the end of S1 is underlined. **(B)** The three chains are interwoven to form the complete trimer. Here, the overall model was based on PDB entry 6vsb with NTD domains replaced by those of entry 5×4s. **(C)** Phylogenetic tree of closely related spike proteins from viruses that infect humans along with a representative outgroup. **(D)** Molecular surface of spike with evolutionary importance represented as a heatmap at three levels: low (blue), medium (green), and high (red). **(E)** Molecular surface of spike with evolutionary rate represented as a heatmap on a scale from lowest (blue) to highest (red).

Over 200 viruses are known to infect humans (Woolhouse et al., 2012). Among recent human virus outbreaks, three (SARS-CoV-1, MERS-CoV, and SARS-CoV-2) have arisen from beta coronaviruses. The close interaction between pathogen and host can be a driving force for molecular evolution. This is nowhere more apparent than on the surfaces of the viruses themselves. The characteristic crown-shaped spikes, for which coronaviruses are named, enable binding to and entering host cells, and also provide camouflage from the host immune system. The ectodomain – the most outer part of the spike (S) protein – consists of two functional subunits, the receptor-binding subunit (S1) and the membrane fusion subunit (S2) (Figure 1A). The S1 subunits are highly variable across genera, while the S2 subunits are much more conserved. These differences reflect their distinct functions: Whereas the S1 regions engage with receptors on the surfaces of host cells, the primary function of S2 is to mediate fusion with host cell membranes. The S1 subunit is located within the N-terminus of the S protein and can be further divided into an N-terminal domain (NTD) and a C-terminal domain, which, in itself, can be divided into a receptor-binding domain (RBD) located at the apex of the protein when viewed from the side and two additional domains connecting it to the NTD (Wang et al., 2020). In SARS-CoV-2, the RBD contains a receptor-binding motif (437–508) that contains host receptor-binding residues. The structural domains of the S protein wind around each other such that the three RBDs and NTDs constitute a nearly continuous surface at the apex of the trimeric protein (Figure 1B).

The targets of the S1 NTD and RBD can differ greatly among beta coronaviruses. For example, the NTD can recognize sugar derivatives in human coronavirus (HCoV)-HKU1 and HCoV-OC43, which facilitate attachment to host cells; in mouse hepatitis coronavirus (MHV), the NTD binds to the host protein carcinoembryonic antigen-related cell adhesion molecule 1 (CEACAM1). Meanwhile, the RBD binds hACE2 in SARS-CoV-1 and SARS-CoV-2, but binds aminopeptidase N (APN) and dipeptidyl peptidase 4 (DPP4) in HCoV-229E and MERS-CoV, respectively (Wang et al., 2020). This large variability in binding partners suggests that NTD and RBD are sites of intense evolutionary pressure.

In order to better understand this evolutionary pressure, we estimated the evolutionary importance of residue positions in SARS-CoV-2 by comparing the amino acid diversity of each position to that of equivalent positions in closely related viruses that infect non-human hosts. We found that evolutionary importance was high in the NTD and RBD. Moreover, within these domains, residues with high evolutionary importance could be characterized by three features: they are more *flexible* (when simulated by molecular dynamics) than surrounding residues, they occur in or around known *functionally important* host – protein binding sites, and their sequences are much more self-like or *familiar* to the host immune system than other residues.

### Estimating Evolutionary Importance

It is possible to infer evolutionarily important residues in the S protein by observing sites that are conserved within a given branch of the phylogenetic tree but vary among different branches. To construct a phylogenetic tree, 20 SARS-CoV-2 S protein sequences, 6 close outgroups that infect bat and pangolin, and several sequences from other lineages of beta coronavirus (SARS-CoV, MERS-CoV, and HCoV-HKU1) were collected. Amino acid sequences were aligned by MAFFT (Katoh and Standley, 2013), and a neighbor-joining (Saitou and Nei, 1987) tree was estimated to roughly visualize the phylogenetic relationship (Figure 1C). We subsequently estimated the sequence diversity at each position using 9,827 SARS-CoV-2 sequences (https://www.gisaid.org; after filtering out those with many ambiguous bases and/or fragmentary sequences). These sequences were compared only with the close outgroups that infect bat and pangolin. The diversity for the combination of human + outgroup and for the human group alone was compared. We defined “evolutionary importance” as the difference:

diversity⁢(human+outgroup)-diversity⁢(human)

assuming that this difference reflects the change in evolutionary pressure when this virus is transmitted to humans. This resulted in three levels of importance: low (0), medium (1), and high (2) as indicated in the heatmap projected onto the molecular surface of the S protein. While low- and medium-importance positions were distributed widely across the S protein surface, most of the positions with high importance were confined to two domains: the NTD and the RBD (Figure 1D).

For comparison, local evolutionary rate in the human-infecting lineage was estimated by (100 AA) sliding window analysis. The evolutionary rate in this lineage is proportional to the evolutionary distance between the present-day sequences infecting humans and the common ancestor of human-infecting and bat-infecting lineages. The average distance, *D*, between these two points was estimated, for each window, by the relative rate test (Sarich, 1969):

$$D=\frac{1}{2}\,\left\{d\,\left(h,p\right)+d\,\left(h,b\right)-d\,\left(b,p\right)\right\}$$

where, *h, p, b* denote human, pangolin, and bat, respectively; pairwise distance was computed using the Poisson correction, d(,) = −ln(1−x/y), where *x* is the number of differences between sequences, and *y* is the number of sites. A high evolutionary rate in this lineage was clearly observed near hACE2-binding sites (Figure 1E). This observation is consistent with the site-specific diversity observed in the evolutionary importance; such sites have apparently changed radically upon transfer to humans and have been highly conserved thereafter. However, regarding the NTD region, the evolutionary rate was not estimated to be as high as the RBD in the human lineage. This local evolutionary rate analysis has three limitations: (1) it uses average rates of multiple adjacent residues, (2) it does not consider conservation within the human-infecting lineage, and (3) it cannot distinguish changes in a specific lineage from background changes in the same region. By defining evolutionary importance as we have above, we clearly observe sites that are specifically conserved in the lineage infecting humans.

### Evolutionary Importance of Flexible Regions

**FIGURE 2:**
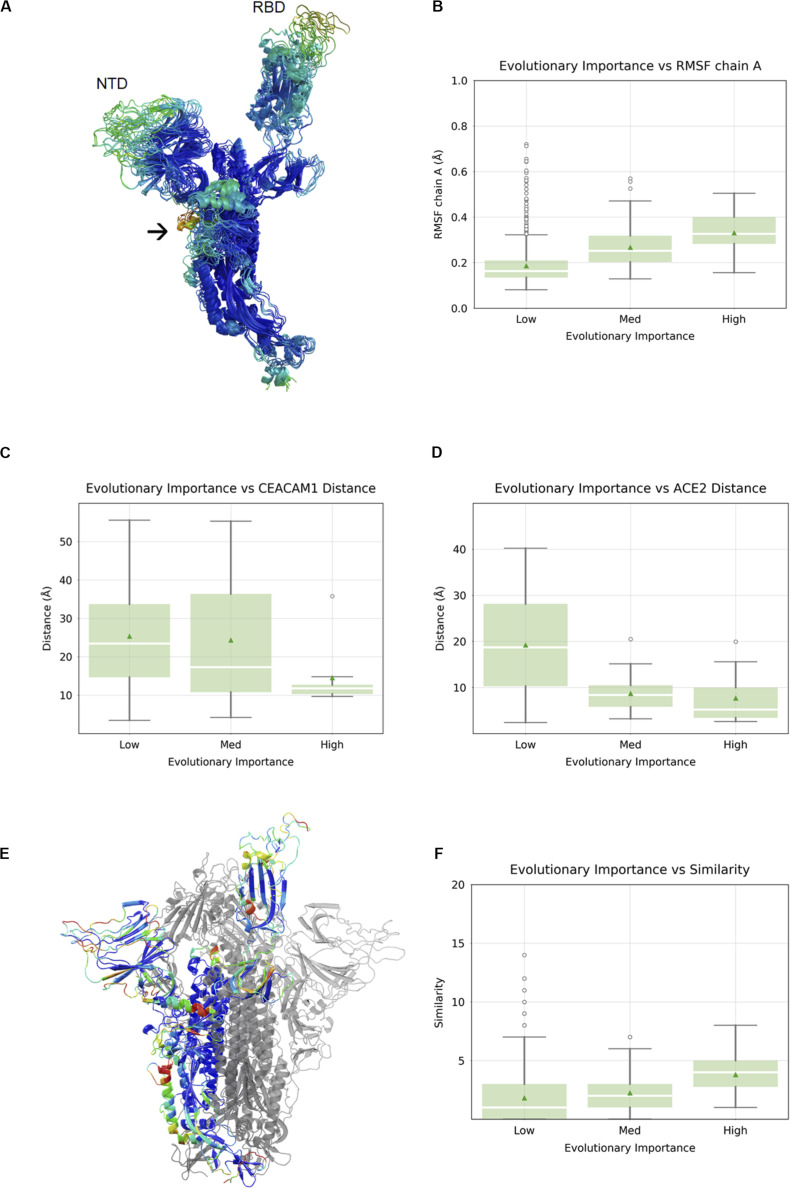
Correlation between evolutionary importance and other properties. **(A)** The ectodomain of the spike protein colored according to the root-mean square fluctuation (RMSF), with the scale going from blue (low RMSF), via green and yellow to red (high RMSF). The NTD and RBD are labeled, and an arrow indicates the location of the fusion peptide. **(B)** Boxplot of RMSF as a function of evolutionary importance indicating that evolutionary importance is higher in flexible regions. **(C)** Boxplot of distance from carcinoembryonic antigen-related cell adhesion molecule 1 (CEACAM1) interface, as inferred from superposition on MHV, for residues in the three groups of evolutionary importance. **(D)** Boxplot of distance from ACE2-binding site for residues in the three groups of evolutionary importance. **(E)** Similarity is represented as a heatmap (from low to high: blue, cyan, green, yellow, orange, red) on a single chain of the spike protein with the other two chains shown in gray. **(F)** Boxplots showing similarity to human proteins highest for evolutionary important residues.

It has been established that in SARS-CoV-1 and SARS-CoV-2, the RBD undergoes a large conformational change from the “closed” state to the “open” state upon engagement with hACE2 (Walls et al., 2020; Wrapp et al., 2020). In order to visualize flexible regions in the SARS-CoV-2 S protein, we carried out molecular dynamic simulations of the S protein in the open conformation followed by the root-mean square fluctuation (RMSF) analysis (Figure 2A). Not surprisingly, the most flexible parts of the protein were in loop regions. We observed that the beta sheet cores of both the S1 NTD and RBD domains were stable, as was most (but not all) of the S2 subunits. There were two exceptions with a higher RMSF: residue alanine 684 is part of the furin cleavage site (RRAR), which has been shown to be essential for infection of human lung cells (Hoffmann et al., 2020) and residues 830–840, which constitute a fusion peptide. Overall, we observed a nearly linear correlation between evolutionary importance and mean RMSF of these regions (Spearman correlation 0.30, *p* < 2.2 × 10^–16^) (Figure 2B). It is possible that flexibility in the NTD and RBD loops provide an induced-fit binding mechanism, wherein loop regions rearrange in order to properly bind to their host receptors. To explore this idea further, we analyze the relationship between evolutionary importance and distance from host receptor-binding sites below.

### Evolutionary Importance and Proximity to Functional Binding Sites

The SARS-CoV-2 RBD mediates host cell entry by binding to hACE2. While the target of the SARS-CoV-2 NTD is still unknown, the high evolutionary importance in the NTD suggests a potential binding partner. Even without knowing the target of the NTD, we can assume that the location of the binding site is roughly conserved, and the distance of each residue in the NTD from this location using the NTDs of other viruses as proxies was measured. We can, of course, perform a similar and more precise analysis in the RBD using the known RBD–ACE2 complex crystal structure (Lan et al., 2020). When we compared the evolutionary importance in SARS-CoV-2 S1-NTD with the distance to the MHV NTD–CEACAM1 interface (Peng et al., 2011), we observed a negative correlation with distance (Spearman correlation -0.19, *p* = 1.8 × 10^–3^) (Figure 2C). The fact that evolutionary importance is higher in residues located near the equivalent site suggests that SARS-CoV-2 S1-NTD may have retained host binding and that the location of the binding site is roughly conserved. We compared the evolutionary importance with the distance to the hACE2-binding site (Yan et al., 2020) and observed that the evolutionary importance was higher in residues located near the ACE2 interface, consistent with its functional importance (Spearman correlation -0.38, *p* < 8.8 × 10^–8^) (Figure 2D). Taken together, we can say that evolutionary important residues occur often in flexible loops in or near known or putative virus – host binding interfaces.

### Evolutionary Importance of Host-Like Sequences

Since the outer parts of the virus are most exposed to the host immune system, we aimed to look for their similarity with human cell surface proteins, as such similarity may indicate immune evasion. We carried out local alignment of all five-residue sequence fragments with a representative set of 507 human cell surface proteins as annotated by the Cell Surface Protein Atlas (Bausch-Fluck et al., 2015). The local sequence similarity was computed for each SARS-CoV-2 residue using rigorous matching criteria for each fragment. This analysis revealed several hotspots of similarity, including the NTD and RBD (Figure 2E). We quantified the relationship between similarity to human cell surface proteins and evolutionary importance and found that the similarity was highest for residues with the greatest importance (Spearman correlation 0.13, *p* < 7.7 × 10^–6^) (Figure 2F).

## Discussion

We estimated evolutionary importance based on generally diverse residue positions that are conserved within the SARS-CoV-2. We observed that such residues were primarily restricted to two domains, the NTD and RBD, both of which have host receptor-binding functions in a number of closely related viruses. Interestingly, these “important” residues were more flexible than less important residues, suggesting that the flexibility is a characteristic of rapid molecular evolution. Moreover, the residues tended to cluster near or within known or predicted host receptor-binding sites. This is not surprising, since the Evolutionary Trace method, on which our simple definition of evolutionary importance was based, has widely been used for predicting protein – protein interactions (Wodak and Mendez, 2004).

The fact that the NTD includes many evolutionary important residues strongly hints at a role in host receptor binding. Moreover, the correlation of evolutionary importance with distance from the known CEACAM1-binding site implies that the location of the binding site might be conserved. A recent report that anti-NTD antibodies can be neutralizing (Chi et al., 2020) supports this notion. We observed that evolutionary important residues appeared to be biased toward “human-like” sequence motifs more than other residues suggesting that they may have more potential to evade the immune system through mimicking the host protein. Although the sequence data on SARS-CoV-2 is still limited, the patterns may provide clues about the identity of targeted human cell surface receptors.

## Data Availability Statement

All datasets presented in this study are included in the article/Supplementary Material.

## Author Contributions

DS carried out structural analysis of domains in related viruses. SL constructed a full-length model of SARS-CoV-2 S protein. FE performed molecular dynamics simulations of S protein. JR developed software for structural alignment. ZX carried out S protein docking. HI performed sequence analysis of SARS-CoV-2 S protein. AD carried out immunogenic (epitope) prediction on S protein. ST performed statistics calculations. KK did phylogenetic analysis. DS conceived of the project and wrote the manuscript. All authors contributed to the article and approved the submitted version.

## Conflict of Interest

The authors declare that the research was conducted in the absence of any commercial or financial relationships that could be construed as a potential conflict of interest.
