# Supplementary material for: Flexible, Functional, and Familiar: Characteristics of SARS-CoV-2 Spike Protein Evolution
Source: Front Microbiol. 2020 Sep 17;11:2112. doi: 10.3389/fmicb.2020.02112 (PMC7527407; doi:10.3389/fmicb.2020.02112)
Supplement: Supplementary file 1 [file Table_1.DOCX]

***Supplementary Material***

1. **Supplementary Data**

Tabel 1 Evolutionary important residues

| Res  num | Res  name | Raw  rate | Human  only  rate | With close out groups rate | Evolutionary importance | ACE2 distance (Å) | CEACAM1 distance (Å) | Human protein hits | RMSF chain A (Å) | RMSF chain B (Å) | RMSF chain C (Å) |
| --- | --- | --- | --- | --- | --- | --- | --- | --- | --- | --- | --- |
| 1 | M | 1 | 1 | 1 | 0 |  |  |  |  |  |  |
| 2 | F | 5 | 1 | 1 | 0 |  |  |  |  |  |  |
| 3 | V | 5 | 1 | 1 | 0 |  |  |  |  |  |  |
| 4 | F | 5 | 1 | 1 | 0 |  |  |  |  |  |  |
| 5 | L | 5 | 2 | 2 | 0 |  |  |  |  |  |  |
| 6 | V | 4 | 1 | 2 | 1 |  |  |  |  |  |  |
| 7 | L | 9 | 1 | 2 | 1 |  |  |  |  |  |  |
| 8 | L | 5 | 3 | 3 | 0 |  |  |  |  |  |  |
| 9 | P | 8 | 3 | 3 | 0 |  |  |  |  |  |  |
| 10 | L | 5 | 1 | 1 | 0 |  |  |  |  |  |  |
| 11 | V | 6 | 1 | 1 | 0 |  |  |  |  |  |  |
| 12 | S | 7 | 2 | 2 | 0 |  |  |  |  |  |  |
| 13 | S | 4 | 1 | 1 | 0 |  |  |  |  |  |  |
| 14 | Q | 7 | 3 | 3 | 0 |  |  |  |  |  |  |
| 15 | C | 3 | 1 | 1 | 0 |  |  |  |  |  |  |
| 16 | V | 6 | 1 | 1 | 0 |  |  |  |  |  |  |
| 17 | N | 8 | 2 | 2 | 0 |  |  |  |  |  |  |
| 18 | L | 4 | 2 | 2 | 0 |  |  |  |  |  |  |
| 19 | T | 5 | 1 | 1 | 0 |  |  |  |  |  |  |
| 20 | T | 8 | 1 | 1 | 0 |  |  |  |  |  |  |
| 21 | R | 5 | 3 | 3 | 0 |  |  |  |  |  |  |
| 22 | T | 5 | 2 | 2 | 0 |  |  |  |  |  |  |
| 23 | Q | 6 | 2 | 3 | 1 |  |  |  |  |  |  |
| 24 | L | 6 | 1 | 2 | 1 |  |  |  |  |  |  |
| 25 | P | 9 | 2 | 3 | 1 |  |  |  |  |  |  |
| 26 | P | 8 | 2 | 2 | 0 |  |  |  |  |  |  |
| 27 | A | 9 | 3 | 4 | 1 |  | 21.64 | 4 | 0.2178 | 0.3666 | 0.275 |
| 28 | Y | 6 | 2 | 2 | 0 |  | 22.86 | 2 | 0.1803 | 0.3167 | 0.2675 |
| 29 | T | 7 | 2 | 2 | 0 |  | 26.39 | 1 | 0.1704 | 0.273 | 0.2637 |
| 30 | N | 5 | 2 | 2 | 0 |  | 30.1 | 0 | 0.1624 | 0.2435 | 0.2569 |
| 31 | S | 1 | 1 | 1 | 0 |  | 99.999 | 0 | 0.1488 | 0.221 | 0.2424 |
| 32 | F | 4 | 3 | 4 | 1 |  | 34.92 | 0 | 0.1562 | 0.2145 | 0.2412 |
| 33 | T | 8 | 1 | 1 | 0 |  | 37.92 | 0 | 0.152 | 0.1885 | 0.2217 |
| 34 | R | 2 | 1 | 1 | 0 |  | 34.75 | 0 | 0.1427 | 0.1742 | 0.2032 |
| 35 | G | 1 | 1 | 1 | 0 |  | 99.999 | 0 | 0.1483 | 0.1785 | 0.1851 |
| 36 | V | 2 | 1 | 1 | 0 |  | 38.47 | 0 | 0.1426 | 0.1438 | 0.1918 |
| 37 | Y | 1 | 1 | 1 | 0 |  | 99.999 | 0 | 0.1302 | 0.1247 | 0.1735 |
| 38 | Y | 1 | 1 | 1 | 0 |  | 41.49 | 0 | 0.1398 | 0.1185 | 0.1752 |
| 39 | P | 3 | 1 | 1 | 0 |  | 42.13 | 0 | 0.1371 | 0.1208 | 0.2042 |
| 40 | D | 1 | 1 | 1 | 0 |  | 43.59 | 0 | 0.1247 | 0.1171 | 0.2088 |
| 41 | K | 4 | 1 | 1 | 0 |  | 40.21 | 0 | 0.125 | 0.1173 | 0.2357 |
| 42 | V | 2 | 2 | 2 | 0 |  | 47.45 | 0 | 0.148 | 0.11 | 0.1888 |
| 43 | F | 2 | 1 | 1 | 0 |  | 50.18 | 0 | 0.1908 | 0.1077 | 0.1802 |
| 44 | R | 1 | 1 | 1 | 0 |  | 48.52 | 0 | 0.1865 | 0.1164 | 0.1678 |
| 45 | S | 1 | 1 | 1 | 0 |  | 54.05 | 0 | 0.2211 | 0.1294 | 0.1585 |
| 46 | S | 3 | 1 | 1 | 0 |  | 55.58 | 0 | 0.2705 | 0.1381 | 0.1638 |
| 47 | V | 5 | 1 | 2 | 1 |  | 53.53 | 0 | 0.2413 | 0.1414 | 0.1592 |
| 48 | L | 4 | 2 | 2 | 0 |  | 53.07 | 0 | 0.1863 | 0.1455 | 0.1587 |
| 49 | H | 3 | 2 | 2 | 0 |  | 48.66 | 0 | 0.1679 | 0.1428 | 0.1722 |
| 50 | S | 2 | 2 | 2 | 0 |  | 48.96 | 0 | 0.1603 | 0.1567 | 0.1688 |
| 51 | T | 3 | 1 | 1 | 0 |  | 45.41 | 0 | 0.1547 | 0.1825 | 0.2034 |
| 52 | Q | 3 | 1 | 1 | 0 |  | 43.03 | 0 | 0.1525 | 0.1862 | 0.2238 |
| 53 | D | 2 | 1 | 1 | 0 |  | 39.52 | 0 | 0.1378 | 0.1819 | 0.2064 |
| 54 | L | 5 | 3 | 3 | 0 |  | 35.02 | 0 | 0.1307 | 0.1812 | 0.1915 |
| 55 | F | 1 | 2 | 2 | 0 |  | 99.999 | 1 | 0.1295 | 0.1642 | 0.192 |
| 56 | L | 1 | 1 | 1 | 0 |  | 32.57 | 3 | 0.1311 | 0.178 | 0.2042 |
| 57 | P | 2 | 2 | 2 | 0 |  | 35.57 | 3 | 0.1438 | 0.1937 | 0.2402 |
| 58 | F | 1 | 1 | 1 | 0 |  | 37.19 | 6 | 0.1505 | 0.202 | 0.2402 |
| 59 | F | 6 | 1 | 1 | 0 |  | 34.29 | 6 | 0.153 | 0.2239 | 0.262 |
| 60 | S | 2 | 1 | 1 | 0 |  | 31.56 | 5 | 0.1475 | 0.2376 | 0.2715 |
| 61 | N | 2 | 1 | 1 | 0 |  | 28.71 | 3 | 0.1575 | 0.2599 | 0.2601 |
| 62 | V | 2 | 2 | 2 | 0 |  | 99.999 | 3 | 0.1464 | 0.2583 | 0.2425 |
| 63 | T | 3 | 1 | 1 | 0 |  | 21.14 | 0 | 0.1502 | 0.2922 | 0.2384 |
| 64 | W | 4 | 2 | 2 | 0 |  | 20.29 | 0 | 0.1611 | 0.3036 | 0.2432 |
| 65 | F | 3 | 1 | 1 | 0 |  | 99.999 | 0 | 0.1742 | 0.3234 | 0.2389 |
| 66 | H | 7 | 1 | 2 | 1 |  | 16.82 | 0 | 0.2186 | 0.3451 | 0.2739 |
| 67 | A | 3 | 1 | 2 | 1 |  | 17.32 | 0 | 0.273 | 0.3229 | 0.2872 |
| 68 | I | 6 | 1 | 1 | 0 |  | 14.58 | 1 | 0.335 | 0.3605 | 0.2952 |
| 69 | H | 6 | 3 | 5 | 2 |  | 14.78 | 2 | 0.3328 | 0.3904 | 0.342 |
| 70 | V | 10 | 2 | 4 | 2 |  | 11.89 | 4 | 0.3377 | 0.4357 | 0.3339 |
| 71 | S | 9 | 3 | 5 | 2 |  | 12.01 | 5 | 0.3764 | 0.5621 | 0.4705 |
| 72 | G | 3 | 2 | 3 | 1 |  | 10.79 | 7 | 0.3997 | 0.6537 | 0.4828 |
| 73 | T | 6 | 2 | 4 | 2 |  | 11.72 | 6 | 0.4433 | 0.6938 | 0.3767 |
| 74 | N | 6 | 2 | 4 | 2 |  | 10.41 | 6 | 0.4483 | 0.6703 | 0.3284 |
| 75 | G | 7 | 3 | 3 | 0 |  | 9.02 | 5 | 0.4742 | 0.5112 | 0.3122 |
| 76 | T | 10 | 2 | 4 | 2 |  | 9.68 | 5 | 0.4133 | 0.4582 | 0.2938 |
| 77 | K | 5 | 2 | 2 | 0 |  | 11.71 | 2 | 0.4035 | 0.3926 | 0.3006 |
| 78 | R | 4 | 2 | 2 | 0 |  | 7.66 | 2 | 0.3532 | 0.3965 | 0.3032 |
| 79 | F | 5 | 1 | 1 | 0 |  | 13.2 | 2 | 0.2993 | 0.3835 | 0.2955 |
| 80 | D | 3 | 2 | 2 | 0 |  | 10.48 | 5 | 0.2655 | 0.373 | 0.2819 |
| 81 | N | 2 | 1 | 1 | 0 |  | 13.31 | 5 | 0.2183 | 0.4055 | 0.2403 |
| 82 | P | 3 | 1 | 1 | 0 |  | 14.01 | 5 | 0.2106 | 0.4073 | 0.2427 |
| 83 | V | 4 | 2 | 2 | 0 |  | 13.79 | 8 | 0.1833 | 0.3635 | 0.216 |
| 84 | L | 3 | 1 | 1 | 0 |  | 17.73 | 7 | 0.1568 | 0.358 | 0.2141 |
| 85 | P | 4 | 1 | 1 | 0 |  | 20.21 | 4 | 0.1524 | 0.3065 | 0.2248 |
| 86 | F | 1 | 1 | 1 | 0 |  | 99.999 | 3 | 0.1353 | 0.2524 | 0.1998 |
| 87 | N | 6 | 1 | 1 | 0 |  | 25.32 | 3 | 0.1764 | 0.2804 | 0.2259 |
| 88 | D | 2 | 1 | 1 | 0 |  | 29.38 | 0 | 0.1658 | 0.236 | 0.213 |
| 89 | G | 1 | 1 | 1 | 0 |  | 99.999 | 0 | 0.133 | 0.2012 | 0.1828 |
| 90 | V | 2 | 2 | 2 | 0 |  | 99.999 | 0 | 0.1274 | 0.2024 | 0.1789 |
| 91 | Y | 1 | 1 | 1 | 0 |  | 99.999 | 0 | 0.1304 | 0.1874 | 0.1798 |
| 92 | F | 2 | 1 | 1 | 0 |  | 99.999 | 0 | 0.1293 | 0.2043 | 0.1781 |
| 93 | A | 3 | 1 | 1 | 0 |  | 99.999 | 0 | 0.1428 | 0.2099 | 0.1889 |
| 94 | S | 4 | 2 | 2 | 0 |  | 99.999 | 0 | 0.1554 | 0.2494 | 0.1951 |
| 95 | T | 3 | 2 | 2 | 0 |  | 25.39 | 0 | 0.1737 | 0.2509 | 0.2166 |
| 96 | E | 2 | 2 | 2 | 0 |  | 22.85 | 0 | 0.1836 | 0.2499 | 0.2169 |
| 97 | K | 3 | 1 | 1 | 0 |  | 23.15 | 0 | 0.212 | 0.2741 | 0.2335 |
| 98 | S | 1 | 2 | 2 | 0 |  | 20.59 | 0 | 0.2401 | 0.2857 | 0.225 |
| 99 | N | 1 | 1 | 1 | 0 |  | 20.39 | 0 | 0.2106 | 0.2769 | 0.21 |
| 100 | I | 2 | 1 | 1 | 0 |  | 17.98 | 0 | 0.1934 | 0.2874 | 0.2099 |
| 101 | I | 3 | 1 | 1 | 0 |  | 20.97 | 0 | 0.171 | 0.2638 | 0.1906 |
| 102 | R | 1 | 2 | 2 | 0 |  | 17.98 | 0 | 0.1738 | 0.2589 | 0.1839 |
| 103 | G | 1 | 1 | 1 | 0 |  | 99.999 | 0 | 0.1528 | 0.2322 | 0.1705 |
| 104 | W | 1 | 2 | 2 | 0 |  | 21.19 | 0 | 0.1344 | 0.2267 | 0.1657 |
| 105 | I | 2 | 1 | 1 | 0 |  | 17.79 | 1 | 0.1439 | 0.2397 | 0.1673 |
| 106 | F | 1 | 1 | 1 | 0 |  | 99.999 | 3 | 0.1375 | 0.2383 | 0.1699 |
| 107 | G | 1 | 1 | 1 | 0 |  | 99.999 | 4 | 0.1462 | 0.2536 | 0.18 |
| 108 | T | 2 | 1 | 1 | 0 |  | 18.49 | 5 | 0.1529 | 0.2705 | 0.1949 |
| 109 | T | 2 | 1 | 1 | 0 |  | 15.58 | 7 | 0.176 | 0.294 | 0.2133 |
| 110 | L | 3 | 2 | 2 | 0 |  | 14.7 | 7 | 0.1775 | 0.2881 | 0.21 |
| 111 | D | 2 | 2 | 2 | 0 |  | 13.78 | 6 | 0.2045 | 0.3245 | 0.224 |
| 112 | S | 3 | 1 | 2 | 1 |  | 11.85 | 5 | 0.2451 | 0.3657 | 0.2201 |
| 113 | K | 4 | 2 | 3 | 1 |  | 13.74 | 4 | 0.2471 | 0.3829 | 0.2341 |
| 114 | T | 2 | 1 | 1 | 0 |  | 17.91 | 2 | 0.2059 | 0.325 | 0.1984 |
| 115 | Q | 1 | 2 | 2 | 0 |  | 20.15 | 1 | 0.1876 | 0.2889 | 0.1797 |
| 116 | S | 2 | 1 | 1 | 0 |  | 18.51 | 1 | 0.1722 | 0.2664 | 0.1729 |
| 117 | L | 3 | 1 | 1 | 0 |  | 99.999 | 1 | 0.1529 | 0.2387 | 0.1611 |
| 118 | L | 3 | 1 | 1 | 0 |  | 17.46 | 0 | 0.1546 | 0.2391 | 0.1617 |
| 119 | I | 2 | 2 | 2 | 0 |  | 22.1 | 0 | 0.1415 | 0.2197 | 0.1596 |
| 120 | V | 5 | 2 | 2 | 0 |  | 20.51 | 0 | 0.1589 | 0.2389 | 0.168 |
| 121 | N | 1 | 1 | 1 | 0 |  | 21.53 | 0 | 0.1795 | 0.2457 | 0.1749 |
| 122 | N | 1 | 1 | 1 | 0 |  | 19.61 | 2 | 0.2069 | 0.2688 | 0.2247 |
| 123 | A | 3 | 1 | 1 | 0 |  | 99.999 | 2 | 0.2255 | 0.2899 | 0.2231 |
| 124 | T | 2 | 1 | 1 | 0 |  | 22.18 | 4 | 0.2075 | 0.2698 | 0.2121 |
| 125 | N | 3 | 1 | 1 | 0 |  | 24.56 | 4 | 0.1811 | 0.2404 | 0.1893 |
| 126 | V | 3 | 1 | 1 | 0 |  | 25.27 | 5 | 0.1652 | 0.2202 | 0.1781 |
| 127 | V | 4 | 1 | 1 | 0 |  | 21.1 | 3 | 0.1625 | 0.2286 | 0.1696 |
| 128 | I | 2 | 2 | 2 | 0 |  | 99.999 | 3 | 0.1533 | 0.218 | 0.1623 |
| 129 | K | 5 | 1 | 1 | 0 |  | 17.1 | 1 | 0.1745 | 0.2382 | 0.1618 |
| 130 | V | 2 | 1 | 1 | 0 |  | 99.999 | 1 | 0.18 | 0.247 | 0.1671 |
| 131 | C | 1 | 1 | 1 | 0 |  | 99.999 | 0 | 0.1879 | 0.2785 | 0.1756 |
| 132 | E | 4 | 1 | 1 | 0 |  | 14.02 | 0 | 0.204 | 0.318 | 0.1897 |
| 133 | F | 1 | 1 | 1 | 0 |  | 12.71 | 0 | 0.2086 | 0.3219 | 0.198 |
| 134 | Q | 6 | 1 | 1 | 0 |  | 8.18 | 0 | 0.2495 | 0.3593 | 0.2252 |
| 135 | F | 3 | 1 | 1 | 0 |  | 10.83 | 0 | 0.2783 | 0.3507 | 0.2464 |
| 136 | C | 1 | 1 | 1 | 0 |  | 9.68 | 0 | 0.4026 | 0.4144 | 0.2882 |
| 137 | N | 7 | 1 | 2 | 1 |  | 7.75 | 0 | 0.4706 | 0.3987 | 0.2839 |
| 138 | D | 4 | 3 | 3 | 0 |  | 8.42 | 1 | 0.399 | 0.4221 | 0.2679 |
| 139 | P | 1 | 2 | 2 | 0 |  | 99.999 | 1 | 0.3646 | 0.4305 | 0.2337 |
| 140 | F | 5 | 3 | 3 | 0 |  | 99.999 | 1 | 0.2651 | 0.38 | 0.2238 |
| 141 | L | 4 | 2 | 2 | 0 |  | 9.49 | 2 | 0.2622 | 0.3791 | 0.2557 |
| 142 | G | 6 | 4 | 4 | 0 |  | 99.999 | 2 | 0.2391 | 0.3779 | 0.2816 |
| 143 | V | 3 | 3 | 3 | 0 |  | 99.999 | 1 | 0.2526 | 0.3708 | 0.2949 |
| 144 | Y | 8 | 2 | 2 | 0 |  | 10.02 | 1 | 0.2588 | 0.3696 | 0.2989 |
| 145 | Y | 10 | 3 | 3 | 0 |  | 11.75 | 2 | 0.2974 | 0.4008 | 0.3288 |
| 146 | H | 6 | 3 | 3 | 0 |  | 10.9 | 1 | 0.3035 | 0.3939 | 0.3373 |
| 147 | K | 8 | 1 | 2 | 1 |  | 8.09 | 2 | 0.3153 | 0.3875 | 0.3488 |
| 148 | N | 5 | 3 | 3 | 0 |  | 9.69 | 2 | 0.3309 | 0.3663 | 0.3504 |
| 149 | N | 9 | 2 | 2 | 0 |  | 11.13 | 2 | 0.3026 | 0.3523 | 0.3501 |
| 150 | K | 7 | 1 | 1 | 0 |  | 13.64 | 1 | 0.3242 | 0.3663 | 0.3436 |
| 151 | S | 3 | 2 | 3 | 1 |  | 15.27 | 1 | 0.3395 | 0.3707 | 0.3575 |
| 152 | W | 6 | 2 | 2 | 0 |  | 14.5 | 0 | 0.3027 | 0.369 | 0.3241 |
| 153 | M | 4 | 3 | 3 | 0 |  | 13.29 | 1 | 0.2873 | 0.405 | 0.2978 |
| 154 | E | 3 | 1 | 1 | 0 |  | 13.13 | 4 | 0.2658 | 0.3816 | 0.275 |
| 155 | S | 4 | 1 | 2 | 1 |  | 8.57 | 4 | 0.333 | 0.3838 | 0.301 |
| 156 | E | 2 | 2 | 2 | 0 |  | 6.61 | 6 | 0.297 | 0.3813 | 0.2722 |
| 157 | F | 2 | 3 | 3 | 0 |  | 12.49 | 6 | 0.2364 | 0.3657 | 0.2377 |
| 158 | R | 3 | 1 | 1 | 0 |  | 6.34 | 5 | 0.3049 | 0.3647 | 0.2409 |
| 159 | V | 3 | 1 | 1 | 0 |  | 12.11 | 3 | 0.2941 | 0.3588 | 0.2105 |
| 160 | Y | 2 | 1 | 1 | 0 |  | 9.24 | 3 | 0.2981 | 0.3632 | 0.2237 |
| 161 | S | 7 | 1 | 1 | 0 |  | 5.74 | 1 | 0.392 | 0.3925 | 0.2715 |
| 162 | S | 5 | 1 | 1 | 0 |  | 8.2 | 1 | 0.3478 | 0.3942 | 0.2641 |
| 163 | A | 4 | 1 | 1 | 0 |  | 11.17 | 1 | 0.2225 | 0.3561 | 0.2336 |
| 164 | N | 5 | 1 | 1 | 0 |  | 13.52 | 0 | 0.2282 | 0.3544 | 0.2331 |
| 165 | N | 1 | 1 | 1 | 0 |  | 17.53 | 0 | 0.1925 | 0.3269 | 0.2333 |
| 166 | C | 1 | 1 | 1 | 0 |  | 17.64 | 0 | 0.1969 | 0.2942 | 0.1984 |
| 167 | T | 1 | 1 | 1 | 0 |  | 21.76 | 0 | 0.188 | 0.2626 | 0.1992 |
| 168 | F | 2 | 1 | 1 | 0 |  | 22.43 | 0 | 0.1809 | 0.2287 | 0.187 |
| 169 | E | 4 | 1 | 1 | 0 |  | 21.29 | 0 | 0.1868 | 0.2357 | 0.1793 |
| 170 | Y | 5 | 1 | 1 | 0 |  | 24.55 | 1 | 0.1749 | 0.2211 | 0.1813 |
| 171 | V | 5 | 1 | 2 | 1 |  | 23.74 | 2 | 0.1735 | 0.2278 | 0.1855 |
| 172 | S | 7 | 1 | 1 | 0 |  | 27.57 | 2 | 0.1725 | 0.2179 | 0.1923 |
| 173 | Q | 6 | 1 | 1 | 0 |  | 29.72 | 2 | 0.2144 | 0.2266 | 0.2194 |
| 174 | P | 4 | 1 | 1 | 0 |  | 28.43 | 2 | 0.2039 | 0.2485 | 0.2182 |
| 175 | F | 2 | 1 | 1 | 0 |  | 25.8 | 4 | 0.2388 | 0.2806 | 0.243 |
| 176 | L | 6 | 2 | 2 | 0 |  | 23.86 | 4 | 0.2194 | 0.2977 | 0.2877 |
| 177 | M | 3 | 2 | 2 | 0 |  | 22.38 | 5 | 0.2085 | 0.2787 | 0.2758 |
| 178 | D | 4 | 1 | 1 | 0 |  | 22.04 | 7 | 0.2521 | 0.277 | 0.2935 |
| 179 | L | 5 | 1 | 1 | 0 |  | 17.75 | 7 | 0.2829 | 0.2664 | 0.2975 |
| 180 | E | 8 | 1 | 1 | 0 |  | 19.21 | 4 | 0.3033 | 0.2897 | 0.3024 |
| 181 | G | 6 | 2 | 2 | 0 |  | 19.13 | 3 | 0.3011 | 0.2938 | 0.3046 |
| 182 | K | 4 | 2 | 2 | 0 |  | 21.98 | 3 | 0.3383 | 0.3155 | 0.3105 |
| 183 | Q | 7 | 1 | 1 | 0 |  | 23.33 | 2 | 0.3733 | 0.3126 | 0.3331 |
| 184 | G | 2 | 2 | 2 | 0 |  | 26.62 | 2 | 0.355 | 0.3022 | 0.3454 |
| 185 | N | 4 | 3 | 3 | 0 |  | 28.45 | 2 | 0.2573 | 0.2892 | 0.317 |
| 186 | F | 1 | 2 | 2 | 0 |  | 24.66 | 3 | 0.2109 | 0.263 | 0.2673 |
| 187 | K | 7 | 1 | 1 | 0 |  | 29.44 | 3 | 0.2168 | 0.2579 | 0.2597 |
| 188 | N | 5 | 1 | 1 | 0 |  | 29.12 | 5 | 0.1853 | 0.2423 | 0.2228 |
| 189 | L | 2 | 1 | 1 | 0 |  | 99.999 | 5 | 0.1692 | 0.2337 | 0.205 |
| 190 | R | 1 | 2 | 2 | 0 |  | 26.44 | 5 | 0.1505 | 0.2246 | 0.1844 |
| 191 | E | 1 | 2 | 2 | 0 |  | 99.999 | 4 | 0.1411 | 0.1849 | 0.1752 |
| 192 | F | 3 | 2 | 2 | 0 |  | 29.53 | 3 | 0.1337 | 0.1743 | 0.1646 |
| 193 | V | 3 | 2 | 2 | 0 |  | 99.999 | 0 | 0.129 | 0.1611 | 0.1626 |
| 194 | F | 1 | 2 | 2 | 0 |  | 99.999 | 0 | 0.1243 | 0.1752 | 0.1611 |
| 195 | K | 2 | 1 | 1 | 0 |  | 99.999 | 0 | 0.1271 | 0.1702 | 0.1695 |
| 196 | N | 2 | 1 | 1 | 0 |  | 29.91 | 0 | 0.1305 | 0.1878 | 0.1832 |
| 197 | I | 6 | 2 | 2 | 0 |  | 34.3 | 0 | 0.1393 | 0.1929 | 0.1908 |
| 198 | D | 3 | 1 | 1 | 0 |  | 35.89 | 0 | 0.154 | 0.2337 | 0.2186 |
| 199 | G | 1 | 1 | 1 | 0 |  | 31.91 | 0 | 0.1493 | 0.246 | 0.212 |
| 200 | Y | 3 | 1 | 1 | 0 |  | 32.34 | 0 | 0.1382 | 0.1905 | 0.1769 |
| 201 | F | 3 | 1 | 1 | 0 |  | 99.999 | 0 | 0.1324 | 0.1785 | 0.1672 |
| 202 | K | 7 | 2 | 2 | 0 |  | 32.64 | 0 | 0.131 | 0.1514 | 0.1624 |
| 203 | I | 3 | 1 | 1 | 0 |  | 29.29 | 0 | 0.1289 | 0.1561 | 0.1586 |
| 204 | Y | 1 | 1 | 1 | 0 |  | 33.11 | 0 | 0.1318 | 0.1428 | 0.1589 |
| 205 | S | 8 | 1 | 1 | 0 |  | 34.06 | 0 | 0.1365 | 0.1569 | 0.1648 |
| 206 | K | 7 | 1 | 1 | 0 |  | 34.19 | 1 | 0.1476 | 0.1709 | 0.1761 |
| 207 | H | 3 | 1 | 1 | 0 |  | 31.87 | 1 | 0.1534 | 0.2051 | 0.1975 |
| 208 | T | 4 | 1 | 1 | 0 |  | 34.25 | 1 | 0.1832 | 0.2296 | 0.2108 |
| 209 | P | 3 | 1 | 1 | 0 |  | 35.18 | 2 | 0.1949 | 0.258 | 0.238 |
| 210 | I | 4 | 1 | 1 | 0 |  | 31.44 | 2 | 0.2152 | 0.2851 | 0.2534 |
| 211 | N | 3 | 1 | 2 | 1 |  | 33.48 | 1 | 0.2545 | 0.2891 | 0.2778 |
| 212 | L | 5 | 1 | 1 | 0 |  | 29.96 | 3 | 0.2439 | 0.2874 | 0.3083 |
| 213 | V | 7 | 2 | 2 | 0 |  | 27.33 | 6 | 0.2506 | 0.3102 | 0.3108 |
| 214 | R | 4 | 2 | 2 | 0 |  | 26.23 | 5 | 0.217 | 0.3058 | 0.312 |
| 215 | D | 3 | 4 | 4 | 0 |  | 27.82 | 5 | 0.1946 | 0.2753 | 0.29 |
| 216 | L | 3 | 2 | 2 | 0 |  | 29.58 | 7 | 0.1777 | 0.249 | 0.2526 |
| 217 | P | 1 | 1 | 1 | 0 |  | 32.63 | 6 | 0.1893 | 0.2384 | 0.26 |
| 218 | Q | 9 | 2 | 4 | 2 |  | 35.77 | 4 | 0.1959 | 0.2287 | 0.2782 |
| 219 | G | 2 | 1 | 1 | 0 |  | 99.999 | 4 | 0.2229 | 0.2102 | 0.2624 |
| 220 | F | 2 | 1 | 1 | 0 |  | 40.19 | 4 | 0.2046 | 0.1721 | 0.2162 |
| 221 | S | 4 | 3 | 4 | 1 |  | 38.71 | 2 | 0.1877 | 0.1596 | 0.195 |
| 222 | A | 6 | 1 | 1 | 0 |  | 39.91 | 1 | 0.1992 | 0.1424 | 0.1826 |
| 223 | L | 1 | 1 | 1 | 0 |  | 99.999 | 0 | 0.1468 | 0.136 | 0.1713 |
| 224 | E | 4 | 1 | 1 | 0 |  | 37.54 | 0 | 0.1483 | 0.1338 | 0.1695 |
| 225 | P | 1 | 1 | 1 | 0 |  | 37.6 | 0 | 0.1427 | 0.1295 | 0.1653 |
| 226 | L | 3 | 1 | 1 | 0 |  | 34.51 | 0 | 0.1442 | 0.1446 | 0.184 |
| 227 | V | 5 | 1 | 1 | 0 |  | 31.11 | 0 | 0.1591 | 0.1884 | 0.2084 |
| 228 | D | 4 | 1 | 1 | 0 |  | 33.19 | 0 | 0.1498 | 0.1613 | 0.18 |
| 229 | L | 2 | 1 | 1 | 0 |  | 99.999 | 0 | 0.1427 | 0.1859 | 0.1661 |
| 230 | P | 1 | 1 | 1 | 0 |  | 29.74 | 0 | 0.1417 | 0.1967 | 0.1743 |
| 231 | I | 4 | 2 | 2 | 0 |  | 26.9 | 0 | 0.1433 | 0.2106 | 0.1639 |
| 232 | G | 2 | 2 | 2 | 0 |  | 28.25 | 1 | 0.1481 | 0.2339 | 0.1694 |
| 233 | I | 2 | 2 | 2 | 0 |  | 24.2 | 2 | 0.1516 | 0.2515 | 0.1648 |
| 234 | N | 2 | 1 | 1 | 0 |  | 24.7 | 2 | 0.1466 | 0.2624 | 0.1778 |
| 235 | I | 2 | 1 | 1 | 0 |  | 24.98 | 2 | 0.1333 | 0.2472 | 0.1741 |
| 236 | T | 1 | 1 | 1 | 0 |  | 22.25 | 2 | 0.1392 | 0.2517 | 0.1893 |
| 237 | R | 6 | 1 | 1 | 0 |  | 16.04 | 3 | 0.1431 | 0.253 | 0.1947 |
| 238 | F | 4 | 1 | 1 | 0 |  | 99.999 | 2 | 0.1345 | 0.2406 | 0.183 |
| 239 | Q | 3 | 2 | 2 | 0 |  | 99.999 | 2 | 0.1448 | 0.246 | 0.1862 |
| 240 | T | 3 | 3 | 3 | 0 |  | 16.51 | 2 | 0.1524 | 0.2407 | 0.1855 |
| 241 | L | 4 | 2 | 2 | 0 |  | 18.24 | 2 | 0.1767 | 0.2498 | 0.1877 |
| 242 | L | 3 | 2 | 2 | 0 |  | 12.37 | 0 | 0.1931 | 0.2455 | 0.2094 |
| 243 | A | 3 | 2 | 2 | 0 |  | 13.97 | 0 | 0.2105 | 0.2891 | 0.2248 |
| 244 | L | 5 | 1 | 1 | 0 |  | 9.93 | 0 | 0.2484 | 0.3348 | 0.252 |
| 245 | H | 4 | 2 | 2 | 0 |  | 10.62 | 0 | 0.2637 | 0.3143 | 0.2713 |
| 246 | R | 5 | 2 | 2 | 0 |  | 7.13 | 0 | 0.2801 | 0.3803 | 0.3191 |
| 247 | S | 4 | 2 | 2 | 0 |  | 9.42 | 0 | 0.3029 | 0.4323 | 0.349 |
| 248 | Y | 4 | 1 | 1 | 0 |  | 99.999 | 0 | 0.3044 | 0.4372 | 0.4182 |
| 249 | L | 3 | 2 | 2 | 0 |  | 11.46 | 1 | 0.327 | 0.5154 | 0.4611 |
| 250 | T | 2 | 1 | 1 | 0 |  | 11.71 | 1 | 0.3355 | 0.6047 | 0.5645 |
| 251 | P | 2 | 1 | 1 | 0 |  | 12.98 | 2 | 0.3552 | 0.6947 | 0.712 |
| 252 | G | 2 | 1 | 1 | 0 |  | 11.69 | 2 | 0.3855 | 0.7566 | 0.848 |
| 253 | D | 10 | 1 | 3 | 2 |  | 9.95 | 8 | 0.3616 | 0.712 | 0.8684 |
| 254 | S | 9 | 2 | 3 | 1 |  | 7.04 | 7 | 0.3508 | 0.6158 | 0.7838 |
| 255 | S | 9 | 2 | 3 | 1 |  | 4.21 | 7 | 0.3609 | 0.5732 | 0.7984 |
| 256 | S | 7 | 1 | 1 | 0 |  | 5.09 | 6 | 0.334 | 0.5616 | 0.6505 |
| 257 | G | 7 | 1 | 1 | 0 |  | 6.97 | 6 | 0.3526 | 0.4616 | 0.4307 |
| 258 | W | 5 | 2 | 2 | 0 |  | 3.46 | 0 | 0.3224 | 0.391 | 0.3292 |
| 259 | T | 7 | 1 | 1 | 0 |  | 99.999 | 0 | 0.3133 | 0.3506 | 0.3091 |
| 260 | A | 5 | 1 | 2 | 1 |  | 99.999 | 0 | 0.2865 | 0.2662 | 0.2974 |
| 261 | G | 6 | 2 | 2 | 0 |  | 15.79 | 0 | 0.2601 | 0.3089 | 0.2851 |
| 262 | A | 7 | 2 | 2 | 0 |  | 19.3 | 0 | 0.2494 | 0.3614 | 0.2852 |
| 263 | A | 3 | 2 | 2 | 0 |  | 99.999 | 0 | 0.2362 | 0.3474 | 0.2628 |
| 264 | A | 2 | 1 | 1 | 0 |  | 99.999 | 0 | 0.185 | 0.312 | 0.234 |
| 265 | Y | 1 | 1 | 1 | 0 |  | 18.25 | 0 | 0.1518 | 0.2991 | 0.2129 |
| 266 | Y | 3 | 1 | 1 | 0 |  | 99.999 | 0 | 0.1425 | 0.2485 | 0.2114 |
| 267 | V | 3 | 1 | 1 | 0 |  | 23.74 | 1 | 0.1368 | 0.2405 | 0.2032 |
| 268 | G | 1 | 1 | 1 | 0 |  | 99.999 | 1 | 0.1428 | 0.2332 | 0.2124 |
| 269 | Y | 3 | 1 | 1 | 0 |  | 23.46 | 2 | 0.1311 | 0.2271 | 0.2022 |
| 270 | L | 1 | 1 | 1 | 0 |  | 99.999 | 2 | 0.1317 | 0.2024 | 0.1958 |
| 271 | Q | 2 | 2 | 2 | 0 |  | 33.29 | 2 | 0.1367 | 0.21 | 0.2086 |
| 272 | P | 4 | 1 | 2 | 1 |  | 36.37 | 2 | 0.1404 | 0.2105 | 0.2154 |
| 273 | R | 6 | 1 | 1 | 0 |  | 36.92 | 3 | 0.1394 | 0.1987 | 0.2194 |
| 274 | T | 1 | 1 | 1 | 0 |  | 43.3 | 2 | 0.1503 | 0.1939 | 0.2349 |
| 275 | F | 2 | 1 | 1 | 0 |  | 99.999 | 2 | 0.1412 | 0.1631 | 0.2007 |
| 276 | L | 2 | 1 | 1 | 0 |  | 47.1 | 2 | 0.1463 | 0.1442 | 0.1691 |
| 277 | L | 3 | 1 | 1 | 0 |  | 99.999 | 2 | 0.1477 | 0.123 | 0.162 |
| 278 | K | 6 | 1 | 2 | 1 |  | 49.5 | 0 | 0.1904 | 0.1252 | 0.155 |
| 279 | Y | 2 | 1 | 1 | 0 |  | 47 | 0 | 0.2146 | 0.1198 | 0.1687 |
| 280 | N | 2 | 1 | 1 | 0 |  | 52.29 | 0 | 0.2595 | 0.137 | 0.1903 |
| 281 | E | 6 | 2 | 3 | 1 |  | 55.32 | 2 | 0.3227 | 0.1608 | 0.2134 |
| 282 | N | 1 | 1 | 1 | 0 |  | 51.92 | 4 | 0.3194 | 0.1632 | 0.287 |
| 283 | G | 1 | 1 | 1 | 0 |  | 49.5 | 5 | 0.3003 | 0.1467 | 0.1977 |
| 284 | T | 1 | 1 | 1 | 0 |  | 47.64 | 5 | 0.2317 | 0.1284 | 0.1839 |
| 285 | I | 2 | 1 | 1 | 0 |  | 99.999 | 6 | 0.1772 | 0.1195 | 0.1745 |
| 286 | T | 7 | 1 | 1 | 0 |  | 47.71 | 5 | 0.1775 | 0.1391 | 0.1825 |
| 287 | D | 2 | 1 | 1 | 0 |  | 47.23 | 4 | 0.1871 | 0.1378 | 0.174 |
| 288 | A | 1 | 2 | 2 | 0 |  | 44.91 | 2 | 0.1555 | 0.136 | 0.1702 |
| 289 | V | 2 | 2 | 2 | 0 |  | 45.39 | 2 | 0.1551 | 0.149 | 0.1702 |
| 290 | D | 1 | 2 | 2 | 0 |  | 40.09 | 1 | 0.1468 | 0.1723 | 0.1843 |
| 291 | C | 1 | 1 | 1 | 0 |  | 44.94 | 0 | 0.1512 | 0.1791 | 0.2377 |
| 292 | A | 2 | 2 | 3 | 1 |  | 42.44 | 0 | 0.158 | 0.1888 | 0.2709 |
| 293 | L | 2 | 2 | 2 | 0 |  | 40.21 | 0 | 0.1642 | 0.2408 | 0.2582 |
| 294 | D | 3 | 2 | 2 | 0 |  |  | 0 | 0.1693 | 0.16 | 0.204 |
| 295 | P | 1 | 2 | 2 | 0 |  |  | 0 | 0.1619 | 0.1242 | 0.1734 |
| 296 | L | 1 | 2 | 2 | 0 |  |  | 0 | 0.1612 | 0.126 | 0.1678 |
| 297 | S | 2 | 2 | 2 | 0 |  |  | 0 | 0.1556 | 0.126 | 0.181 |
| 298 | E | 1 | 2 | 2 | 0 |  |  | 0 | 0.1521 | 0.1154 | 0.1749 |
| 299 | T | 2 | 1 | 1 | 0 |  |  | 0 | 0.156 | 0.1121 | 0.1574 |
| 300 | K | 1 | 1 | 1 | 0 |  |  | 0 | 0.1575 | 0.1266 | 0.1601 |
| 301 | C | 1 | 1 | 1 | 0 |  |  | 0 | 0.1535 | 0.1342 | 0.1723 |
| 302 | T | 2 | 1 | 1 | 0 |  |  | 0 | 0.1643 | 0.1312 | 0.1713 |
| 303 | L | 4 | 1 | 1 | 0 |  |  | 0 | 0.1631 | 0.1346 | 0.1476 |
| 304 | K | 1 | 1 | 1 | 0 |  |  | 0 | 0.1687 | 0.1942 | 0.1493 |
| 305 | S | 4 | 1 | 1 | 0 |  |  | 0 | 0.2062 | 0.1741 | 0.1704 |
| 306 | F | 3 | 1 | 2 | 1 |  |  | 0 | 0.2899 | 0.2262 | 0.1657 |
| 307 | T | 6 | 3 | 3 | 0 |  |  | 0 | 0.3181 | 0.2432 | 0.1759 |
| 308 | V | 2 | 2 | 2 | 0 |  |  | 0 | 0.2564 | 0.1593 | 0.1681 |
| 309 | E | 7 | 2 | 2 | 0 |  |  | 0 | 0.2236 | 0.1425 | 0.1495 |
| 310 | K | 1 | 1 | 1 | 0 |  |  | 0 | 0.2126 | 0.1276 | 0.157 |
| 311 | G | 1 | 1 | 1 | 0 |  |  | 0 | 0.1601 | 0.1191 | 0.1557 |
| 312 | I | 1 | 1 | 1 | 0 |  |  | 0 | 0.1468 | 0.1076 | 0.1282 |
| 313 | Y | 1 | 1 | 1 | 0 |  |  | 0 | 0.1371 | 0.106 | 0.1311 |
| 314 | Q | 1 | 1 | 1 | 0 |  |  | 0 | 0.1454 | 0.1062 | 0.1335 |
| 315 | T | 1 | 1 | 1 | 0 |  |  | 0 | 0.1397 | 0.112 | 0.1642 |
| 316 | S | 1 | 1 | 1 | 0 |  |  | 0 | 0.158 | 0.1136 | 0.1774 |
| 317 | N | 1 | 1 | 1 | 0 |  |  | 0 | 0.1352 | 0.1096 | 0.152 |
| 318 | F | 1 | 2 | 2 | 0 |  |  | 0 | 0.1376 | 0.1312 | 0.1739 |
| 319 | R | 1 | 1 | 1 | 0 |  |  | 0 | 0.1558 | 0.1312 | 0.1505 |
| 320 | V | 1 | 1 | 1 | 0 |  |  | 2 | 0.1631 | 0.1388 | 0.1677 |
| 321 | Q | 5 | 2 | 2 | 0 |  |  | 2 | 0.1792 | 0.1983 | 0.1905 |
| 322 | P | 1 | 1 | 1 | 0 |  |  | 3 | 0.161 | 0.18 | 0.1965 |
| 323 | T | 3 | 2 | 2 | 0 |  |  | 3 | 0.215 | 0.2109 | 0.2398 |
| 324 | E | 10 | 1 | 3 | 2 |  |  | 4 | 0.1558 | 0.1937 | 0.2139 |
| 325 | S | 3 | 1 | 1 | 0 |  |  | 3 | 0.1378 | 0.1878 | 0.1843 |
| 326 | I | 2 | 1 | 1 | 0 |  |  | 3 | 0.1251 | 0.1749 | 0.1686 |
| 327 | V | 3 | 1 | 1 | 0 |  |  | 2 | 0.1408 | 0.1701 | 0.1445 |
| 328 | R | 1 | 1 | 1 | 0 |  |  | 3 | 0.1476 | 0.1655 | 0.1511 |
| 329 | F | 1 | 1 | 1 | 0 |  |  | 3 | 0.1673 | 0.1773 | 0.1521 |
| 330 | P | 1 | 1 | 1 | 0 |  |  | 3 | 0.1879 | 0.1835 | 0.1723 |
| 331 | N | 1 | 1 | 1 | 0 |  |  | 3 | 0.188 | 0.1873 | 0.2184 |
| 332 | I | 1 | 1 | 1 | 0 |  |  | 3 | 0.1598 | 0.1879 | 0.1933 |
| 333 | T | 1 | 1 | 1 | 0 |  |  | 2 | 0.1627 | 0.1922 | 0.2124 |
| 334 | N | 2 | 1 | 1 | 0 |  |  | 1 | 0.1808 | 0.1925 | 0.1948 |
| 335 | L | 3 | 1 | 1 | 0 |  |  | 0 | 0.2152 | 0.1859 | 0.1751 |
| 336 | C | 1 | 1 | 1 | 0 | 33.399 |  | 0 | 0.1986 | 0.1776 | 0.1487 |
| 337 | P | 1 | 1 | 1 | 0 | 31.782 |  | 0 | 0.3027 | 0.1883 | 0.1699 |
| 338 | F | 1 | 2 | 2 | 0 | 28.855 |  | 2 | 0.2421 | 0.189 | 0.1379 |
| 339 | G | 4 | 1 | 1 | 0 | 28.188 |  | 4 | 0.2459 | 0.2118 | 0.1501 |
| 340 | E | 5 | 1 | 1 | 0 | 28.158 |  | 4 | 0.2885 | 0.2139 | 0.1643 |
| 341 | V | 2 | 2 | 2 | 0 | 25.215 |  | 4 | 0.2481 | 0.2081 | 0.1539 |
| 342 | F | 1 | 1 | 1 | 0 | 23.338 |  | 4 | 0.2248 | 0.2296 | 0.1471 |
| 343 | N | 1 | 1 | 1 | 0 | 22.845 |  | 3 | 0.2688 | 0.2524 | 0.1618 |
| 344 | A | 3 | 2 | 2 | 0 | 23.148 |  | 1 | 0.2683 | 0.2566 | 0.1696 |
| 345 | T | 2 | 1 | 2 | 1 | 20.476 |  | 1 | 0.2913 | 0.3021 | 0.2115 |
| 346 | R | 4 | 1 | 3 | 2 | 19.957 |  | 1 | 0.2838 | 0.2671 | 0.1955 |
| 347 | F | 1 | 1 | 1 | 0 | 18.551 |  | 3 | 0.2345 | 0.2107 | 0.1433 |
| 348 | A | 2 | 2 | 2 | 0 | 17.345 |  | 4 | 0.2272 | 0.1864 | 0.1392 |
| 349 | S | 2 | 1 | 1 | 0 | 14.869 |  | 4 | 0.2383 | 0.1802 | 0.139 |
| 350 | V | 1 | 1 | 1 | 0 | 12.217 |  | 4 | 0.2303 | 0.1707 | 0.1306 |
| 351 | Y | 1 | 1 | 1 | 0 | 14.242 |  | 4 | 0.2488 | 0.1728 | 0.1362 |
| 352 | A | 1 | 1 | 1 | 0 | 17.146 |  | 2 | 0.25 | 0.18 | 0.1544 |
| 353 | W | 1 | 2 | 2 | 0 | 18.017 |  | 0 | 0.2278 | 0.1686 | 0.1606 |
| 354 | N | 2 | 3 | 3 | 0 | 22.574 |  | 0 | 0.2227 | 0.1729 | 0.1461 |
| 355 | R | 1 | 1 | 1 | 0 | 25.312 |  | 0 | 0.2255 | 0.1613 | 0.1461 |
| 356 | K | 5 | 1 | 1 | 0 | 28.303 |  | 1 | 0.2147 | 0.1742 | 0.1215 |
| 357 | R | 2 | 1 | 1 | 0 | 31.556 |  | 1 | 0.216 | 0.1795 | 0.1343 |
| 358 | I | 1 | 1 | 1 | 0 | 32.678 |  | 1 | 0.1976 | 0.181 | 0.13 |
| 359 | S | 2 | 2 | 2 | 0 | 36.783 |  | 1 | 0.206 | 0.1855 | 0.1282 |
| 360 | N | 3 | 1 | 1 | 0 | 40.204 |  | 1 | 0.1799 | 0.1823 | 0.1477 |
| 361 | C | 1 | 1 | 1 | 0 | 37.277 |  | 2 | 0.1905 | 0.181 | 0.1512 |
| 362 | V | 2 | 1 | 1 | 0 | 38.067 |  | 2 | 0.1698 | 0.1851 | 0.1535 |
| 363 | A | 2 | 1 | 1 | 0 | 35.288 |  | 3 | 0.1899 | 0.1867 | 0.1509 |
| 364 | D | 1 | 2 | 2 | 0 | 31.359 |  | 7 | 0.1902 | 0.2055 | 0.1525 |
| 365 | Y | 1 | 1 | 1 | 0 | 28.423 |  | 8 | 0.1897 | 0.2662 | 0.1502 |
| 366 | S | 2 | 1 | 1 | 0 | 28.341 |  | 6 | 0.1891 | 0.2703 | 0.1596 |
| 367 | V | 3 | 2 | 2 | 0 | 25.423 |  | 6 | 0.1907 | 0.2861 | 0.1503 |
| 368 | L | 2 | 1 | 1 | 0 | 25.157 |  | 6 | 0.1811 | 0.2721 | 0.146 |
| 369 | Y | 1 | 1 | 1 | 0 | 23.683 |  | 2 | 0.1641 | 0.2563 | 0.1611 |
| 370 | N | 1 | 1 | 1 | 0 | 22.676 |  | 2 | 0.173 | 0.2695 | 0.1685 |
| 371 | S | 1 | 1 | 1 | 0 | 18.918 |  | 3 | 0.1829 | 0.3188 | 0.154 |
| 372 | A | 3 | 2 | 3 | 1 | 15.105 |  | 3 | 0.1896 | 0.3577 | 0.1623 |
| 373 | S | 3 | 2 | 2 | 0 | 16.567 |  | 2 | 0.2092 | 0.3039 | 0.1525 |
| 374 | F | 1 | 2 | 2 | 0 | 15.152 |  | 2 | 0.1922 | 0.2305 | 0.1425 |
| 375 | S | 1 | 1 | 1 | 0 | 13.944 |  | 1 | 0.1948 | 0.2105 | 0.1443 |
| 376 | T | 1 | 1 | 1 | 0 | 16.929 |  | 1 | 0.1809 | 0.1917 | 0.1296 |
| 377 | F | 1 | 1 | 1 | 0 | 20.197 |  | 1 | 0.1711 | 0.1814 | 0.1309 |
| 378 | K | 3 | 2 | 2 | 0 | 20.825 |  | 1 | 0.1595 | 0.1761 | 0.1333 |
| 379 | C | 1 | 1 | 1 | 0 | 26.106 |  | 1 | 0.1751 | 0.1703 | 0.1255 |
| 380 | Y | 1 | 1 | 1 | 0 | 21.418 |  | 1 | 0.202 | 0.159 | 0.1236 |
| 381 | G | 1 | 1 | 1 | 0 | 29.948 |  | 0 | 0.2374 | 0.1601 | 0.1269 |
| 382 | V | 1 | 1 | 1 | 0 | 31.247 |  | 0 | 0.2305 | 0.1583 | 0.12 |
| 383 | S | 1 | 1 | 1 | 0 | 31.246 |  | 0 | 0.2169 | 0.1957 | 0.1228 |
| 384 | P | 2 | 2 | 2 | 0 | 27.71 |  | 1 | 0.1819 | 0.203 | 0.1453 |
| 385 | T | 3 | 1 | 1 | 0 | 28.712 |  | 1 | 0.1901 | 0.1968 | 0.1494 |
| 386 | K | 1 | 1 | 1 | 0 | 32.614 |  | 1 | 0.2028 | 0.1748 | 0.1315 |
| 387 | L | 1 | 1 | 1 | 0 | 30.719 |  | 1 | 0.1894 | 0.1881 | 0.1295 |
| 388 | N | 2 | 1 | 1 | 0 | 34.266 |  | 1 | 0.1874 | 0.2122 | 0.1413 |
| 389 | D | 1 | 1 | 1 | 0 | 37.888 |  | 1 | 0.2148 | 0.2148 | 0.1357 |
| 390 | L | 1 | 1 | 1 | 0 | 39.685 |  | 0 | 0.2056 | 0.209 | 0.1281 |
| 391 | C | 1 | 1 | 1 | 0 | 36.86 |  | 0 | 0.2065 | 0.1958 | 0.1286 |
| 392 | F | 1 | 1 | 1 | 0 | 38.832 |  | 0 | 0.2086 | 0.1871 | 0.1174 |
| 393 | T | 2 | 1 | 1 | 0 | 36.581 |  | 0 | 0.2416 | 0.1932 | 0.1236 |
| 394 | N | 2 | 1 | 1 | 0 | 33.931 |  | 0 | 0.2424 | 0.1828 | 0.1228 |
| 395 | V | 1 | 1 | 1 | 0 | 32.013 |  | 0 | 0.2121 | 0.1729 | 0.1198 |
| 396 | Y | 1 | 1 | 1 | 0 | 28.421 |  | 0 | 0.2052 | 0.1681 | 0.1304 |
| 397 | A | 2 | 1 | 1 | 0 | 26.452 |  | 0 | 0.2002 | 0.1657 | 0.1287 |
| 398 | D | 1 | 1 | 1 | 0 | 99.999 |  | 0 | 0.1952 | 0.1504 | 0.1178 |
| 399 | S | 3 | 1 | 1 | 0 | 20.364 |  | 0 | 0.1937 | 0.1611 | 0.1172 |
| 400 | F | 1 | 1 | 1 | 0 | 16.039 |  | 0 | 0.1982 | 0.1604 | 0.1136 |
| 401 | V | 2 | 1 | 1 | 0 | 99.999 |  | 3 | 0.2053 | 0.1708 | 0.1223 |
| 402 | I | 2 | 1 | 2 | 1 | 99.999 |  | 3 | 0.2155 | 0.1672 | 0.1245 |
| 403 | R | 3 | 1 | 3 | 2 | 4.716 |  | 3 | 0.2228 | 0.1761 | 0.1326 |
| 404 | G | 3 | 1 | 1 | 0 | 99.999 |  | 4 | 0.223 | 0.1894 | 0.1517 |
| 405 | D | 2 | 2 | 2 | 0 | 7.231 |  | 5 | 0.2373 | 0.1716 | 0.2486 |
| 406 | E | 2 | 1 | 1 | 0 | 6.943 |  | 4 | 0.2228 | 0.1458 | 0.1866 |
| 407 | V | 1 | 1 | 1 | 0 | 13.831 |  | 4 | 0.1998 | 0.1537 | 0.1925 |
| 408 | R | 1 | 3 | 3 | 0 | 10.232 |  | 4 | 0.2222 | 0.1533 | 0.1754 |
| 409 | Q | 1 | 2 | 2 | 0 | 10.064 |  | 3 | 0.2293 | 0.1296 | 0.134 |
| 410 | I | 2 | 1 | 1 | 0 | 16.059 |  | 2 | 0.2116 | 0.1212 | 0.1383 |
| 411 | A | 1 | 1 | 1 | 0 | 18.936 |  | 0 | 0.219 | 0.1361 | 0.1349 |
| 412 | P | 1 | 1 | 1 | 0 | 20.244 |  | 0 | 0.249 | 0.1425 | 0.1522 |
| 413 | G | 2 | 1 | 1 | 0 | 18.486 |  | 1 | 0.295 | 0.1915 | 0.2373 |
| 414 | Q | 2 | 3 | 3 | 0 | 14.568 |  | 3 | 0.2778 | 0.1697 | 0.2063 |
| 415 | T | 1 | 1 | 1 | 0 | 13.24 |  | 3 | 0.2855 | 0.1453 | 0.1661 |
| 416 | G | 1 | 1 | 1 | 0 | 10.126 |  | 3 | 0.2736 | 0.1415 | 0.1557 |
| 417 | K | 2 | 2 | 3 | 1 | 5.672 |  | 6 | 0.2746 | 0.1398 | 0.153 |
| 418 | I | 1 | 1 | 1 | 0 | 9.366 |  | 7 | 0.2529 | 0.146 | 0.1395 |
| 419 | A | 1 | 1 | 1 | 0 | 99.999 |  | 4 | 0.2556 | 0.1426 | 0.138 |
| 420 | D | 1 | 1 | 1 | 0 | 12.057 |  | 5 | 0.2723 | 0.1357 | 0.1444 |
| 421 | Y | 1 | 1 | 1 | 0 | 7.678 |  | 5 | 0.2697 | 0.1371 | 0.1367 |
| 422 | N | 1 | 1 | 1 | 0 | 10.446 |  | 2 | 0.2462 | 0.1385 | 0.1307 |
| 423 | Y | 1 | 1 | 1 | 0 | 14.825 |  | 1 | 0.2282 | 0.1365 | 0.1318 |
| 424 | K | 2 | 1 | 1 | 0 | 17.413 |  | 1 | 0.2363 | 0.1455 | 0.1223 |
| 425 | L | 1 | 1 | 1 | 0 | 19.63 |  | 4 | 0.2342 | 0.1473 | 0.1225 |
| 426 | P | 1 | 1 | 1 | 0 | 23.022 |  | 4 | 0.2541 | 0.1706 | 0.1483 |
| 427 | D | 1 | 1 | 1 | 0 | 23.869 |  | 5 | 0.2683 | 0.1795 | 0.1561 |
| 428 | D | 2 | 1 | 1 | 0 | 27.147 |  | 6 | 0.3153 | 0.1705 | 0.1548 |
| 429 | F | 1 | 1 | 1 | 0 | 29.353 |  | 6 | 0.2525 | 0.1583 | 0.1293 |
| 430 | T | 4 | 1 | 1 | 0 | 25.388 |  | 3 | 0.2376 | 0.1583 | 0.1252 |
| 431 | G | 1 | 1 | 1 | 0 | 99.999 |  | 3 | 0.2013 | 0.1587 | 0.1223 |
| 432 | C | 1 | 2 | 2 | 0 | 99.999 |  | 1 | 0.171 | 0.1663 | 0.119 |
| 433 | V | 1 | 1 | 1 | 0 | 21.368 |  | 0 | 0.1628 | 0.1628 | 0.1148 |
| 434 | I | 2 | 2 | 2 | 0 | 99.999 |  | 0 | 0.1647 | 0.1753 | 0.1191 |
| 435 | A | 1 | 2 | 2 | 0 | 99.999 |  | 0 | 0.1749 | 0.1776 | 0.1245 |
| 436 | W | 1 | 2 | 2 | 0 | 15.373 |  | 0 | 0.188 | 0.1975 | 0.1375 |
| 437 | N | 1 | 2 | 2 | 0 | 10.372 |  | 1 | 0.2095 | 0.2141 | 0.1505 |
| 438 | S | 2 | 3 | 3 | 0 | 13.148 |  | 3 | 0.2313 | 0.2279 | 0.1761 |
| 439 | N | 5 | 3 | 4 | 1 | 9 |  | 3 | 0.2569 | 0.262 | 0.2037 |
| 440 | N | 5 | 2 | 4 | 2 | 11.072 |  | 4 | 0.2851 | 0.4209 | 0.2641 |
| 441 | L | 6 | 2 | 4 | 2 | 15.564 |  | 4 | 0.2782 | 0.441 | 0.3106 |
| 442 | D | 1 | 1 | 1 | 0 | 12.37 |  | 3 | 0.2794 | 0.4542 | 0.3246 |
| 443 | S | 8 | 1 | 2 | 1 | 9.908 |  | 1 | 0.3139 | 0.5026 | 0.3685 |
| 444 | K | 5 | 1 | 2 | 1 | 8.544 |  | 1 | 0.3451 | 0.4223 | 0.4241 |
| 445 | V | 8 | 1 | 3 | 2 | 5.738 |  | 1 | 0.4202 | 0.3735 | 0.5345 |
| 446 | G | 4 | 2 | 2 | 0 | 4.765 |  | 2 | 0.4586 | 0.3823 | 0.4602 |
| 447 | G | 3 | 1 | 2 | 1 | 6.181 |  | 3 | 0.4461 | 0.3212 | 0.3478 |
| 448 | N | 2 | 1 | 2 | 1 | 6.329 |  | 3 | 0.3744 | 0.3531 | 0.2511 |
| 449 | Y | 4 | 1 | 3 | 2 | 3.235 |  | 3 | 0.3965 | 0.2605 | 0.2021 |
| 450 | N | 5 | 1 | 2 | 1 | 10.176 |  | 2 | 0.3622 | 0.2799 | 0.2058 |
| 451 | Y | 2 | 1 | 1 | 0 | 9.612 |  | 1 | 0.2741 | 0.2181 | 0.1439 |
| 452 | L | 4 | 2 | 2 | 0 | 9.117 |  | 0 | 0.267 | 0.2029 | 0.1375 |
| 453 | Y | 3 | 1 | 1 | 0 | 3.414 |  | 0 | 0.2558 | 0.1642 | 0.1319 |
| 454 | R | 1 | 1 | 1 | 0 | 8.322 |  | 1 | 0.2719 | 0.157 | 0.127 |
| 455 | L | 6 | 1 | 1 | 0 | 3.913 |  | 1 | 0.2866 | 0.1598 | 0.1329 |
| 456 | F | 6 | 1 | 1 | 0 | 4.042 |  | 1 | 0.3147 | 0.1784 | 0.1484 |
| 457 | R | 2 | 1 | 1 | 0 | 8.176 |  | 2 | 0.3219 | 0.1816 | 0.1493 |
| 458 | K | 4 | 3 | 3 | 0 | 9.53 |  | 5 | 0.3633 | 0.2146 | 0.1609 |
| 459 | S | 5 | 1 | 2 | 1 | 11.752 |  | 4 | 0.3243 | 0.1981 | 0.1808 |
| 460 | N | 2 | 2 | 3 | 1 | 13.198 |  | 5 | 0.299 | 0.1615 | 0.1549 |
| 461 | L | 2 | 1 | 1 | 0 | 15.763 |  | 5 | 0.2764 | 0.1444 | 0.1432 |
| 462 | K | 3 | 1 | 1 | 0 | 19.22 |  | 4 | 0.2708 | 0.1655 | 0.1487 |
| 463 | P | 1 | 1 | 1 | 0 | 22.224 |  | 1 | 0.2376 | 0.1656 | 0.1409 |
| 464 | F | 2 | 1 | 1 | 0 | 22.255 |  | 2 | 0.2308 | 0.1689 | 0.152 |
| 465 | E | 2 | 1 | 1 | 0 | 19.517 |  | 1 | 0.2602 | 0.1663 | 0.1368 |
| 466 | R | 1 | 1 | 1 | 0 | 20.081 |  | 2 | 0.263 | 0.1637 | 0.141 |
| 467 | D | 1 | 2 | 2 | 0 | 15.514 |  | 2 | 0.2765 | 0.1641 | 0.1426 |
| 468 | I | 2 | 3 | 3 | 0 | 16.49 |  | 2 | 0.3102 | 0.1912 | 0.1564 |
| 469 | S | 2 | 1 | 1 | 0 | 14.662 |  | 2 | 0.3199 | 0.2065 | 0.1725 |
| 470 | T | 3 | 1 | 1 | 0 | 11.514 |  | 2 | 0.3392 | 0.2177 | 0.1499 |
| 471 | E | 3 | 1 | 1 | 0 | 11.66 |  | 2 | 0.3911 | 0.2272 | 0.1582 |
| 472 | I | 5 | 2 | 2 | 0 | 10.138 |  | 2 | 0.3945 | 0.2355 | 0.1416 |
| 473 | Y | 3 | 1 | 1 | 0 | 4.915 |  | 4 | 0.3912 | 0.2611 | 0.1356 |
| 474 | Q | 4 | 1 | 1 | 0 | 6.673 |  | 4 | 0.4417 | 0.3187 | 0.161 |
| 475 | A | 4 | 2 | 2 | 0 | 3.225 |  | 4 | 0.4774 | 0.379 | 0.1644 |
| 476 | G | 5 | 2 | 2 | 0 | 2.386 |  | 4 | 0.5405 | 0.4739 | 0.2181 |
| 477 | S | 3 | 2 | 2 | 0 | 4.48 |  | 4 | 0.5955 | 0.5351 | 0.289 |
| 478 | T | 5 | 1 | 2 | 1 | 5.935 |  | 2 | 0.5698 | 0.493 | 0.271 |
| 479 | P | 4 | 1 | 1 | 0 | 9.789 |  | 1 | 0.57 | 0.4389 | 0.2573 |
| 480 | C | 2 | 1 | 1 | 0 | 10.749 |  | 3 | 0.5233 | 0.371 | 0.2182 |
| 481 | N | 4 | 2 | 2 | 0 | 14.144 |  | 3 | 0.5714 | 0.4012 | 0.2588 |
| 482 | G | 4 | 1 | 1 | 0 | 11.808 |  | 6 | 0.557 | 0.3993 | 0.2584 |
| 483 | V | 3 | 3 | 4 | 1 | 8.401 |  | 7 | 0.5566 | 0.3862 | 0.2604 |
| 484 | E | 6 | 1 | 3 | 2 | 6.803 |  | 7 | 0.5047 | 0.3519 | 0.2207 |
| 485 | G | 3 | 1 | 1 | 0 | 5.231 |  | 5 | 0.523 | 0.3968 | 0.2284 |
| 486 | F | 5 | 1 | 2 | 1 | 3.197 |  | 4 | 0.5251 | 0.4345 | 0.2249 |
| 487 | N | 3 | 1 | 1 | 0 | 2.853 |  | 1 | 0.4816 | 0.3919 | 0.1929 |
| 488 | C | 3 | 1 | 1 | 0 | 99.999 |  | 1 | 0.4442 | 0.3296 | 0.1578 |
| 489 | Y | 4 | 1 | 1 | 0 | 2.58 |  | 1 | 0.4142 | 0.2785 | 0.1379 |
| 490 | F | 7 | 1 | 2 | 1 | 5.287 |  | 1 | 0.3746 | 0.2375 | 0.1364 |
| 491 | P | 2 | 3 | 3 | 0 | 7.157 |  | 1 | 0.3154 | 0.188 | 0.1289 |
| 492 | L | 1 | 1 | 1 | 0 | 6.147 |  | 1 | 0.2965 | 0.1884 | 0.1317 |
| 493 | Q | 8 | 1 | 3 | 2 | 2.602 |  | 1 | 0.2904 | 0.1821 | 0.1399 |
| 494 | S | 6 | 2 | 3 | 1 | 5.684 |  | 2 | 0.2865 | 0.198 | 0.1637 |
| 495 | Y | 1 | 1 | 1 | 0 | 3.846 |  | 2 | 0.2659 | 0.1795 | 0.1879 |
| 496 | G | 2 | 1 | 1 | 0 | 3.625 |  | 2 | 0.2874 | 0.2085 | 0.221 |
| 497 | F | 1 | 1 | 1 | 0 | 5.516 |  | 3 | 0.2703 | 0.2165 | 0.1937 |
| 498 | Q | 6 | 1 | 3 | 2 | 3.76 |  | 3 | 0.298 | 0.2584 | 0.2239 |
| 499 | P | 4 | 1 | 1 | 0 | 5.865 |  | 1 | 0.3039 | 0.2712 | 0.2298 |
| 500 | T | 4 | 1 | 1 | 0 | 2.724 |  | 2 | 0.3426 | 0.3255 | 0.2593 |
| 501 | N | 7 | 1 | 3 | 2 | 3.326 |  | 2 | 0.3202 | 0.3642 | 0.2292 |
| 502 | G | 2 | 1 | 1 | 0 | 3.295 |  | 1 | 0.3132 | 0.3342 | 0.2108 |
| 503 | V | 3 | 2 | 2 | 0 | 5.028 |  | 3 | 0.2666 | 0.2744 | 0.2274 |
| 504 | G | 5 | 1 | 2 | 1 | 5.952 |  | 3 | 0.2684 | 0.2442 | 0.1893 |
| 505 | Y | 3 | 1 | 2 | 1 | 3.465 |  | 2 | 0.266 | 0.2297 | 0.1817 |
| 506 | Q | 1 | 1 | 1 | 0 | 6.573 |  | 3 | 0.2468 | 0.2035 | 0.165 |
| 507 | P | 2 | 1 | 1 | 0 | 99.999 |  | 3 | 0.2307 | 0.1939 | 0.1589 |
| 508 | Y | 3 | 2 | 3 | 1 | 10.768 |  | 1 | 0.203 | 0.1859 | 0.1357 |
| 509 | R | 1 | 2 | 2 | 0 | 16.008 |  | 1 | 0.1927 | 0.1809 | 0.1304 |
| 510 | V | 1 | 2 | 2 | 0 | 99.999 |  | 1 | 0.1796 | 0.1625 | 0.1167 |
| 511 | V | 1 | 1 | 1 | 0 | 21.685 |  | 0 | 0.1766 | 0.1636 | 0.1167 |
| 512 | V | 1 | 1 | 1 | 0 | 21.357 |  | 0 | 0.1765 | 0.1535 | 0.1149 |
| 513 | L | 1 | 1 | 1 | 0 | 25.87 |  | 1 | 0.1842 | 0.1649 | 0.1238 |
| 514 | S | 1 | 1 | 1 | 0 | 28.094 |  | 1 | 0.2085 | 0.165 | 0.1228 |
| 515 | F | 1 | 1 | 1 | 0 | 30.952 |  | 2 | 0.215 | 0.1797 | 0.1243 |
| 516 | E | 1 | 2 | 2 | 0 | 30.087 |  | 2 | 0.2415 | 0.1919 | 0.1266 |
| 517 | L | 1 | 1 | 1 | 0 | 36.133 |  | 3 | 0.2412 | 0.2638 | 0.1295 |
| 518 | L | 1 | 1 | 1 | 0 | 39.457 |  | 2 | 0.2085 | 0.2677 | 0.1496 |
| 519 | H | 2 | 3 | 4 | 1 |  |  | 3 | 0.2167 | 0.3181 | 0.1666 |
| 520 | A | 2 | 2 | 3 | 1 |  |  | 2 | 0.212 | 0.2924 | 0.1555 |
| 521 | P | 1 | 1 | 1 | 0 |  |  | 2 | 0.2006 | 0.251 | 0.1636 |
| 522 | A | 1 | 3 | 3 | 0 |  |  | 1 | 0.2092 | 0.2035 | 0.1352 |
| 523 | T | 1 | 1 | 1 | 0 |  |  | 1 | 0.1831 | 0.179 | 0.139 |
| 524 | V | 1 | 1 | 1 | 0 |  |  | 1 | 0.1895 | 0.1654 | 0.1256 |
| 525 | C | 1 | 1 | 1 | 0 |  |  | 1 | 0.189 | 0.1693 | 0.1353 |
| 526 | G | 1 | 1 | 1 | 0 |  |  | 1 | 0.1878 | 0.1837 | 0.1451 |
| 527 | P | 1 | 1 | 1 | 0 |  |  | 1 | 0.1853 | 0.2002 | 0.1716 |
| 528 | K | 1 | 1 | 1 | 0 |  |  | 1 | 0.1847 | 0.1992 | 0.1857 |
| 529 | K | 3 | 2 | 3 | 1 |  |  | 0 | 0.2197 | 0.198 | 0.2001 |
| 530 | S | 1 | 1 | 1 | 0 |  |  | 2 | 0.1591 | 0.1902 | 0.1849 |
| 531 | T | 1 | 1 | 1 | 0 |  |  | 2 | 0.1421 | 0.2033 | 0.1889 |
| 532 | N | 9 | 1 | 2 | 1 |  |  | 4 | 0.1474 | 0.2067 | 0.2148 |
| 533 | L | 1 | 1 | 1 | 0 |  |  | 4 | 0.1352 | 0.2008 | 0.21 |
| 534 | V | 2 | 1 | 1 | 0 |  |  | 4 | 0.1443 | 0.2147 | 0.2087 |
| 535 | K | 3 | 1 | 1 | 0 |  |  | 2 | 0.1587 | 0.2331 | 0.216 |
| 536 | N | 2 | 1 | 2 | 1 |  |  | 2 | 0.1803 | 0.2462 | 0.2245 |
| 537 | K | 2 | 1 | 1 | 0 |  |  | 1 | 0.1686 | 0.2369 | 0.2275 |
| 538 | C | 1 | 1 | 1 | 0 |  |  | 1 | 0.1562 | 0.2109 | 0.2029 |
| 539 | V | 1 | 1 | 1 | 0 |  |  | 1 | 0.1358 | 0.1808 | 0.175 |
| 540 | N | 1 | 1 | 1 | 0 |  |  | 2 | 0.1355 | 0.1822 | 0.15 |
| 541 | F | 1 | 1 | 1 | 0 |  |  | 2 | 0.1323 | 0.1794 | 0.1339 |
| 542 | N | 1 | 1 | 1 | 0 |  |  | 1 | 0.1309 | 0.1762 | 0.1178 |
| 543 | F | 1 | 1 | 1 | 0 |  |  | 1 | 0.1369 | 0.1925 | 0.1193 |
| 544 | N | 1 | 1 | 1 | 0 |  |  | 1 | 0.1739 | 0.221 | 0.1494 |
| 545 | G | 1 | 1 | 1 | 0 |  |  | 0 | 0.177 | 0.2504 | 0.1363 |
| 546 | L | 2 | 1 | 1 | 0 |  |  | 0 | 0.1493 | 0.2333 | 0.1347 |
| 547 | T | 3 | 2 | 2 | 0 |  |  | 0 | 0.1501 | 0.2225 | 0.113 |
| 548 | G | 1 | 1 | 1 | 0 |  |  | 0 | 0.1762 | 0.2385 | 0.1279 |
| 549 | T | 2 | 2 | 2 | 0 |  |  | 3 | 0.1565 | 0.1965 | 0.1465 |
| 550 | G | 1 | 1 | 1 | 0 |  |  | 3 | 0.1521 | 0.1914 | 0.1673 |
| 551 | V | 1 | 1 | 1 | 0 |  |  | 5 | 0.144 | 0.2001 | 0.185 |
| 552 | L | 1 | 1 | 1 | 0 |  |  | 5 | 0.141 | 0.1829 | 0.1928 |
| 553 | T | 2 | 1 | 1 | 0 |  |  | 5 | 0.1435 | 0.2119 | 0.2128 |
| 554 | E | 8 | 1 | 2 | 1 |  |  | 2 | 0.1535 | 0.2498 | 0.2154 |
| 555 | S | 1 | 1 | 1 | 0 |  |  | 2 | 0.1517 | 0.2357 | 0.2154 |
| 556 | N | 5 | 1 | 2 | 1 |  |  | 1 | 0.2291 | 0.2153 | 0.2322 |
| 557 | K | 1 | 1 | 1 | 0 |  |  | 1 | 0.2029 | 0.24 | 0.2363 |
| 558 | K | 4 | 2 | 3 | 1 |  |  | 3 | 0.2175 | 0.2735 | 0.2211 |
| 559 | F | 1 | 1 | 1 | 0 |  |  | 3 | 0.1939 | 0.2139 | 0.221 |
| 560 | L | 2 | 1 | 1 | 0 |  |  | 3 | 0.2054 | 0.2144 | 0.1984 |
| 561 | P | 2 | 2 | 2 | 0 |  |  | 2 | 0.2174 | 0.2449 | 0.1993 |
| 562 | F | 1 | 1 | 1 | 0 |  |  | 2 | 0.1896 | 0.2278 | 0.185 |
| 563 | Q | 1 | 1 | 1 | 0 |  |  | 0 | 0.133 | 0.2032 | 0.1846 |
| 564 | Q | 1 | 1 | 1 | 0 |  |  | 0 | 0.1269 | 0.2085 | 0.1789 |
| 565 | F | 1 | 1 | 1 | 0 |  |  | 0 | 0.1103 | 0.2131 | 0.1715 |
| 566 | G | 1 | 1 | 1 | 0 |  |  | 0 | 0.1143 | 0.1726 | 0.1682 |
| 567 | R | 2 | 1 | 1 | 0 |  |  | 0 | 0.1194 | 0.1643 | 0.1828 |
| 568 | D | 1 | 2 | 2 | 0 |  |  | 0 | 0.1359 | 0.1534 | 0.1886 |
| 569 | I | 6 | 2 | 2 | 0 |  |  | 0 | 0.1169 | 0.1769 | 0.1869 |
| 570 | A | 3 | 3 | 4 | 1 |  |  | 0 | 0.2223 | 0.2059 | 0.1952 |
| 571 | D | 1 | 1 | 1 | 0 |  |  | 0 | 0.191 | 0.1623 | 0.1954 |
| 572 | T | 2 | 2 | 2 | 0 |  |  | 0 | 0.2153 | 0.1508 | 0.1784 |
| 573 | T | 2 | 2 | 2 | 0 |  |  | 0 | 0.1913 | 0.16 | 0.1723 |
| 574 | D | 1 | 2 | 2 | 0 |  |  | 0 | 0.1425 | 0.1412 | 0.178 |
| 575 | A | 2 | 2 | 2 | 0 |  |  | 0 | 0.1266 | 0.1491 | 0.1789 |
| 576 | V | 1 | 1 | 1 | 0 |  |  | 3 | 0.1182 | 0.1437 | 0.1704 |
| 577 | R | 1 | 1 | 1 | 0 |  |  | 3 | 0.1351 | 0.1365 | 0.1779 |
| 578 | D | 1 | 1 | 1 | 0 |  |  | 3 | 0.1563 | 0.1486 | 0.1701 |
| 579 | P | 1 | 1 | 1 | 0 |  |  | 4 | 0.218 | 0.1771 | 0.168 |
| 580 | Q | 2 | 1 | 1 | 0 |  |  | 5 | 0.2084 | 0.1965 | 0.2001 |
| 581 | T | 1 | 1 | 1 | 0 |  |  | 4 | 0.1795 | 0.1958 | 0.2288 |
| 582 | L | 2 | 1 | 1 | 0 |  |  | 5 | 0.1685 | 0.1811 | 0.2066 |
| 583 | E | 4 | 3 | 3 | 0 |  |  | 6 | 0.1445 | 0.1821 | 0.2265 |
| 584 | I | 2 | 1 | 1 | 0 |  |  | 5 | 0.1247 | 0.1713 | 0.2119 |
| 585 | L | 1 | 1 | 1 | 0 |  |  | 4 | 0.1252 | 0.1873 | 0.1943 |
| 586 | D | 1 | 1 | 1 | 0 |  |  | 2 | 0.1256 | 0.1466 | 0.1985 |
| 587 | I | 1 | 1 | 1 | 0 |  |  | 1 | 0.1366 | 0.1432 | 0.1989 |
| 588 | T | 3 | 1 | 1 | 0 |  |  | 0 | 0.1589 | 0.1328 | 0.1913 |
| 589 | P | 1 | 1 | 1 | 0 |  |  | 0 | 0.1542 | 0.1488 | 0.1819 |
| 590 | C | 2 | 1 | 1 | 0 |  |  | 0 | 0.146 | 0.1546 | 0.1518 |
| 591 | S | 2 | 1 | 1 | 0 |  |  | 0 | 0.1521 | 0.1366 | 0.1324 |
| 592 | F | 2 | 1 | 1 | 0 |  |  | 0 | 0.1402 | 0.1213 | 0.1321 |
| 593 | G | 1 | 2 | 2 | 0 |  |  | 0 | 0.1376 | 0.1077 | 0.1493 |
| 594 | G | 1 | 2 | 2 | 0 |  |  | 0 | 0.1315 | 0.107 | 0.1375 |
| 595 | V | 1 | 1 | 1 | 0 |  |  | 0 | 0.13 | 0.0985 | 0.1478 |
| 596 | S | 1 | 2 | 2 | 0 |  |  | 0 | 0.1241 | 0.0986 | 0.1305 |
| 597 | V | 1 | 1 | 1 | 0 |  |  | 0 | 0.1226 | 0.0962 | 0.1374 |
| 598 | I | 1 | 1 | 1 | 0 |  |  | 0 | 0.1345 | 0.103 | 0.1443 |
| 599 | T | 1 | 1 | 1 | 0 |  |  | 0 | 0.155 | 0.1132 | 0.1816 |
| 600 | P | 1 | 1 | 1 | 0 |  |  | 1 | 0.1968 | 0.1467 | 0.2167 |
| 601 | G | 1 | 1 | 1 | 0 |  |  | 1 | 0.2099 | 0.1829 | 0.2515 |
| 602 | T | 1 | 1 | 1 | 0 |  |  | 2 | 0.2214 | 0.1797 | 0.2532 |
| 603 | N | 1 | 1 | 1 | 0 |  |  | 4 | 0.1878 | 0.1596 | 0.3244 |
| 604 | T | 3 | 1 | 2 | 1 |  |  | 4 | 0.2115 | 0.2036 | 0.366 |
| 605 | S | 1 | 1 | 1 | 0 |  |  | 3 | 0.1939 | 0.1671 | 0.3118 |
| 606 | N | 4 | 1 | 1 | 0 |  |  | 5 | 0.1918 | 0.1745 | 0.3446 |
| 607 | Q | 4 | 2 | 2 | 0 |  |  | 5 | 0.2038 | 0.167 | 0.2566 |
| 608 | V | 2 | 1 | 1 | 0 |  |  | 3 | 0.1619 | 0.1237 | 0.1759 |
| 609 | A | 1 | 1 | 1 | 0 |  |  | 3 | 0.153 | 0.1089 | 0.1639 |
| 610 | V | 1 | 1 | 1 | 0 |  |  | 3 | 0.1259 | 0.0971 | 0.1442 |
| 611 | L | 1 | 2 | 2 | 0 |  |  | 1 | 0.1187 | 0.0941 | 0.129 |
| 612 | Y | 1 | 1 | 1 | 0 |  |  | 0 | 0.1262 | 0.1005 | 0.14 |
| 613 | Q | 1 | 2 | 2 | 0 |  |  | 0 | 0.1296 | 0.1023 | 0.1345 |
| 614 | D | 2 | 2 | 2 | 0 |  |  | 2 | 0.1502 | 0.1366 | 0.1216 |
| 615 | V | 1 | 4 | 4 | 0 |  |  | 2 | 0.1596 | 0.1397 | 0.1374 |
| 616 | N | 1 | 1 | 1 | 0 |  |  | 2 | 0.181 | 0.1968 | 0.168 |
| 617 | C | 1 | 1 | 1 | 0 |  |  | 2 | 0.2005 | 0.1798 | 0.1815 |
| 618 | T | 1 | 1 | 1 | 0 |  |  | 3 | 0.2056 | 0.2076 | 0.2383 |
| 619 | E | 3 | 1 | 1 | 0 |  |  | 5 | 0.1815 | 0.2021 | 0.2518 |
| 620 | V | 1 | 1 | 1 | 0 |  |  | 7 | 0.1796 | 0.1899 | 0.2323 |
| 621 | P | 2 | 2 | 2 | 0 |  |  | 6 | 0.2276 | 0.1968 | 0.2228 |
| 622 | V | 6 | 2 | 3 | 1 |  |  | 7 | 0.2372 | 0.2154 | 0.2605 |
| 623 | A | 6 | 3 | 3 | 0 |  |  | 7 | 0.2014 | 0.233 | 0.2776 |
| 624 | I | 2 | 1 | 1 | 0 |  |  | 6 | 0.2159 | 0.2573 | 0.2862 |
| 625 | H | 4 | 1 | 1 | 0 |  |  | 3 | 0.3003 | 0.299 | 0.3123 |
| 626 | A | 1 | 2 | 2 | 0 |  |  | 3 | 0.3266 | 0.361 | 0.3319 |
| 627 | D | 2 | 2 | 3 | 1 |  |  | 3 | 0.2973 | 0.4147 | 0.3475 |
| 628 | Q | 2 | 1 | 1 | 0 |  |  | 3 | 0.3016 | 0.4945 | 0.3488 |
| 629 | L | 2 | 1 | 1 | 0 |  |  | 2 | 0.318 | 0.4792 | 0.2959 |
| 630 | T | 3 | 2 | 2 | 0 |  |  | 2 | 0.2406 | 0.374 | 0.2201 |
| 631 | P | 2 | 3 | 3 | 0 |  |  | 2 | 0.3086 | 0.3491 | 0.284 |
| 632 | T | 4 | 1 | 2 | 1 |  |  | 1 | 0.2674 | 0.3205 | 0.3455 |
| 633 | W | 1 | 1 | 1 | 0 |  |  | 0 | 0.2304 | 0.2808 | 0.3314 |
| 634 | R | 1 | 1 | 1 | 0 |  |  | 0 | 0.2148 | 0.2406 | 0.3283 |
| 635 | V | 2 | 1 | 1 | 0 |  |  | 0 | 0.2156 | 0.2302 | 0.2977 |
| 636 | Y | 2 | 1 | 1 | 0 |  |  | 0 | 0.1788 | 0.1808 | 0.2985 |
| 637 | S | 3 | 1 | 1 | 0 |  |  | 0 | 0.2087 | 0.2149 | 0.275 |
| 638 | T | 5 | 1 | 2 | 1 |  |  | 1 | 0.2908 | 0.3033 | 0.332 |
| 639 | G | 2 | 1 | 1 | 0 |  |  | 4 | 0.2866 | 0.3218 | 0.3667 |
| 640 | S | 10 | 1 | 2 | 1 |  |  | 5 | 0.2114 | 0.2305 | 0.2388 |
| 641 | N | 5 | 1 | 1 | 0 |  |  | 5 | 0.2089 | 0.2112 | 0.1952 |
| 642 | V | 3 | 1 | 1 | 0 |  |  | 7 | 0.1802 | 0.1754 | 0.1775 |
| 643 | F | 1 | 1 | 1 | 0 |  |  | 6 | 0.1543 | 0.1717 | 0.1594 |
| 644 | Q | 1 | 1 | 1 | 0 |  |  | 4 | 0.1572 | 0.1668 | 0.1664 |
| 645 | T | 1 | 1 | 1 | 0 |  |  | 3 | 0.1455 | 0.1637 | 0.1548 |
| 646 | R | 3 | 1 | 1 | 0 |  |  | 3 | 0.1544 | 0.2 | 0.1655 |
| 647 | A | 1 | 2 | 2 | 0 |  |  | 1 | 0.1454 | 0.147 | 0.1615 |
| 648 | G | 1 | 1 | 1 | 0 |  |  | 1 | 0.1381 | 0.1351 | 0.141 |
| 649 | C | 1 | 1 | 1 | 0 |  |  | 0 | 0.1375 | 0.1265 | 0.1438 |
| 650 | L | 1 | 1 | 1 | 0 |  |  | 1 | 0.1284 | 0.1107 | 0.1374 |
| 651 | I | 2 | 1 | 2 | 1 |  |  | 1 | 0.14 | 0.1133 | 0.1546 |
| 652 | G | 1 | 1 | 1 | 0 |  |  | 1 | 0.1567 | 0.1323 | 0.163 |
| 653 | A | 1 | 3 | 3 | 0 |  |  | 1 | 0.1645 | 0.1443 | 0.1497 |
| 654 | E | 2 | 2 | 2 | 0 |  |  | 1 | 0.168 | 0.1695 | 0.175 |
| 655 | H | 2 | 2 | 2 | 0 |  |  | 0 | 0.1568 | 0.1878 | 0.1948 |
| 656 | V | 2 | 1 | 1 | 0 |  |  | 0 | 0.1706 | 0.2147 | 0.2359 |
| 657 | N | 2 | 1 | 1 | 0 |  |  | 0 | 0.1746 | 0.2618 | 0.2493 |
| 658 | N | 5 | 1 | 1 | 0 |  |  | 0 | 0.1749 | 0.2312 | 0.2013 |
| 659 | S | 1 | 1 | 1 | 0 |  |  | 1 | 0.1739 | 0.1988 | 0.1831 |
| 660 | Y | 1 | 1 | 1 | 0 |  |  | 1 | 0.1734 | 0.176 | 0.1965 |
| 661 | E | 3 | 1 | 1 | 0 |  |  | 1 | 0.1934 | 0.1652 | 0.2171 |
| 662 | C | 1 | 1 | 1 | 0 |  |  | 1 | 0.1827 | 0.15 | 0.1841 |
| 663 | D | 1 | 1 | 1 | 0 |  |  | 1 | 0.2309 | 0.1644 | 0.1671 |
| 664 | I | 2 | 1 | 1 | 0 |  |  | 0 | 0.2032 | 0.1562 | 0.1416 |
| 665 | P | 1 | 1 | 1 | 0 |  |  | 0 | 0.1257 | 0.1426 | 0.1303 |
| 666 | I | 2 | 1 | 2 | 1 |  |  | 0 | 0.1285 | 0.132 | 0.1288 |
| 667 | G | 1 | 2 | 2 | 0 |  |  | 0 | 0.1222 | 0.1184 | 0.1417 |
| 668 | A | 1 | 1 | 1 | 0 |  |  | 0 | 0.1433 | 0.1172 | 0.1197 |
| 669 | G | 1 | 1 | 1 | 0 |  |  | 0 | 0.152 | 0.1204 | 0.114 |
| 670 | I | 1 | 1 | 1 | 0 |  |  | 0 | 0.1217 | 0.1242 | 0.1125 |
| 671 | C | 1 | 1 | 1 | 0 |  |  | 0 | 0.131 | 0.1191 | 0.1215 |
| 672 | A | 1 | 1 | 1 | 0 |  |  | 1 | 0.1616 | 0.1221 | 0.1352 |
| 673 | S | 2 | 1 | 1 | 0 |  |  | 1 | 0.2381 | 0.1423 | 0.1598 |
| 674 | Y | 1 | 1 | 1 | 0 |  |  | 1 | 0.2139 | 0.1262 | 0.1875 |
| 675 | Q | 3 | 4 | 4 | 0 |  |  | 1 | 0.2331 | 0.1572 | 0.1911 |
| 676 | T | 3 | 2 | 3 | 1 |  |  | 1 | 0.2087 | 0.15 | 0.198 |
| 677 | Q | 5 | 3 | 4 | 1 |  |  | 1 | 0.1815 | 0.1428 | 0.2191 |
| 678 | T | 3 | 1 | 2 | 1 |  |  | 1 | 0.2507 | 0.1521 | 0.2316 |
| 679 | N | 7 | 4 | 5 | 1 |  |  | 2 | 0.3177 | 0.1858 | 0.2627 |
| 680 | S | 2 | 3 | 3 | 0 |  |  | 2 | 0.2789 | 0.2395 | 0.2499 |
| 681 | P | 2 | 4 | 4 | 0 |  |  | 3 | 0.2568 | 0.2824 | 0.2662 |
| 682 | R | 2 | 3 | 3 | 0 |  |  | 3 | 0.319 | 0.2694 | 0.2638 |
| 683 | R | 2 | 4 | 4 | 0 |  |  | 4 | 0.2946 | 0.2037 | 0.2237 |
| 684 | A | 4 | 3 | 5 | 2 |  |  | 3 | 0.2557 | 0.166 | 0.2191 |
| 685 | R | 3 | 2 | 2 | 0 |  |  | 4 | 0.2023 | 0.1413 | 0.2195 |
| 686 | S | 4 | 3 | 3 | 0 |  |  | 3 | 0.1862 | 0.1455 | 0.2097 |
| 687 | V | 4 | 2 | 2 | 0 |  |  | 2 | 0.2413 | 0.1554 | 0.193 |
| 688 | A | 5 | 2 | 3 | 1 |  |  | 1 | 0.2786 | 0.2129 | 0.1843 |
| 689 | S | 3 | 2 | 3 | 1 |  |  | 1 | 0.3219 | 0.2642 | 0.2033 |
| 690 | Q | 4 | 1 | 2 | 1 |  |  | 0 | 0.3617 | 0.3317 | 0.225 |
| 691 | S | 2 | 1 | 1 | 0 |  |  | 1 | 0.2489 | 0.2452 | 0.191 |
| 692 | I | 1 | 1 | 1 | 0 |  |  | 1 | 0.1877 | 0.1678 | 0.1599 |
| 693 | I | 3 | 1 | 1 | 0 |  |  | 1 | 0.1636 | 0.1632 | 0.1508 |
| 694 | A | 2 | 1 | 1 | 0 |  |  | 1 | 0.1453 | 0.147 | 0.1287 |
| 695 | Y | 1 | 1 | 1 | 0 |  |  | 1 | 0.1391 | 0.1533 | 0.1299 |
| 696 | T | 1 | 1 | 1 | 0 |  |  | 0 | 0.1433 | 0.1617 | 0.1295 |
| 697 | M | 1 | 1 | 1 | 0 |  |  | 0 | 0.1639 | 0.1661 | 0.1383 |
| 698 | S | 1 | 1 | 1 | 0 |  |  | 0 | 0.1808 | 0.1623 | 0.1774 |
| 699 | L | 1 | 1 | 1 | 0 |  |  | 1 | 0.1958 | 0.1665 | 0.1606 |
| 700 | G | 1 | 1 | 1 | 0 |  |  | 2 | 0.2316 | 0.194 | 0.1544 |
| 701 | A | 2 | 1 | 1 | 0 |  |  | 3 | 0.2226 | 0.2017 | 0.1829 |
| 702 | E | 3 | 2 | 2 | 0 |  |  | 3 | 0.2285 | 0.1959 | 0.1896 |
| 703 | N | 3 | 2 | 2 | 0 |  |  | 3 | 0.169 | 0.1781 | 0.2207 |
| 704 | S | 2 | 2 | 2 | 0 |  |  | 2 | 0.1631 | 0.1968 | 0.2651 |
| 705 | V | 2 | 2 | 2 | 0 |  |  | 2 | 0.1566 | 0.1933 | 0.2547 |
| 706 | A | 2 | 2 | 2 | 0 |  |  | 1 | 0.2101 | 0.2403 | 0.2173 |
| 707 | Y | 1 | 1 | 1 | 0 |  |  | 1 | 0.1501 | 0.21 | 0.167 |
| 708 | S | 2 | 1 | 1 | 0 |  |  | 1 | 0.14 | 0.1963 | 0.1778 |
| 709 | N | 1 | 1 | 1 | 0 |  |  | 1 | 0.1352 | 0.1971 | 0.1578 |
| 710 | N | 1 | 1 | 1 | 0 |  |  | 1 | 0.1394 | 0.1631 | 0.2005 |
| 711 | S | 2 | 1 | 1 | 0 |  |  | 0 | 0.1342 | 0.1576 | 0.1852 |
| 712 | I | 1 | 1 | 1 | 0 |  |  | 0 | 0.1203 | 0.1419 | 0.193 |
| 713 | A | 1 | 1 | 1 | 0 |  |  | 0 | 0.1236 | 0.1394 | 0.1829 |
| 714 | I | 1 | 2 | 2 | 0 |  |  | 0 | 0.1271 | 0.122 | 0.1755 |
| 715 | P | 1 | 1 | 1 | 0 |  |  | 1 | 0.1324 | 0.1182 | 0.1682 |
| 716 | T | 2 | 2 | 2 | 0 |  |  | 2 | 0.145 | 0.1248 | 0.18 |
| 717 | N | 1 | 1 | 1 | 0 |  |  | 2 | 0.1504 | 0.1179 | 0.1701 |
| 718 | F | 1 | 1 | 1 | 0 |  |  | 2 | 0.1393 | 0.1057 | 0.1514 |
| 719 | T | 2 | 2 | 2 | 0 |  |  | 2 | 0.1432 | 0.1078 | 0.1341 |
| 720 | I | 1 | 2 | 2 | 0 |  |  | 1 | 0.1225 | 0.0981 | 0.1289 |
| 721 | S | 2 | 1 | 1 | 0 |  |  | 0 | 0.1268 | 0.1131 | 0.1232 |
| 722 | V | 2 | 1 | 1 | 0 |  |  | 0 | 0.1175 | 0.1234 | 0.1215 |
| 723 | T | 2 | 1 | 1 | 0 |  |  | 0 | 0.1056 | 0.1228 | 0.133 |
| 724 | T | 2 | 1 | 1 | 0 |  |  | 0 | 0.0971 | 0.1107 | 0.131 |
| 725 | E | 1 | 1 | 1 | 0 |  |  | 0 | 0.0867 | 0.104 | 0.1161 |
| 726 | I | 2 | 1 | 1 | 0 |  |  | 0 | 0.0904 | 0.113 | 0.1158 |
| 727 | L | 2 | 1 | 1 | 0 |  |  | 0 | 0.0889 | 0.1044 | 0.1045 |
| 728 | P | 1 | 1 | 1 | 0 |  |  | 0 | 0.0922 | 0.1136 | 0.1088 |
| 729 | V | 1 | 1 | 1 | 0 |  |  | 0 | 0.0976 | 0.1087 | 0.1038 |
| 730 | S | 1 | 1 | 1 | 0 |  |  | 0 | 0.1014 | 0.1183 | 0.1212 |
| 731 | M | 2 | 2 | 2 | 0 |  |  | 0 | 0.1205 | 0.1249 | 0.1205 |
| 732 | T | 3 | 1 | 1 | 0 |  |  | 0 | 0.1117 | 0.129 | 0.1122 |
| 733 | K | 1 | 1 | 1 | 0 |  |  | 0 | 0.1133 | 0.1157 | 0.1075 |
| 734 | T | 1 | 1 | 1 | 0 |  |  | 0 | 0.1072 | 0.1056 | 0.1144 |
| 735 | S | 2 | 2 | 2 | 0 |  |  | 0 | 0.1234 | 0.097 | 0.1287 |
| 736 | V | 1 | 1 | 1 | 0 |  |  | 0 | 0.1266 | 0.1023 | 0.1087 |
| 737 | D | 1 | 1 | 1 | 0 |  |  | 0 | 0.1256 | 0.123 | 0.1037 |
| 738 | C | 1 | 1 | 1 | 0 |  |  | 0 | 0.1212 | 0.1212 | 0.1077 |
| 739 | T | 2 | 1 | 1 | 0 |  |  | 0 | 0.1205 | 0.1321 | 0.1063 |
| 740 | M | 1 | 2 | 2 | 0 |  |  | 0 | 0.1207 | 0.13 | 0.0974 |
| 741 | Y | 1 | 1 | 1 | 0 |  |  | 0 | 0.1164 | 0.1234 | 0.0909 |
| 742 | I | 1 | 1 | 1 | 0 |  |  | 0 | 0.1155 | 0.1309 | 0.0942 |
| 743 | C | 1 | 1 | 1 | 0 |  |  | 0 | 0.1172 | 0.1379 | 0.0925 |
| 744 | G | 1 | 1 | 1 | 0 |  |  | 0 | 0.1329 | 0.1543 | 0.1224 |
| 745 | D | 1 | 2 | 2 | 0 |  |  | 0 | 0.1551 | 0.1758 | 0.1302 |
| 746 | S | 1 | 1 | 1 | 0 |  |  | 0 | 0.1335 | 0.1931 | 0.1206 |
| 747 | T | 5 | 2 | 3 | 1 |  |  | 0 | 0.1518 | 0.2019 | 0.1377 |
| 748 | E | 1 | 1 | 1 | 0 |  |  | 0 | 0.1153 | 0.2036 | 0.1389 |
| 749 | C | 1 | 1 | 1 | 0 |  |  | 0 | 0.1053 | 0.1795 | 0.1202 |
| 750 | S | 2 | 1 | 1 | 0 |  |  | 0 | 0.1204 | 0.1803 | 0.126 |
| 751 | N | 1 | 1 | 1 | 0 |  |  | 0 | 0.1162 | 0.1925 | 0.146 |
| 752 | L | 1 | 2 | 2 | 0 |  |  | 0 | 0.0968 | 0.189 | 0.1531 |
| 753 | L | 1 | 1 | 1 | 0 |  |  | 0 | 0.1069 | 0.1835 | 0.1549 |
| 754 | L | 1 | 1 | 1 | 0 |  |  | 0 | 0.1176 | 0.191 | 0.1776 |
| 755 | Q | 1 | 1 | 1 | 0 |  |  | 0 | 0.1056 | 0.1976 | 0.1942 |
| 756 | Y | 1 | 1 | 1 | 0 |  |  | 0 | 0.099 | 0.1932 | 0.2322 |
| 757 | G | 1 | 1 | 1 | 0 |  |  | 0 | 0.1184 | 0.2043 | 0.2756 |
| 758 | S | 1 | 1 | 1 | 0 |  |  | 0 | 0.1124 | 0.171 | 0.1887 |
| 759 | F | 1 | 1 | 1 | 0 |  |  | 0 | 0.1066 | 0.1606 | 0.1678 |
| 760 | C | 1 | 1 | 1 | 0 |  |  | 0 | 0.1125 | 0.1699 | 0.153 |
| 761 | T | 2 | 1 | 1 | 0 |  |  | 0 | 0.1267 | 0.1525 | 0.1338 |
| 762 | Q | 1 | 1 | 1 | 0 |  |  | 0 | 0.1215 | 0.1232 | 0.1341 |
| 763 | L | 1 | 1 | 1 | 0 |  |  | 0 | 0.1033 | 0.1091 | 0.1242 |
| 764 | N | 1 | 1 | 1 | 0 |  |  | 0 | 0.1119 | 0.1182 | 0.141 |
| 765 | R | 1 | 4 | 4 | 0 |  |  | 0 | 0.1268 | 0.1095 | 0.1352 |
| 766 | A | 1 | 1 | 1 | 0 |  |  | 0 | 0.1167 | 0.0929 | 0.1176 |
| 767 | L | 1 | 1 | 1 | 0 |  |  | 0 | 0.1092 | 0.0942 | 0.1158 |
| 768 | T | 3 | 2 | 2 | 0 |  |  | 0 | 0.1153 | 0.0944 | 0.1271 |
| 769 | G | 1 | 2 | 2 | 0 |  |  | 0 | 0.127 | 0.1004 | 0.1223 |
| 770 | I | 2 | 1 | 1 | 0 |  |  | 0 | 0.1297 | 0.1048 | 0.1125 |
| 771 | A | 1 | 2 | 2 | 0 |  |  | 0 | 0.1236 | 0.0972 | 0.104 |
| 772 | V | 4 | 3 | 3 | 0 |  |  | 0 | 0.1365 | 0.0954 | 0.1154 |
| 773 | E | 1 | 1 | 1 | 0 |  |  | 0 | 0.1309 | 0.1089 | 0.129 |
| 774 | Q | 1 | 1 | 1 | 0 |  |  | 0 | 0.1179 | 0.1244 | 0.1134 |
| 775 | D | 1 | 1 | 1 | 0 |  |  | 0 | 0.1206 | 0.1233 | 0.119 |
| 776 | K | 3 | 1 | 1 | 0 |  |  | 0 | 0.1218 | 0.1145 | 0.1437 |
| 777 | N | 1 | 1 | 1 | 0 |  |  | 0 | 0.1135 | 0.1286 | 0.1197 |
| 778 | T | 1 | 1 | 1 | 0 |  |  | 0 | 0.1018 | 0.1338 | 0.1378 |
| 779 | Q | 2 | 2 | 2 | 0 |  |  | 0 | 0.1025 | 0.12 | 0.1437 |
| 780 | E | 2 | 2 | 2 | 0 |  |  | 0 | 0.1011 | 0.1294 | 0.1235 |
| 781 | V | 1 | 1 | 1 | 0 |  |  | 0 | 0.0958 | 0.145 | 0.1133 |
| 782 | F | 1 | 1 | 1 | 0 |  |  | 0 | 0.1044 | 0.1535 | 0.1395 |
| 783 | A | 2 | 1 | 1 | 0 |  |  | 1 | 0.1148 | 0.2188 | 0.2203 |
| 784 | Q | 1 | 1 | 1 | 0 |  |  | 1 | 0.1128 | 0.1898 | 0.1426 |
| 785 | V | 2 | 1 | 1 | 0 |  |  | 1 | 0.1109 | 0.1503 | 0.1396 |
| 786 | K | 1 | 1 | 1 | 0 |  |  | 1 | 0.1397 | 0.1772 | 0.1546 |
| 787 | Q | 2 | 2 | 2 | 0 |  |  | 1 | 0.1367 | 0.1575 | 0.1611 |
| 788 | I | 2 | 1 | 1 | 0 |  |  | 0 | 0.1348 | 0.1622 | 0.158 |
| 789 | Y | 1 | 1 | 1 | 0 |  |  | 0 | 0.1277 | 0.1516 | 0.1509 |
| 790 | K | 1 | 1 | 1 | 0 |  |  | 0 | 0.1514 | 0.1924 | 0.1656 |
| 791 | T | 1 | 2 | 2 | 0 |  |  | 0 | 0.1585 | 0.1888 | 0.1648 |
| 792 | P | 1 | 1 | 1 | 0 |  |  | 1 | 0.1673 | 0.2173 | 0.2086 |
| 793 | P | 4 | 1 | 1 | 0 |  |  | 2 | 0.1842 | 0.2246 | 0.2561 |
| 794 | I | 2 | 1 | 1 | 0 |  |  | 3 | 0.1782 | 0.1896 | 0.2431 |
| 795 | K | 2 | 1 | 1 | 0 |  |  | 3 | 0.1728 | 0.1603 | 0.3084 |
| 796 | D | 2 | 1 | 1 | 0 |  |  | 3 | 0.1767 | 0.1542 | 0.3349 |
| 797 | F | 1 | 2 | 2 | 0 |  |  | 3 | 0.1809 | 0.1684 | 0.3399 |
| 798 | G | 2 | 1 | 1 | 0 |  |  | 3 | 0.2735 | 0.2422 | 0.4105 |
| 799 | G | 1 | 1 | 1 | 0 |  |  | 2 | 0.2524 | 0.1901 | 0.3342 |
| 800 | F | 1 | 1 | 1 | 0 |  |  | 2 | 0.1699 | 0.125 | 0.2674 |
| 801 | N | 1 | 1 | 1 | 0 |  |  | 2 | 0.1461 | 0.131 | 0.2536 |
| 802 | F | 1 | 1 | 1 | 0 |  |  | 3 | 0.1375 | 0.1464 | 0.2049 |
| 803 | S | 2 | 1 | 2 | 1 |  |  | 2 | 0.159 | 0.16 | 0.2388 |
| 804 | Q | 1 | 2 | 2 | 0 |  |  | 2 | 0.1571 | 0.1391 | 0.2192 |
| 805 | I | 2 | 2 | 2 | 0 |  |  | 3 | 0.1337 | 0.1153 | 0.1694 |
| 806 | L | 1 | 1 | 1 | 0 |  |  | 6 | 0.1378 | 0.1196 | 0.1633 |
| 807 | P | 1 | 1 | 1 | 0 |  |  | 5 | 0.1666 | 0.1574 | 0.1596 |
| 808 | D | 1 | 2 | 2 | 0 |  |  | 6 | 0.2097 | 0.2093 | 0.1659 |
| 809 | P | 2 | 2 | 2 | 0 |  |  | 6 | 0.2661 | 0.2948 | 0.1999 |
| 810 | S | 3 | 1 | 1 | 0 |  |  | 5 | 0.3077 | 0.3641 | 0.2419 |
| 811 | K | 1 | 1 | 1 | 0 |  |  | 2 | 0.3038 | 0.56 | 0.3016 |
| 812 | P | 2 | 2 | 2 | 0 |  |  | 1 | 0.2664 | 0.4658 | 0.2646 |
| 813 | S | 3 | 1 | 1 | 0 |  |  | 2 | 0.3033 | 0.3575 | 0.2491 |
| 814 | K | 2 | 1 | 1 | 0 |  |  | 2 | 0.243 | 0.2307 | 0.1804 |
| 815 | R | 1 | 1 | 1 | 0 |  |  | 2 | 0.1598 | 0.1556 | 0.1564 |
| 816 | S | 1 | 1 | 1 | 0 |  |  | 2 | 0.1538 | 0.1296 | 0.1604 |
| 817 | F | 1 | 2 | 2 | 0 |  |  | 4 | 0.1561 | 0.1227 | 0.1889 |
| 818 | I | 1 | 2 | 2 | 0 |  |  | 4 | 0.1244 | 0.1228 | 0.1327 |
| 819 | E | 1 | 1 | 1 | 0 |  |  | 5 | 0.1184 | 0.1282 | 0.1336 |
| 820 | D | 1 | 1 | 1 | 0 |  |  | 5 | 0.1474 | 0.127 | 0.144 |
| 821 | L | 1 | 2 | 2 | 0 |  |  | 7 | 0.1445 | 0.1275 | 0.1304 |
| 822 | L | 1 | 2 | 2 | 0 |  |  | 6 | 0.1258 | 0.1329 | 0.1251 |
| 823 | F | 2 | 1 | 1 | 0 |  |  | 5 | 0.1374 | 0.1363 | 0.1343 |
| 824 | N | 1 | 1 | 1 | 0 |  |  | 3 | 0.1677 | 0.1489 | 0.1381 |
| 825 | K | 1 | 1 | 1 | 0 |  |  | 3 | 0.1639 | 0.1529 | 0.1277 |
| 826 | V | 1 | 2 | 2 | 0 |  |  | 2 | 0.1575 | 0.1457 | 0.1266 |
| 827 | T | 1 | 1 | 1 | 0 |  |  | 1 | 0.1913 | 0.1545 | 0.1372 |
| 828 | L | 1 | 1 | 1 | 0 |  |  | 1 | 0.2148 | 0.1802 | 0.1397 |
| 829 | A | 2 | 2 | 2 | 0 |  |  | 1 | 0.2098 | 0.1568 | 0.129 |
| 830 | D | 1 | 2 | 2 | 0 |  |  | 1 | 0.2282 | 0.1243 | 0.1257 |
| 831 | A | 2 | 3 | 3 | 0 |  |  | 1 | 0.2676 | 0.1462 | 0.1399 |
| 832 | G | 1 | 2 | 2 | 0 |  |  | 1 | 0.3608 | 0.1369 | 0.1369 |
| 833 | F | 1 | 1 | 1 | 0 |  |  | 0 | 0.5047 | 0.1245 | 0.1344 |
| 834 | I | 2 | 2 | 2 | 0 |  |  | 0 | 0.4595 | 0.1421 | 0.1491 |
| 835 | K | 1 | 1 | 1 | 0 |  |  | 0 | 0.6051 | 0.1595 | 0.1558 |
| 836 | Q | 1 | 1 | 1 | 0 |  |  | 0 | 0.6556 | 0.1486 | 0.1537 |
| 837 | Y | 1 | 1 | 1 | 0 |  |  | 0 | 0.6432 | 0.128 | 0.1541 |
| 838 | G | 2 | 1 | 1 | 0 |  |  | 0 | 0.7203 | 0.1309 | 0.1468 |
| 839 | D | 3 | 5 | 5 | 0 |  |  | 0 | 0.7125 | 0.1189 | 0.1195 |
| 840 | C | 1 | 1 | 1 | 0 |  |  | 0 | 0.6558 | 0.1385 | 0.1538 |
| 841 | L | 1 | 1 | 1 | 0 |  |  | 0 | 0.6452 | 0.219 | 0.1657 |
| 842 | G | 1 | 2 | 2 | 0 |  |  | 1 | 0.5605 | 0.3337 | 0.2268 |
| 843 | D | 2 | 1 | 1 | 0 |  |  | 3 | 0.4284 | 0.3834 | 0.2519 |
| 844 | I | 2 | 1 | 1 | 0 |  |  | 3 | 0.2863 | 0.4682 | 0.2682 |
| 845 | A | 3 | 3 | 3 | 0 |  |  | 3 | 0.2463 | 0.4085 | 0.3766 |
| 846 | A | 1 | 2 | 2 | 0 |  |  | 4 | 0.1869 | 0.3533 | 0.4304 |
| 847 | R | 1 | 1 | 1 | 0 |  |  | 3 | 0.1739 | 0.2639 | 0.3816 |
| 848 | D | 1 | 1 | 1 | 0 |  |  | 2 | 0.1677 | 0.1926 | 0.3885 |
| 849 | L | 1 | 2 | 2 | 0 |  |  | 1 | 0.1917 | 0.1605 | 0.1835 |
| 850 | I | 1 | 1 | 1 | 0 |  |  | 1 | 0.1784 | 0.1619 | 0.2093 |
| 851 | C | 1 | 1 | 1 | 0 |  |  | 0 | 0.2028 | 0.1532 | 0.1896 |
| 852 | A | 1 | 2 | 2 | 0 |  |  | 0 | 0.1711 | 0.121 | 0.1749 |
| 853 | Q | 1 | 1 | 1 | 0 |  |  | 0 | 0.1629 | 0.1149 | 0.1345 |
| 854 | K | 1 | 1 | 1 | 0 |  |  | 0 | 0.1405 | 0.1162 | 0.1838 |
| 855 | F | 1 | 1 | 1 | 0 |  |  | 0 | 0.1595 | 0.1114 | 0.1564 |
| 856 | N | 1 | 2 | 2 | 0 |  |  | 0 | 0.1789 | 0.1105 | 0.1613 |
| 857 | G | 2 | 1 | 1 | 0 |  |  | 0 | 0.1637 | 0.1275 | 0.1173 |
| 858 | L | 1 | 1 | 1 | 0 |  |  | 0 | 0.125 | 0.1137 | 0.0974 |
| 859 | T | 1 | 2 | 2 | 0 |  |  | 0 | 0.1178 | 0.1103 | 0.0994 |
| 860 | V | 1 | 3 | 3 | 0 |  |  | 0 | 0.1376 | 0.1083 | 0.1109 |
| 861 | L | 1 | 2 | 2 | 0 |  |  | 0 | 0.1674 | 0.106 | 0.1058 |
| 862 | P | 2 | 1 | 1 | 0 |  |  | 0 | 0.1722 | 0.1069 | 0.1126 |
| 863 | P | 1 | 1 | 1 | 0 |  |  | 0 | 0.1704 | 0.1134 | 0.121 |
| 864 | L | 1 | 1 | 1 | 0 |  |  | 0 | 0.117 | 0.1085 | 0.1244 |
| 865 | L | 1 | 1 | 1 | 0 |  |  | 0 | 0.1089 | 0.1472 | 0.1487 |
| 866 | T | 1 | 1 | 1 | 0 |  |  | 1 | 0.1093 | 0.1916 | 0.131 |
| 867 | D | 2 | 2 | 2 | 0 |  |  | 2 | 0.1076 | 0.2354 | 0.1319 |
| 868 | E | 2 | 2 | 2 | 0 |  |  | 2 | 0.1114 | 0.233 | 0.1378 |
| 869 | M | 1 | 1 | 1 | 0 |  |  | 2 | 0.1062 | 0.1881 | 0.1261 |
| 870 | I | 2 | 2 | 2 | 0 |  |  | 2 | 0.0982 | 0.1785 | 0.1152 |
| 871 | A | 1 | 1 | 1 | 0 |  |  | 1 | 0.1028 | 0.1964 | 0.1238 |
| 872 | Q | 2 | 1 | 1 | 0 |  |  | 1 | 0.1053 | 0.1825 | 0.1267 |
| 873 | Y | 1 | 1 | 1 | 0 |  |  | 1 | 0.0972 | 0.1463 | 0.1123 |
| 874 | T | 1 | 1 | 1 | 0 |  |  | 1 | 0.0951 | 0.1419 | 0.1096 |
| 875 | S | 2 | 1 | 1 | 0 |  |  | 1 | 0.105 | 0.1396 | 0.1255 |
| 876 | A | 1 | 1 | 1 | 0 |  |  | 1 | 0.1017 | 0.1279 | 0.1295 |
| 877 | L | 1 | 1 | 1 | 0 |  |  | 0 | 0.096 | 0.1081 | 0.1202 |
| 878 | L | 3 | 1 | 1 | 0 |  |  | 0 | 0.103 | 0.1068 | 0.1232 |
| 879 | A | 2 | 4 | 4 | 0 |  |  | 0 | 0.1111 | 0.1093 | 0.1449 |
| 880 | G | 1 | 1 | 1 | 0 |  |  | 0 | 0.1102 | 0.106 | 0.1546 |
| 881 | T | 2 | 1 | 1 | 0 |  |  | 0 | 0.1116 | 0.1003 | 0.146 |
| 882 | I | 3 | 1 | 1 | 0 |  |  | 0 | 0.1338 | 0.1041 | 0.1409 |
| 883 | T | 1 | 1 | 1 | 0 |  |  | 0 | 0.1399 | 0.1051 | 0.146 |
| 884 | S | 2 | 2 | 2 | 0 |  |  | 0 | 0.1486 | 0.104 | 0.1589 |
| 885 | G | 1 | 1 | 1 | 0 |  |  | 0 | 0.201 | 0.1152 | 0.1774 |
| 886 | W | 2 | 1 | 1 | 0 |  |  | 0 | 0.1868 | 0.1176 | 0.126 |
| 887 | T | 2 | 1 | 1 | 0 |  |  | 0 | 0.1572 | 0.1125 | 0.1176 |
| 888 | F | 1 | 2 | 2 | 0 |  |  | 0 | 0.2027 | 0.1195 | 0.1686 |
| 889 | G | 1 | 2 | 2 | 0 |  |  | 1 | 0.1568 | 0.1371 | 0.1923 |
| 890 | A | 1 | 1 | 1 | 0 |  |  | 3 | 0.1862 | 0.1321 | 0.1753 |
| 891 | G | 1 | 1 | 1 | 0 |  |  | 3 | 0.189 | 0.1477 | 0.1841 |
| 892 | A | 2 | 3 | 3 | 0 |  |  | 4 | 0.1764 | 0.1491 | 0.1736 |
| 893 | A | 1 | 2 | 2 | 0 |  |  | 4 | 0.1977 | 0.131 | 0.1923 |
| 894 | L | 1 | 1 | 1 | 0 |  |  | 3 | 0.1753 | 0.1263 | 0.1751 |
| 895 | Q | 1 | 1 | 1 | 0 |  |  | 1 | 0.1692 | 0.1155 | 0.1509 |
| 896 | I | 1 | 1 | 1 | 0 |  |  | 1 | 0.1493 | 0.1005 | 0.1476 |
| 897 | P | 1 | 1 | 1 | 0 |  |  | 0 | 0.1457 | 0.1101 | 0.1542 |
| 898 | F | 1 | 1 | 1 | 0 |  |  | 0 | 0.1542 | 0.1191 | 0.1538 |
| 899 | A | 1 | 1 | 1 | 0 |  |  | 0 | 0.1378 | 0.1388 | 0.1558 |
| 900 | M | 1 | 1 | 1 | 0 |  |  | 0 | 0.1173 | 0.1279 | 0.1445 |
| 901 | Q | 1 | 1 | 1 | 0 |  |  | 0 | 0.1252 | 0.1012 | 0.1343 |
| 902 | M | 1 | 1 | 1 | 0 |  |  | 0 | 0.1157 | 0.1075 | 0.1381 |
| 903 | A | 1 | 1 | 1 | 0 |  |  | 0 | 0.1123 | 0.1396 | 0.136 |
| 904 | Y | 1 | 1 | 1 | 0 |  |  | 0 | 0.1191 | 0.1395 | 0.1231 |
| 905 | R | 1 | 1 | 1 | 0 |  |  | 0 | 0.1186 | 0.108 | 0.1174 |
| 906 | F | 1 | 1 | 1 | 0 |  |  | 0 | 0.1128 | 0.1207 | 0.1248 |
| 907 | N | 1 | 1 | 1 | 0 |  |  | 0 | 0.1265 | 0.1693 | 0.124 |
| 908 | G | 1 | 1 | 1 | 0 |  |  | 0 | 0.1372 | 0.213 | 0.1183 |
| 909 | I | 1 | 1 | 1 | 0 |  |  | 0 | 0.1363 | 0.1436 | 0.1226 |
| 910 | G | 1 | 1 | 1 | 0 |  |  | 0 | 0.1488 | 0.1814 | 0.149 |
| 911 | V | 1 | 1 | 1 | 0 |  |  | 0 | 0.1533 | 0.1392 | 0.1318 |
| 912 | T | 1 | 1 | 1 | 0 |  |  | 0 | 0.1871 | 0.1357 | 0.1461 |
| 913 | Q | 1 | 1 | 1 | 0 |  |  | 0 | 0.1585 | 0.1444 | 0.1469 |
| 914 | N | 1 | 1 | 1 | 0 |  |  | 2 | 0.1685 | 0.151 | 0.1605 |
| 915 | V | 1 | 1 | 1 | 0 |  |  | 2 | 0.1944 | 0.138 | 0.1445 |
| 916 | L | 1 | 1 | 1 | 0 |  |  | 4 | 0.1741 | 0.1412 | 0.1369 |
| 917 | Y | 1 | 2 | 2 | 0 |  |  | 4 | 0.158 | 0.1508 | 0.1619 |
| 918 | E | 2 | 1 | 1 | 0 |  |  | 5 | 0.1649 | 0.1626 | 0.1624 |
| 919 | N | 1 | 1 | 1 | 0 |  |  | 3 | 0.16 | 0.1749 | 0.151 |
| 920 | Q | 1 | 1 | 1 | 0 |  |  | 5 | 0.1538 | 0.145 | 0.1468 |
| 921 | K | 1 | 1 | 1 | 0 |  |  | 4 | 0.1664 | 0.1426 | 0.1531 |
| 922 | L | 2 | 1 | 1 | 0 |  |  | 4 | 0.1591 | 0.1446 | 0.146 |
| 923 | I | 1 | 1 | 1 | 0 |  |  | 3 | 0.1385 | 0.1315 | 0.1358 |
| 924 | A | 1 | 2 | 2 | 0 |  |  | 3 | 0.1379 | 0.1242 | 0.1416 |
| 925 | N | 1 | 1 | 1 | 0 |  |  | 3 | 0.152 | 0.1332 | 0.1444 |
| 926 | Q | 1 | 1 | 1 | 0 |  |  | 1 | 0.1407 | 0.1316 | 0.1347 |
| 927 | F | 1 | 1 | 1 | 0 |  |  | 2 | 0.1377 | 0.122 | 0.134 |
| 928 | N | 1 | 1 | 1 | 0 |  |  | 2 | 0.1445 | 0.1294 | 0.1403 |
| 929 | S | 2 | 2 | 2 | 0 |  |  | 2 | 0.1498 | 0.1383 | 0.136 |
| 930 | A | 1 | 2 | 2 | 0 |  |  | 1 | 0.1415 | 0.1301 | 0.1288 |
| 931 | I | 1 | 1 | 1 | 0 |  |  | 3 | 0.1348 | 0.1244 | 0.1299 |
| 932 | G | 3 | 2 | 2 | 0 |  |  | 3 | 0.1463 | 0.1366 | 0.1367 |
| 933 | K | 2 | 1 | 1 | 0 |  |  | 3 | 0.1504 | 0.1376 | 0.1349 |
| 934 | I | 1 | 2 | 2 | 0 |  |  | 3 | 0.1346 | 0.1244 | 0.1303 |
| 935 | Q | 1 | 2 | 2 | 0 |  |  | 4 | 0.137 | 0.1261 | 0.1322 |
| 936 | D | 2 | 3 | 3 | 0 |  |  | 5 | 0.1519 | 0.1358 | 0.1415 |
| 937 | S | 1 | 1 | 1 | 0 |  |  | 7 | 0.153 | 0.1305 | 0.1455 |
| 938 | L | 2 | 2 | 2 | 0 |  |  | 10 | 0.1466 | 0.1276 | 0.1436 |
| 939 | S | 2 | 2 | 2 | 0 |  |  | 12 | 0.1637 | 0.1432 | 0.1535 |
| 940 | S | 2 | 3 | 3 | 0 |  |  | 11 | 0.176 | 0.1529 | 0.1573 |
| 941 | T | 1 | 2 | 2 | 0 |  |  | 8 | 0.1957 | 0.1608 | 0.1646 |
| 942 | A | 3 | 1 | 1 | 0 |  |  | 8 | 0.218 | 0.1747 | 0.1956 |
| 943 | S | 2 | 3 | 3 | 0 |  |  | 3 | 0.2024 | 0.189 | 0.1635 |
| 944 | A | 1 | 1 | 1 | 0 |  |  | 0 | 0.2514 | 0.2561 | 0.1564 |
| 945 | L | 1 | 1 | 1 | 0 |  |  | 0 | 0.1434 | 0.1306 | 0.1276 |
| 946 | G | 1 | 1 | 1 | 0 |  |  | 0 | 0.1594 | 0.1804 | 0.1541 |
| 947 | K | 1 | 1 | 1 | 0 |  |  | 0 | 0.1298 | 0.1238 | 0.1259 |
| 948 | L | 1 | 2 | 2 | 0 |  |  | 0 | 0.1177 | 0.113 | 0.1157 |
| 949 | Q | 1 | 1 | 1 | 0 |  |  | 0 | 0.1164 | 0.117 | 0.1153 |
| 950 | D | 1 | 2 | 2 | 0 |  |  | 0 | 0.1081 | 0.1147 | 0.1307 |
| 951 | V | 1 | 1 | 1 | 0 |  |  | 0 | 0.118 | 0.1148 | 0.1439 |
| 952 | V | 2 | 2 | 2 | 0 |  |  | 0 | 0.1135 | 0.1192 | 0.154 |
| 953 | N | 1 | 1 | 1 | 0 |  |  | 0 | 0.1097 | 0.1108 | 0.1306 |
| 954 | Q | 2 | 2 | 2 | 0 |  |  | 0 | 0.1114 | 0.1096 | 0.1089 |
| 955 | N | 1 | 1 | 1 | 0 |  |  | 0 | 0.1083 | 0.1105 | 0.0979 |
| 956 | A | 1 | 1 | 1 | 0 |  |  | 0 | 0.1262 | 0.1035 | 0.1009 |
| 957 | Q | 2 | 1 | 1 | 0 |  |  | 0 | 0.1345 | 0.1034 | 0.1025 |
| 958 | A | 1 | 1 | 1 | 0 |  |  | 0 | 0.1216 | 0.1078 | 0.0925 |
| 959 | L | 1 | 1 | 1 | 0 |  |  | 0 | 0.1258 | 0.1033 | 0.0886 |
| 960 | N | 1 | 2 | 2 | 0 |  |  | 0 | 0.1477 | 0.1051 | 0.0941 |
| 961 | T | 1 | 1 | 1 | 0 |  |  | 0 | 0.1423 | 0.1196 | 0.1008 |
| 962 | L | 1 | 1 | 1 | 0 |  |  | 0 | 0.1229 | 0.1148 | 0.0996 |
| 963 | V | 1 | 2 | 2 | 0 |  |  | 0 | 0.1361 | 0.1072 | 0.0992 |
| 964 | K | 1 | 1 | 1 | 0 |  |  | 0 | 0.1497 | 0.123 | 0.1085 |
| 965 | Q | 1 | 1 | 1 | 0 |  |  | 0 | 0.1333 | 0.1291 | 0.1226 |
| 966 | L | 1 | 1 | 1 | 0 |  |  | 0 | 0.1358 | 0.1469 | 0.124 |
| 967 | S | 1 | 1 | 1 | 0 |  |  | 0 | 0.142 | 0.166 | 0.1349 |
| 968 | S | 2 | 1 | 1 | 0 |  |  | 0 | 0.1299 | 0.1443 | 0.1663 |
| 969 | N | 1 | 1 | 1 | 0 |  |  | 0 | 0.1283 | 0.1471 | 0.1277 |
| 970 | F | 1 | 2 | 2 | 0 |  |  | 0 | 0.1283 | 0.1409 | 0.1311 |
| 971 | G | 1 | 1 | 1 | 0 |  |  | 0 | 0.1461 | 0.1524 | 0.1611 |
| 972 | A | 1 | 1 | 1 | 0 |  |  | 0 | 0.1276 | 0.1596 | 0.1396 |
| 973 | I | 1 | 1 | 1 | 0 |  |  | 0 | 0.139 | 0.1839 | 0.1479 |
| 974 | S | 1 | 1 | 1 | 0 |  |  | 0 | 0.1296 | 0.1846 | 0.1458 |
| 975 | S | 1 | 1 | 1 | 0 |  |  | 0 | 0.1322 | 0.1822 | 0.1406 |
| 976 | V | 2 | 2 | 2 | 0 |  |  | 0 | 0.13 | 0.2137 | 0.137 |
| 977 | L | 1 | 1 | 1 | 0 |  |  | 0 | 0.1146 | 0.2308 | 0.1211 |
| 978 | N | 1 | 1 | 1 | 0 |  |  | 0 | 0.1261 | 0.2681 | 0.1403 |
| 979 | D | 1 | 1 | 1 | 0 |  |  | 0 | 0.1141 | 0.2578 | 0.1494 |
| 980 | I | 2 | 1 | 1 | 0 |  |  | 0 | 0.104 | 0.232 | 0.1332 |
| 981 | L | 1 | 1 | 1 | 0 |  |  | 1 | 0.1161 | 0.2688 | 0.1311 |
| 982 | S | 1 | 1 | 1 | 0 |  |  | 2 | 0.125 | 0.3037 | 0.1522 |
| 983 | R | 1 | 1 | 1 | 0 |  |  | 2 | 0.1173 | 0.2801 | 0.1473 |
| 984 | L | 1 | 1 | 1 | 0 |  |  | 3 | 0.1248 | 0.2698 | 0.1318 |
| 985 | D | 1 | 1 | 1 | 0 |  |  | 3 | 0.1685 | 0.3197 | 0.1445 |
| 986 | K | 1 | 1 | 1 | 0 |  |  | 3 | 0.1927 | 0.3352 | 0.1647 |
| 987 | V | 1 | 1 | 1 | 0 |  |  | 4 | 0.2044 | 0.3292 | 0.1713 |
| 988 | E | 1 | 1 | 1 | 0 |  |  | 6 | 0.1719 | 0.2679 | 0.1404 |
| 989 | A | 1 | 1 | 1 | 0 |  |  | 5 | 0.1133 | 0.2395 | 0.1219 |
| 990 | E | 1 | 1 | 1 | 0 |  |  | 5 | 0.121 | 0.2476 | 0.1293 |
| 991 | V | 1 | 1 | 1 | 0 |  |  | 4 | 0.1517 | 0.2085 | 0.1323 |
| 992 | Q | 1 | 1 | 1 | 0 |  |  | 3 | 0.1363 | 0.1808 | 0.1177 |
| 993 | I | 1 | 1 | 1 | 0 |  |  | 0 | 0.1073 | 0.1877 | 0.1069 |
| 994 | D | 1 | 1 | 1 | 0 |  |  | 0 | 0.1058 | 0.1672 | 0.1083 |
| 995 | R | 1 | 1 | 1 | 0 |  |  | 0 | 0.1098 | 0.1424 | 0.1117 |
| 996 | L | 1 | 1 | 1 | 0 |  |  | 0 | 0.1011 | 0.1519 | 0.1033 |
| 997 | I | 1 | 1 | 1 | 0 |  |  | 0 | 0.1011 | 0.1508 | 0.0959 |
| 998 | T | 1 | 1 | 1 | 0 |  |  | 0 | 0.103 | 0.134 | 0.1034 |
| 999 | G | 1 | 1 | 1 | 0 |  |  | 0 | 0.1045 | 0.1274 | 0.1081 |
| 1000 | R | 1 | 1 | 1 | 0 |  |  | 0 | 0.1032 | 0.1292 | 0.1023 |
| 1001 | L | 1 | 1 | 1 | 0 |  |  | 0 | 0.0994 | 0.125 | 0.1047 |
| 1002 | Q | 1 | 1 | 1 | 0 |  |  | 0 | 0.105 | 0.117 | 0.1165 |
| 1003 | S | 1 | 1 | 1 | 0 |  |  | 0 | 0.1074 | 0.1212 | 0.1064 |
| 1004 | L | 1 | 1 | 1 | 0 |  |  | 0 | 0.1057 | 0.12 | 0.1059 |
| 1005 | Q | 1 | 1 | 1 | 0 |  |  | 0 | 0.1059 | 0.1163 | 0.1142 |
| 1006 | T | 1 | 1 | 1 | 0 |  |  | 0 | 0.103 | 0.1129 | 0.1127 |
| 1007 | Y | 1 | 1 | 1 | 0 |  |  | 0 | 0.093 | 0.1036 | 0.1012 |
| 1008 | V | 1 | 1 | 1 | 0 |  |  | 0 | 0.097 | 0.1079 | 0.1045 |
| 1009 | T | 1 | 1 | 1 | 0 |  |  | 0 | 0.0968 | 0.1096 | 0.1048 |
| 1010 | Q | 1 | 1 | 1 | 0 |  |  | 0 | 0.092 | 0.1013 | 0.0974 |
| 1011 | Q | 1 | 1 | 1 | 0 |  |  | 0 | 0.0956 | 0.1012 | 0.1007 |
| 1012 | L | 1 | 1 | 1 | 0 |  |  | 0 | 0.1027 | 0.1046 | 0.1018 |
| 1013 | I | 1 | 1 | 1 | 0 |  |  | 0 | 0.1046 | 0.1082 | 0.1 |
| 1014 | R | 1 | 1 | 1 | 0 |  |  | 0 | 0.0987 | 0.1111 | 0.1029 |
| 1015 | A | 1 | 1 | 1 | 0 |  |  | 0 | 0.1064 | 0.1128 | 0.1074 |
| 1016 | A | 1 | 1 | 1 | 0 |  |  | 0 | 0.1149 | 0.1166 | 0.1116 |
| 1017 | E | 1 | 1 | 1 | 0 |  |  | 0 | 0.1047 | 0.1012 | 0.1096 |
| 1018 | I | 1 | 1 | 1 | 0 |  |  | 0 | 0.0969 | 0.0997 | 0.102 |
| 1019 | R | 2 | 1 | 1 | 0 |  |  | 0 | 0.1173 | 0.1108 | 0.1036 |
| 1020 | A | 2 | 3 | 3 | 0 |  |  | 0 | 0.1159 | 0.1042 | 0.1045 |
| 1021 | S | 1 | 1 | 1 | 0 |  |  | 0 | 0.0982 | 0.0962 | 0.098 |
| 1022 | A | 1 | 1 | 1 | 0 |  |  | 0 | 0.106 | 0.1054 | 0.095 |
| 1023 | N | 1 | 1 | 1 | 0 |  |  | 0 | 0.1217 | 0.1087 | 0.0992 |
| 1024 | L | 1 | 1 | 1 | 0 |  |  | 0 | 0.1115 | 0.0968 | 0.0983 |
| 1025 | A | 1 | 1 | 1 | 0 |  |  | 0 | 0.101 | 0.0938 | 0.0947 |
| 1026 | A | 1 | 1 | 1 | 0 |  |  | 0 | 0.1214 | 0.1007 | 0.0993 |
| 1027 | T | 1 | 1 | 1 | 0 |  |  | 0 | 0.1287 | 0.1008 | 0.1043 |
| 1028 | K | 1 | 1 | 1 | 0 |  |  | 0 | 0.1092 | 0.0966 | 0.1023 |
| 1029 | M | 1 | 1 | 1 | 0 |  |  | 0 | 0.1129 | 0.0951 | 0.1003 |
| 1030 | S | 1 | 1 | 1 | 0 |  |  | 0 | 0.1345 | 0.087 | 0.1027 |
| 1031 | E | 1 | 1 | 1 | 0 |  |  | 0 | 0.1045 | 0.0905 | 0.1035 |
| 1032 | C | 1 | 1 | 1 | 0 |  |  | 0 | 0.0942 | 0.0952 | 0.0996 |
| 1033 | V | 1 | 1 | 1 | 0 |  |  | 0 | 0.0938 | 0.0966 | 0.1006 |
| 1034 | L | 2 | 1 | 1 | 0 |  |  | 0 | 0.1057 | 0.1007 | 0.1055 |
| 1035 | G | 1 | 1 | 1 | 0 |  |  | 0 | 0.1155 | 0.1097 | 0.1185 |
| 1036 | Q | 1 | 1 | 1 | 0 |  |  | 0 | 0.1183 | 0.1369 | 0.1124 |
| 1037 | S | 1 | 1 | 1 | 0 |  |  | 0 | 0.1066 | 0.1258 | 0.1109 |
| 1038 | K | 1 | 1 | 1 | 0 |  |  | 0 | 0.1674 | 0.1338 | 0.142 |
| 1039 | R | 1 | 1 | 1 | 0 |  |  | 0 | 0.1673 | 0.1439 | 0.1602 |
| 1040 | V | 1 | 2 | 2 | 0 |  |  | 0 | 0.1806 | 0.141 | 0.1554 |
| 1041 | D | 2 | 1 | 1 | 0 |  |  | 0 | 0.1391 | 0.1469 | 0.1503 |
| 1042 | F | 1 | 1 | 1 | 0 |  |  | 0 | 0.0973 | 0.1384 | 0.1352 |
| 1043 | C | 1 | 1 | 1 | 0 |  |  | 0 | 0.102 | 0.1224 | 0.1355 |
| 1044 | G | 1 | 1 | 1 | 0 |  |  | 0 | 0.1287 | 0.1281 | 0.1591 |
| 1045 | K | 2 | 1 | 1 | 0 |  |  | 0 | 0.1481 | 0.1281 | 0.1755 |
| 1046 | G | 1 | 1 | 1 | 0 |  |  | 0 | 0.1612 | 0.1041 | 0.1373 |
| 1047 | Y | 1 | 1 | 1 | 0 |  |  | 0 | 0.1098 | 0.0951 | 0.1261 |
| 1048 | H | 1 | 1 | 1 | 0 |  |  | 0 | 0.0927 | 0.0957 | 0.1167 |
| 1049 | L | 1 | 1 | 1 | 0 |  |  | 0 | 0.0991 | 0.0995 | 0.1178 |
| 1050 | M | 1 | 1 | 1 | 0 |  |  | 0 | 0.0999 | 0.1055 | 0.1137 |
| 1051 | S | 1 | 1 | 1 | 0 |  |  | 0 | 0.0822 | 0.1001 | 0.1083 |
| 1052 | F | 1 | 1 | 1 | 0 |  |  | 0 | 0.087 | 0.1044 | 0.1115 |
| 1053 | P | 1 | 1 | 1 | 0 |  |  | 0 | 0.0849 | 0.1092 | 0.1098 |
| 1054 | Q | 1 | 1 | 1 | 0 |  |  | 0 | 0.0883 | 0.1046 | 0.1108 |
| 1055 | S | 2 | 1 | 1 | 0 |  |  | 0 | 0.0897 | 0.1199 | 0.116 |
| 1056 | A | 1 | 2 | 2 | 0 |  |  | 0 | 0.0998 | 0.1133 | 0.1106 |
| 1057 | P | 1 | 1 | 1 | 0 |  |  | 0 | 0.1175 | 0.1186 | 0.1198 |
| 1058 | H | 1 | 2 | 2 | 0 |  |  | 0 | 0.0975 | 0.1241 | 0.1155 |
| 1059 | G | 1 | 1 | 1 | 0 |  |  | 0 | 0.0952 | 0.1228 | 0.1138 |
| 1060 | V | 1 | 2 | 2 | 0 |  |  | 0 | 0.0884 | 0.1071 | 0.1129 |
| 1061 | V | 1 | 1 | 1 | 0 |  |  | 0 | 0.0864 | 0.1063 | 0.1139 |
| 1062 | F | 1 | 1 | 1 | 0 |  |  | 0 | 0.0808 | 0.0967 | 0.109 |
| 1063 | L | 1 | 2 | 2 | 0 |  |  | 0 | 0.0869 | 0.0971 | 0.1149 |
| 1064 | H | 1 | 1 | 1 | 0 |  |  | 0 | 0.084 | 0.1023 | 0.1135 |
| 1065 | V | 1 | 2 | 2 | 0 |  |  | 0 | 0.0961 | 0.0962 | 0.1189 |
| 1066 | T | 1 | 2 | 2 | 0 |  |  | 0 | 0.1 | 0.0887 | 0.1237 |
| 1067 | Y | 2 | 2 | 2 | 0 |  |  | 1 | 0.1116 | 0.0876 | 0.1268 |
| 1068 | V | 2 | 1 | 1 | 0 |  |  | 3 | 0.1216 | 0.0948 | 0.1224 |
| 1069 | P | 1 | 1 | 1 | 0 |  |  | 3 | 0.1343 | 0.1013 | 0.1352 |
| 1070 | A | 3 | 1 | 1 | 0 |  |  | 4 | 0.1456 | 0.1178 | 0.152 |
| 1071 | Q | 2 | 1 | 1 | 0 |  |  | 4 | 0.1543 | 0.1394 | 0.1711 |
| 1072 | E | 1 | 1 | 1 | 0 |  |  | 4 | 0.1489 | 0.146 | 0.1771 |
| 1073 | K | 3 | 1 | 1 | 0 |  |  | 2 | 0.1515 | 0.147 | 0.1961 |
| 1074 | N | 1 | 1 | 1 | 0 |  |  | 2 | 0.1439 | 0.1518 | 0.1826 |
| 1075 | F | 1 | 1 | 1 | 0 |  |  | 1 | 0.1433 | 0.1417 | 0.1719 |
| 1076 | T | 1 | 2 | 2 | 0 |  |  | 2 | 0.1428 | 0.1461 | 0.1675 |
| 1077 | T | 1 | 1 | 1 | 0 |  |  | 1 | 0.1385 | 0.143 | 0.1747 |
| 1078 | A | 1 | 3 | 3 | 0 |  |  | 1 | 0.1736 | 0.173 | 0.1728 |
| 1079 | P | 1 | 3 | 3 | 0 |  |  | 1 | 0.2514 | 0.216 | 0.23 |
| 1080 | A | 1 | 1 | 1 | 0 |  |  | 1 | 0.1862 | 0.1626 | 0.2018 |
| 1081 | I | 1 | 1 | 1 | 0 |  |  | 0 | 0.1635 | 0.1982 | 0.1925 |
| 1082 | C | 1 | 1 | 1 | 0 |  |  | 0 | 0.1779 | 0.238 | 0.2278 |
| 1083 | H | 1 | 1 | 1 | 0 |  |  | 0 | 0.2005 | 0.2802 | 0.3046 |
| 1084 | D | 3 | 2 | 3 | 1 |  |  | 0 | 0.2009 | 0.3712 | 0.3244 |
| 1085 | G | 1 | 1 | 1 | 0 |  |  | 0 | 0.1893 | 0.2935 | 0.3294 |
| 1086 | K | 1 | 3 | 3 | 0 |  |  | 0 | 0.166 | 0.2441 | 0.3086 |
| 1087 | A | 1 | 2 | 2 | 0 |  |  | 0 | 0.1666 | 0.194 | 0.2365 |
| 1088 | H | 2 | 1 | 1 | 0 |  |  | 0 | 0.155 | 0.1696 | 0.2281 |
| 1089 | F | 1 | 1 | 1 | 0 |  |  | 0 | 0.161 | 0.1339 | 0.2071 |
| 1090 | P | 1 | 1 | 1 | 0 |  |  | 0 | 0.1533 | 0.1381 | 0.1899 |
| 1091 | R | 1 | 2 | 2 | 0 |  |  | 0 | 0.1715 | 0.1412 | 0.1966 |
| 1092 | E | 1 | 1 | 1 | 0 |  |  | 0 | 0.1848 | 0.1496 | 0.1806 |
| 1093 | G | 1 | 2 | 2 | 0 |  |  | 0 | 0.1525 | 0.1393 | 0.1642 |
| 1094 | V | 1 | 1 | 1 | 0 |  |  | 1 | 0.1605 | 0.1285 | 0.1675 |
| 1095 | F | 1 | 1 | 1 | 0 |  |  | 1 | 0.1558 | 0.1299 | 0.1761 |
| 1096 | V | 1 | 1 | 1 | 0 |  |  | 2 | 0.1431 | 0.1356 | 0.1706 |
| 1097 | S | 3 | 1 | 1 | 0 |  |  | 3 | 0.1546 | 0.1734 | 0.1712 |
| 1098 | N | 2 | 1 | 1 | 0 |  |  | 3 | 0.2005 | 0.2091 | 0.1871 |
| 1099 | G | 1 | 1 | 1 | 0 |  |  | 2 | 0.2961 | 0.327 | 0.2389 |
| 1100 | T | 2 | 1 | 1 | 0 |  |  | 2 | 0.2807 | 0.3239 | 0.2308 |
| 1101 | H | 3 | 2 | 2 | 0 |  |  | 1 | 0.2005 | 0.1927 | 0.1872 |
| 1102 | W | 1 | 1 | 1 | 0 |  |  | 0 | 0.1629 | 0.1719 | 0.1784 |
| 1103 | F | 1 | 1 | 1 | 0 |  |  | 0 | 0.1588 | 0.1399 | 0.169 |
| 1104 | V | 2 | 1 | 2 | 1 |  |  | 0 | 0.1493 | 0.1283 | 0.1687 |
| 1105 | T | 1 | 1 | 1 | 0 |  |  | 0 | 0.1465 | 0.1223 | 0.1626 |
| 1106 | Q | 1 | 1 | 1 | 0 |  |  | 0 | 0.1539 | 0.1185 | 0.1517 |
| 1107 | R | 1 | 1 | 1 | 0 |  |  | 0 | 0.1606 | 0.121 | 0.1513 |
| 1108 | N | 1 | 1 | 1 | 0 |  |  | 0 | 0.1495 | 0.1209 | 0.1473 |
| 1109 | F | 1 | 2 | 2 | 0 |  |  | 0 | 0.1513 | 0.1183 | 0.164 |
| 1110 | Y | 2 | 1 | 1 | 0 |  |  | 1 | 0.1544 | 0.1263 | 0.1706 |
| 1111 | E | 2 | 1 | 1 | 0 |  |  | 1 | 0.1691 | 0.1296 | 0.1752 |
| 1112 | P | 1 | 2 | 2 | 0 |  |  | 1 | 0.1734 | 0.137 | 0.1789 |
| 1113 | Q | 2 | 1 | 1 | 0 |  |  | 4 | 0.1792 | 0.1355 | 0.1804 |
| 1114 | I | 4 | 1 | 1 | 0 |  |  | 5 | 0.1876 | 0.1535 | 0.1891 |
| 1115 | I | 1 | 1 | 1 | 0 |  |  | 4 | 0.1774 | 0.1524 | 0.1941 |
| 1116 | T | 1 | 1 | 1 | 0 |  |  | 4 | 0.2058 | 0.1719 | 0.2164 |
| 1117 | T | 1 | 2 | 2 | 0 |  |  | 4 | 0.1979 | 0.2041 | 0.2132 |
| 1118 | D | 1 | 1 | 1 | 0 |  |  | 1 | 0.1978 | 0.2035 | 0.2524 |
| 1119 | N | 1 | 1 | 1 | 0 |  |  | 0 | 0.1728 | 0.1546 | 0.2497 |
| 1120 | T | 1 | 1 | 1 | 0 |  |  | 0 | 0.1606 | 0.1519 | 0.2221 |
| 1121 | F | 1 | 1 | 1 | 0 |  |  | 0 | 0.1658 | 0.1678 | 0.2501 |
| 1122 | V | 1 | 2 | 2 | 0 |  |  | 0 | 0.1694 | 0.1928 | 0.2868 |
| 1123 | S | 2 | 1 | 1 | 0 |  |  | 0 | 0.2033 | 0.1756 | 0.2916 |
| 1124 | G | 1 | 2 | 2 | 0 |  |  | 0 | 0.2012 | 0.2127 | 0.3285 |
| 1125 | N | 3 | 1 | 2 | 1 |  |  | 1 | 0.1784 | 0.2479 | 0.315 |
| 1126 | C | 1 | 1 | 1 | 0 |  |  | 1 | 0.1757 | 0.249 | 0.2707 |
| 1127 | D | 2 | 1 | 1 | 0 |  |  | 2 | 0.2016 | 0.297 | 0.3099 |
| 1128 | V | 1 | 1 | 1 | 0 |  |  | 7 | 0.201 | 0.2788 | 0.3101 |
| 1129 | V | 1 | 2 | 2 | 0 |  |  | 9 | 0.1751 | 0.2377 | 0.2651 |
| 1130 | I | 1 | 1 | 1 | 0 |  |  | 10 | 0.1898 | 0.2562 | 0.2911 |
| 1131 | G | 1 | 1 | 1 | 0 |  |  | 11 | 0.204 | 0.3187 | 0.2347 |
| 1132 | I | 1 | 2 | 2 | 0 |  |  | 10 | 0.1903 | 0.2435 | 0.2032 |
| 1133 | V | 2 | 2 | 2 | 0 |  |  | 6 | 0.1903 | 0.2623 | 0.2018 |
| 1134 | N | 1 | 1 | 1 | 0 |  |  | 4 | 0.1987 | 0.2932 | 0.2046 |
| 1135 | N | 1 | 1 | 1 | 0 |  |  | 3 | 0.1924 | 0.2615 | 0.2111 |
| 1136 | T | 1 | 1 | 1 | 0 |  |  | 2 | 0.1988 | 0.2526 | 0.2178 |
| 1137 | V | 1 | 1 | 1 | 0 |  |  | 6 | 0.189 | 0.2125 | 0.1937 |
| 1138 | Y | 1 | 1 | 1 | 0 |  |  | 6 | 0.2158 | 0.2048 | 0.2071 |
| 1139 | D | 1 | 2 | 2 | 0 |  |  | 7 | 0.2174 | 0.2218 | 0.2439 |
| 1140 | P | 1 | 1 | 1 | 0 |  |  | 7 | 0.2411 | 0.2352 | 0.2469 |
| 1141 | L | 1 | 1 | 1 | 0 |  |  | 8 | 0.238 | 0.2466 | 0.3006 |
| 1142 | Q | 1 | 1 | 1 | 0 |  |  | 5 | 0.2454 | 0.2859 | 0.3552 |
| 1143 | P | 1 | 2 | 2 | 0 |  |  | 4 | 0.2857 | 0.3125 | 0.3511 |
| 1144 | E | 1 | 1 | 1 | 0 |  |  | 4 | 0.3226 | 0.324 | 0.3694 |
| 1145 | L | 1 | 1 | 1 | 0 |  |  | 3 | 0.3469 | 0.38 | 0.5183 |
| 1146 | D | 2 | 2 | 2 | 0 |  |  | 3 | 0.4349 | 0.4712 | 0.7101 |
| 1147 | S | 1 | 2 | 2 | 0 |  |  | 2 |  |  |  |
| 1148 | F | 1 | 1 | 1 | 0 |  |  | 3 |  |  |  |
| 1149 | K | 1 | 2 | 2 | 0 |  |  | 7 |  |  |  |
| 1150 | E | 4 | 2 | 2 | 0 |  |  | 9 |  |  |  |
| 1151 | E | 1 | 1 | 1 | 0 |  |  | 8 |  |  |  |
| 1152 | L | 1 | 1 | 1 | 0 |  |  | 9 |  |  |  |
| 1153 | D | 1 | 1 | 1 | 0 |  |  | 8 |  |  |  |
| 1154 | K | 1 | 1 | 1 | 0 |  |  | 4 |  |  |  |
| 1155 | Y | 1 | 1 | 1 | 0 |  |  | 2 |  |  |  |
| 1156 | F | 1 | 1 | 1 | 0 |  |  | 2 |  |  |  |
| 1157 | K | 1 | 2 | 2 | 0 |  |  | 3 |  |  |  |
| 1158 | N | 2 | 1 | 1 | 0 |  |  | 3 |  |  |  |
| 1159 | H | 1 | 1 | 1 | 0 |  |  | 2 |  |  |  |
| 1160 | T | 1 | 1 | 1 | 0 |  |  | 2 |  |  |  |
| 1161 | S | 1 | 1 | 1 | 0 |  |  | 3 |  |  |  |
| 1162 | P | 2 | 4 | 4 | 0 |  |  | 2 |  |  |  |
| 1163 | D | 2 | 2 | 2 | 0 |  |  | 5 |  |  |  |
| 1164 | V | 2 | 1 | 1 | 0 |  |  | 6 |  |  |  |
| 1165 | D | 2 | 2 | 2 | 0 |  |  | 7 |  |  |  |
| 1166 | L | 2 | 1 | 1 | 0 |  |  | 9 |  |  |  |
| 1167 | G | 2 | 2 | 2 | 0 |  |  | 10 |  |  |  |
| 1168 | D | 2 | 2 | 2 | 0 |  |  | 7 |  |  |  |
| 1169 | I | 2 | 3 | 3 | 0 |  |  | 5 |  |  |  |
| 1170 | S | 2 | 2 | 2 | 0 |  |  | 7 |  |  |  |
| 1171 | G | 3 | 1 | 1 | 0 |  |  | 5 |  |  |  |
| 1172 | I | 1 | 1 | 1 | 0 |  |  | 6 |  |  |  |
| 1173 | N | 1 | 1 | 1 | 0 |  |  | 11 |  |  |  |
| 1174 | A | 2 | 1 | 1 | 0 |  |  | 14 |  |  |  |
| 1175 | S | 2 | 1 | 1 | 0 |  |  | 12 |  |  |  |
| 1176 | V | 1 | 2 | 2 | 0 |  |  | 12 |  |  |  |
| 1177 | V | 1 | 2 | 2 | 0 |  |  | 12 |  |  |  |
| 1178 | N | 2 | 1 | 1 | 0 |  |  | 8 |  |  |  |
| 1179 | I | 1 | 1 | 1 | 0 |  |  | 4 |  |  |  |
| 1180 | Q | 2 | 1 | 1 | 0 |  |  | 3 |  |  |  |
| 1181 | K | 2 | 1 | 1 | 0 |  |  | 3 |  |  |  |
| 1182 | E | 1 | 2 | 2 | 0 |  |  | 4 |  |  |  |
| 1183 | I | 1 | 1 | 1 | 0 |  |  | 4 |  |  |  |
| 1184 | D | 3 | 1 | 1 | 0 |  |  | 6 |  |  |  |
| 1185 | R | 2 | 2 | 2 | 0 |  |  | 6 |  |  |  |
| 1186 | L | 1 | 1 | 1 | 0 |  |  | 5 |  |  |  |
| 1187 | N | 1 | 1 | 1 | 0 |  |  | 3 |  |  |  |
| 1188 | E | 1 | 1 | 1 | 0 |  |  | 4 |  |  |  |
| 1189 | V | 2 | 1 | 1 | 0 |  |  | 2 |  |  |  |
| 1190 | A | 1 | 1 | 1 | 0 |  |  | 1 |  |  |  |
| 1191 | K | 2 | 2 | 2 | 0 |  |  | 2 |  |  |  |
| 1192 | N | 2 | 1 | 1 | 0 |  |  | 3 |  |  |  |
| 1193 | L | 1 | 1 | 1 | 0 |  |  | 3 |  |  |  |
| 1194 | N | 1 | 2 | 2 | 0 |  |  | 3 |  |  |  |
| 1195 | E | 2 | 1 | 1 | 0 |  |  | 4 |  |  |  |
| 1196 | S | 1 | 1 | 1 | 0 |  |  | 4 |  |  |  |
| 1197 | L | 2 | 1 | 2 | 1 |  |  | 5 |  |  |  |
| 1198 | I | 1 | 1 | 1 | 0 |  |  | 4 |  |  |  |
| 1199 | D | 1 | 1 | 1 | 0 |  |  | 9 |  |  |  |
| 1200 | L | 1 | 1 | 1 | 0 |  |  | 9 |  |  |  |
| 1201 | Q | 2 | 1 | 1 | 0 |  |  | 9 |  |  |  |
| 1202 | E | 1 | 2 | 2 | 0 |  |  | 7 |  |  |  |
| 1203 | L | 1 | 2 | 2 | 0 |  |  | 7 |  |  |  |
| 1204 | G | 1 | 1 | 1 | 0 |  |  | 3 |  |  |  |
| 1205 | K | 1 | 1 | 1 | 0 |  |  | 1 |  |  |  |
| 1206 | Y | 1 | 1 | 1 | 0 |  |  | 0 |  |  |  |
| 1207 | E | 1 | 3 | 3 | 0 |  |  | 0 |  |  |  |
| 1208 | Q | 2 | 1 | 1 | 0 |  |  | 0 |  |  |  |
| 1209 | Y | 1 | 2 | 2 | 0 |  |  | 0 |  |  |  |
| 1210 | I | 1 | 2 | 2 | 0 |  |  | 0 |  |  |  |
| 1211 | K | 1 | 1 | 1 | 0 |  |  | 0 |  |  |  |
| 1212 | W | 1 | 1 | 1 | 0 |  |  | 0 |  |  |  |
| 1213 | P | 1 | 1 | 1 | 0 |  |  | 0 |  |  |  |
| 1214 | W | 1 | 1 | 1 | 0 |  |  | 0 |  |  |  |
| 1215 | Y | 1 | 1 | 1 | 0 |  |  | 0 |  |  |  |
| 1216 | I | 2 | 2 | 2 | 0 |  |  | 0 |  |  |  |
| 1217 | W | 1 | 1 | 1 | 0 |  |  | 0 |  |  |  |
| 1218 | L | 1 | 1 | 1 | 0 |  |  | 0 |  |  |  |
| 1219 | G | 1 | 3 | 3 | 0 |  |  | 1 |  |  |  |
| 1220 | F | 1 | 1 | 1 | 0 |  |  | 1 |  |  |  |
| 1221 | I | 1 | 1 | 1 | 0 |  |  | 2 |  |  |  |
| 1222 | A | 1 | 1 | 1 | 0 |  |  | 3 |  |  |  |
| 1223 | G | 1 | 1 | 1 | 0 |  |  | 4 |  |  |  |
| 1224 | L | 1 | 1 | 1 | 0 |  |  | 3 |  |  |  |
| 1225 | I | 3 | 1 | 1 | 0 |  |  | 3 |  |  |  |
| 1226 | A | 3 | 1 | 1 | 0 |  |  | 2 |  |  |  |
| 1227 | I | 2 | 1 | 1 | 0 |  |  | 4 |  |  |  |
| 1228 | V | 3 | 2 | 3 | 1 |  |  | 4 |  |  |  |
| 1229 | M | 2 | 2 | 2 | 0 |  |  | 6 |  |  |  |
| 1230 | V | 2 | 2 | 2 | 0 |  |  | 7 |  |  |  |
| 1231 | T | 2 | 1 | 1 | 0 |  |  | 8 |  |  |  |
| 1232 | I | 1 | 2 | 2 | 0 |  |  | 6 |  |  |  |
| 1233 | M | 2 | 1 | 1 | 0 |  |  | 5 |  |  |  |
| 1234 | L | 1 | 1 | 1 | 0 |  |  | 3 |  |  |  |
| 1235 | C | 1 | 1 | 1 | 0 |  |  | 2 |  |  |  |
| 1236 | C | 2 | 2 | 2 | 0 |  |  | 1 |  |  |  |
| 1237 | M | 3 | 2 | 2 | 0 |  |  | 1 |  |  |  |
| 1238 | T | 1 | 2 | 2 | 0 |  |  | 0 |  |  |  |
| 1239 | S | 1 | 1 | 1 | 0 |  |  | 0 |  |  |  |
| 1240 | C | 1 | 1 | 1 | 0 |  |  | 0 |  |  |  |
| 1241 | C | 1 | 1 | 1 | 0 |  |  | 0 |  |  |  |
| 1242 | S | 1 | 1 | 1 | 0 |  |  | 0 |  |  |  |
| 1243 | C | 1 | 1 | 1 | 0 |  |  | 0 |  |  |  |
| 1244 | L | 2 | 1 | 1 | 0 |  |  | 1 |  |  |  |
| 1245 | K | 2 | 2 | 2 | 0 |  |  | 1 |  |  |  |
| 1246 | G | 1 | 1 | 1 | 0 |  |  | 1 |  |  |  |
| 1247 | C | 4 | 2 | 2 | 0 |  |  | 1 |  |  |  |
| 1248 | C | 1 | 2 | 2 | 0 |  |  | 1 |  |  |  |
| 1249 | S | 1 | 1 | 1 | 0 |  |  | 1 |  |  |  |
| 1250 | C | 1 | 3 | 3 | 0 |  |  | 1 |  |  |  |
| 1251 | G | 2 | 1 | 1 | 0 |  |  | 1 |  |  |  |
| 1252 | S | 2 | 2 | 2 | 0 |  |  | 3 |  |  |  |
| 1253 | C | 1 | 1 | 1 | 0 |  |  | 3 |  |  |  |
| 1254 | C | 1 | 3 | 3 | 0 |  |  | 2 |  |  |  |
| 1255 | K | 2 | 1 | 1 | 0 |  |  | 4 |  |  |  |
| 1256 | F | 1 | 1 | 1 | 0 |  |  | 6 |  |  |  |
| 1257 | D | 1 | 1 | 1 | 0 |  |  | 5 |  |  |  |
| 1258 | E | 1 | 2 | 2 | 0 |  |  | 7 |  |  |  |
| 1259 | D | 1 | 2 | 2 | 0 |  |  | 8 |  |  |  |
| 1260 | D | 2 | 2 | 2 | 0 |  |  | 8 |  |  |  |
| 1261 | S | 2 | 1 | 1 | 0 |  |  | 7 |  |  |  |
| 1262 | E | 1 | 2 | 2 | 0 |  |  | 6 |  |  |  |
| 1263 | P | 1 | 2 | 2 | 0 |  |  | 4 |  |  |  |
| 1264 | V | 2 | 2 | 2 | 0 |  |  | 4 |  |  |  |
| 1265 | L | 1 | 2 | 2 | 0 |  |  | 3 |  |  |  |
| 1266 | K | 2 | 1 | 1 | 0 |  |  | 2 |  |  |  |
| 1267 | G | 1 | 1 | 1 | 0 |  |  | 2 |  |  |  |
| 1268 | V | 1 | 1 | 1 | 0 |  |  | 2 |  |  |  |
| 1269 | K | 1 | 1 | 1 | 0 |  |  | 1 |  |  |  |
| 1270 | L | 1 | 1 | 1 | 0 |  |  | 0 |  |  |  |
| 1271 | H | 1 | 1 | 1 | 0 |  |  | 0 |  |  |  |
| 1272 | Y | 1 | 1 | 1 | 0 |  |  | 0 |  |  |  |
| 1273 | T | 1 | 2 | 2 | 0 |  |  | 0 |  |  |  |

Table 2. Human cell surface protein with local sequence similarity to S protein domains

| Uniprot ACC | Entry name | Alignment position | Alignment length | Query seq | Hit seq |
| --- | --- | --- | --- | --- | --- |
| Q9UQV4 | LAMP3_HUMAN | 457-461 | 5 | RKSNL | RKSNL |
| P07339 | CATD_HUMAN | 405-409 | 5 | DEVRQ | DEVRE |
| P02751 | FINC_HUMAN | 624-628 | 5 | IHADQ | VHADQ |
| P02751 | FINC_HUMAN | 681-685 | 5 | PRRAR | PRRAR |
| P02751 | FINC_HUMAN | 583-587 | 5 | EILDI | EILDV |
| P02751 | FINC_HUMAN | 549-553 | 5 | TGVLT | TGVLT |
| P04440 | DPB1_HUMAN | 530-534 | 5 | STNLV | STNLI |
| P61978 | HNRPK_HUMAN | 866-870 | 5 | TDEMI | TDEMV |
| P11279 | LAMP1_HUMAN | 70-74 | 5 | VSGTN | VSGTN |
| P11279 | LAMP1_HUMAN | 71-75 | 5 | SGTNG | SGTNG |
| P11279 | LAMP1_HUMAN | 72-76 | 5 | GTNGT | GTNGT |
| P11279 | LAMP1_HUMAN | 343-347 | 5 | NATRF | NATRY |
| P39023 | RL3_HUMAN | 808-812 | 5 | DPSKP | DPSKP |
| P39023 | RL3_HUMAN | 1128-1132 | 5 | VVIGI | VVVGI |
| P39023 | RL3_HUMAN | 237-241 | 5 | RFQTL | RFQTM |
| P06733 | ENOA_HUMAN | 122-126 | 5 | NATNV | DATNV |
| Q07020 | RL18_HUMAN | 283-287 | 5 | GTITD | GTITD |
| P05556 | ITB1_HUMAN | 339-343 | 5 | GEVFN | GEVFN |
| P05556 | ITB1_HUMAN | 175-179 | 5 | FLMDL | YLMDL |
| P05556 | ITB1_HUMAN | 936-940 | 5 | DSLSS | NSLSS |
| P07900 | HS90A_HUMAN | 147-151 | 5 | KNNKS | KNDKS |
| Q08722 | CD47_HUMAN | 821-825 | 5 | LLFNK | LLFNK |
| P12956 | XRCC6_HUMAN | 80-84 | 5 | DNPVL | ENPVL |
| P04843 | RPN1_HUMAN | 466-470 | 5 | RDIST | RDIST |
| P04843 | RPN1_HUMAN | 918-922 | 5 | ENQKL | ENEKL |
| P04843 | RPN1_HUMAN | 783-787 | 5 | AQVKQ | AQVKE |
| O14786 | NRP1_HUMAN | 218-222 | 5 | QGFSA | EGFSA |
| O14786 | NRP1_HUMAN | 364-368 | 5 | DYSVL | NYSVL |
| Q9UBG0 | MRC2_HUMAN | 600-604 | 5 | PGTNT | PGTNT |
| Q00839 | HNRPU_HUMAN | 843-847 | 5 | DIAAR | EIAAR |
| Q9NV96 | CC50A_HUMAN | 138-142 | 5 | DPFLG | NPFLG |
| P54709 | AT1B3_HUMAN | 268-272 | 5 | GYLQP | GYLQP |
| P54709 | AT1B3_HUMAN | 267-271 | 5 | VGYLQ | VGYLQ |
| P54709 | AT1B3_HUMAN | 817-821 | 5 | FIEDL | YIEDL |
| P20645 | MPRD_HUMAN | 932-936 | 5 | GKIQD | GKVQD |
| Q9ULI3 | HEG1_HUMAN | 716-720 | 5 | TNFTI | TNFTI |
| Q9ULI3 | HEG1_HUMAN | 939-943 | 5 | SSTAS | SSTAS |
| Q9ULI3 | HEG1_HUMAN | 938-942 | 5 | LSSTA | LSSTA |
| P14314 | GLU2B_HUMAN | 1142-1146 | 5 | QPELD | HPELD |
| Q8IWA5 | CTL2_HUMAN | 156-160 | 5 | EFRVY | DFRVY |
| P07942 | LAMB1_HUMAN | 438-442 | 5 | SNNLD | SNDLD |
| Q13478 | IL18R_HUMAN | 464-468 | 5 | FERDI | FERDV |
| Q9NS15 | LTBP3_HUMAN | 889-893 | 5 | GAGAA | GAGAA |
| Q9NS15 | LTBP3_HUMAN | 159-163 | 5 | VYSSA | VYSSA |
| P40189 | IL6RB_HUMAN | 807-811 | 5 | PDPSK | PDPSK |
| P40189 | IL6RB_HUMAN | 460-464 | 5 | NLKPF | DLKPF |
| P54852 | EMP3_HUMAN | 623-627 | 5 | AIHAD | AIHAE |
| Q5T3F8 | CSCL2_HUMAN | 556-560 | 5 | NKKFL | DKKFL |
| Q9BY67 | CADM1_HUMAN | 614-618 | 5 | DVNCT | EVNCT |
| Q08380 | LG3BP_HUMAN | 212-216 | 5 | LVRDL | MVRDL |
| P08195 | 4F2_HUMAN | 482-486 | 5 | GVEGF | GVDGF |
| O95490 | AGRL2_HUMAN | 639-643 | 5 | GSNVF | GSDVF |
| O95490 | AGRL2_HUMAN | 607-611 | 5 | QVAVL | EVAVL |
| P11717 | MPRI_HUMAN | 282-286 | 5 | NGTIT | NGTIT |
| P11717 | MPRI_HUMAN | 283-287 | 5 | GTITD | GTITN |
| Q9UQ80 | PA2G4_HUMAN | 414-418 | 5 | QTGKI | ETGKI |
| Q9UQ80 | PA2G4_HUMAN | 914-918 | 5 | NVLYE | NVLYE |
| O43567 | RNF13_HUMAN | 792-796 | 5 | PPIKD | PPVKD |
| O43852 | CALU_HUMAN | 627-631 | 5 | DQLTP | DQLTP |
| P48960 | CD97_HUMAN | 1113-1117 | 5 | QIITT | EIITT |
| O00754 | MA2B1_HUMAN | 1137-1141 | 5 | VYDPL | VYNPL |
| Q8TCJ2 | STT3B_HUMAN | 640-644 | 5 | SNVFQ | SNVFE |
| Q8TCJ2 | STT3B_HUMAN | 503-507 | 5 | VGYQP | VGFQP |
| Q96BD0 | SO4A1_HUMAN | 576-580 | 5 | VRDPQ | VRDPQ |
| Q96BD0 | SO4A1_HUMAN | 213-217 | 5 | VRDLP | IRDLP |
| Q13444 | ADA15_HUMAN | 251-255 | 5 | PGDSS | PGDSS |
| Q9UHG3 | PCYOX_HUMAN | 867-871 | 5 | DEMIA | NEMIA |
| Q92896 | GSLG1_HUMAN | 458-462 | 5 | KSNLK | KSDLK |
| Q92896 | GSLG1_HUMAN | 920-924 | 5 | QKLIA | QKLIA |
| Q969N2 | PIGT_HUMAN | 842-846 | 5 | GDIAA | GDVAA |
| Q16363 | LAMA4_HUMAN | 639-643 | 5 | GSNVF | GSDVF |
| Q16363 | LAMA4_HUMAN | 937-941 | 5 | SLSST | SLSST |
| Q16363 | LAMA4_HUMAN | 938-942 | 5 | LSSTA | LSSTA |
| Q16363 | LAMA4_HUMAN | 428-432 | 5 | DFTGC | NFTGC |
| Q16363 | LAMA4_HUMAN | 685-689 | 5 | RSVAS | RSVAS |
| Q16363 | LAMA4_HUMAN | 798-802 | 5 | GGFNF | GGFNF |
| Q9Y639 | NPTN_HUMAN | 187-191 | 5 | KNLRE | KNLRQ |
| Q4KMQ2 | ANO6_HUMAN | 1129-1133 | 5 | VIGIV | VIGII |
| Q8NBJ5 | GT251_HUMAN | 988-992 | 5 | EAEVQ | QAEVQ |
| O14672 | ADA10_HUMAN | 328-332 | 5 | RFPNI | RFPNI |
| Q13873 | BMPR2_HUMAN | 458-462 | 5 | KSNLK | KSNLK |
| Q13873 | BMPR2_HUMAN | 281-285 | 5 | ENGTI | ENGTI |
| Q07954 | LRP1_HUMAN | 480-484 | 5 | CNGVE | CNGVQ |
| Q07954 | LRP1_HUMAN | 364-368 | 5 | DYSVL | DYSVL |
| Q92542 | NICA_HUMAN | 818-822 | 5 | IEDLL | VEDLL |
| Q07954 | LRP1_HUMAN | 715-719 | 5 | PTNFT | PTNFT |
| Q96JJ7 | TMX3_HUMAN | 1146-1150 | 5 | DSFKE | ESFKE |
| Q96JJ7 | TMX3_HUMAN | 603-607 | 5 | NTSNQ | NTSNQ |
| O96005 | CLPT1_HUMAN | 817-821 | 5 | FIEDL | FIDDL |
| O94813 | SLIT2_HUMAN | 286-290 | 5 | TDAVD | TDAVN |
| O94813 | SLIT2_HUMAN | 916-920 | 5 | LYENQ | LYDNQ |
| Q12797 | ASPH_HUMAN | 1137-1141 | 5 | VYDPL | VYEPL |
| Q12797 | ASPH_HUMAN | 237-241 | 5 | RFQTL | RFETL |
| P11047 | LAMC1_HUMAN | 483-487 | 5 | VEGFN | VEGFN |
| P11047 | LAMC1_HUMAN | 484-488 | 5 | EGFNC | EGFNC |
| P19256 | LFA3_HUMAN | 330-334 | 5 | PNITN | PNITD |
| P53708 | ITA8_HUMAN | 70-74 | 5 | VSGTN | VSGTN |
| P78504 | JAG1_HUMAN | 793-797 | 5 | PIKDF | PIKDY |
| P28827 | PTPRM_HUMAN | 322-326 | 5 | PTESI | PTESI |
| P28827 | PTPRM_HUMAN | 813-817 | 5 | SKRSF | SKRSF |
| P42702 | LIFR_HUMAN | 178-182 | 5 | DLEGK | DLQGK |
| Q92508 | PIEZ1_HUMAN | 530-534 | 5 | STNLV | STNLI |
| O15031 | PLXB2_HUMAN | 80-84 | 5 | DNPVL | ENPVL |
| O60568 | PLOD3_HUMAN | 401-405 | 5 | VIRGD | VIRGD |
| O60568 | PLOD3_HUMAN | 79-83 | 5 | FDNPV | FDNPV |
| Q08431 | MFGM_HUMAN | 182-186 | 5 | KQGNF | KQGNF |
| Q08431 | MFGM_HUMAN | 706-710 | 5 | AYSNN | AYSND |
| Q08431 | MFGM_HUMAN | 705-709 | 5 | VAYSN | VAYSN |
| P06756 | ITAV_HUMAN | 822-826 | 5 | LFNKV | LFDKV |
| P06756 | ITAV_HUMAN | 154-158 | 5 | ESEFR | ESEFR |
| P18084 | ITB5_HUMAN | 532-536 | 5 | NLVKN | NLVKN |
| P08648 | ITA5_HUMAN | 154-158 | 5 | ESEFR | ESEFR |
| P18084 | ITB5_HUMAN | 175-179 | 5 | FLMDL | YLMDL |
| Q96AE7 | TTC17_HUMAN | 109-113 | 5 | TLDSK | TLESK |
| P48357 | LEPR_HUMAN | 420-424 | 5 | DYNYK | NYNYK |
| P48357 | LEPR_HUMAN | 925-929 | 5 | NQFNS | DQFNS |
| Q8IWB1 | IPRI_HUMAN | 558-562 | 5 | KFLPF | KFMPF |
| Q9H6X2 | ANTR1_HUMAN | 892-896 | 5 | AALQI | AALQV |
| O75976 | CBPD_HUMAN | 124-128 | 5 | TNVVI | TNVVV |
| O75976 | CBPD_HUMAN | 1070-1074 | 5 | AQEKN | AQEKD |
| O75976 | CBPD_HUMAN | 329-333 | 5 | FPNIT | YPNIT |
| Q96KA5 | CLP1L_HUMAN | 699-703 | 5 | LGAEN | LGAEN |
| O15230 | LAMA5_HUMAN | 579-583 | 5 | PQTLE | PQTLE |
| O15230 | LAMA5_HUMAN | 76-80 | 5 | TKRFD | TKRFE |
| Q3T906 | GNPTA_HUMAN | 56-60 | 5 | LPFFS | LPYFS |
| Q9NYQ6 | CELR1_HUMAN | 324-328 | 5 | ESIVR | DSIVR |
| Q9NYQ6 | CELR1_HUMAN | 581-585 | 5 | TLEIL | TLEIL |
| Q9NYQ6 | CELR1_HUMAN | 83-87 | 5 | VLPFN | VLPFD |
| Q9NYQ6 | CELR1_HUMAN | 58-62 | 5 | FFSNV | YFSNV |
| Q9NYQ6 | CELR1_HUMAN | 425-429 | 5 | LPDDF | LPEDF |
| Q9NYQ6 | CELR1_HUMAN | 427-431 | 5 | DDFTG | EDFTG |
| Q8TCT8 | SPP2A_HUMAN | 890-894 | 5 | AGAAL | AGAAL |
| Q14118 | DAG1_HUMAN | 273-277 | 5 | RTFLL | RTFLL |
| P25942 | TNR5_HUMAN | 58-62 | 5 | FFSNV | FFSNV |
| Q13308 | PTK7_HUMAN | 806-810 | 5 | LPDPS | LPEPS |
| P46977 | STT3A_HUMAN | 503-507 | 5 | VGYQP | VGFQP |
| Q13308 | PTK7_HUMAN | 338-342 | 5 | FGEVF | FGEVF |
| P11362 | FGFR1_HUMAN | 417-421 | 5 | KIADY | KIADF |
| P11362 | FGFR1_HUMAN | 188-192 | 5 | NLREF | NLREY |
| Q5JRA6 | TGO1_HUMAN | 368-372 | 5 | LYNSA | LYNSA |
| Q5JRA6 | TGO1_HUMAN | 438-442 | 5 | SNNLD | SNNLN |
| Q5JRA6 | TGO1_HUMAN | 427-431 | 5 | DDFTG | EDFTG |
| O00468 | AGRIN_HUMAN | 806-810 | 5 | LPDPS | LPDPS |
| O15118 | NPC1_HUMAN | 524-528 | 5 | VCGPK | VCGPK |
| P04626 | ERBB2_HUMAN | 145-149 | 5 | YHKNN | FHKNN |
| P04626 | ERBB2_HUMAN | 1140-1144 | 5 | PLQPE | PLQPE |
| P04626 | ERBB2_HUMAN | 639-643 | 5 | GSNVF | GSDVF |
| P04626 | ERBB2_HUMAN | 620-624 | 5 | VPVAI | IPVAI |
| Q15262 | PTPRK_HUMAN | 813-817 | 5 | SKRSF | SKRSF |
| P17301 | ITA2_HUMAN | 371-375 | 5 | SASFS | SASFS |
| P17301 | ITA2_HUMAN | 406-410 | 5 | EVRQI | DVRQI |
| P17301 | ITA2_HUMAN | 606-610 | 5 | NQVAV | NQVAI |
| P17301 | ITA2_HUMAN | 540-544 | 5 | NFNFN | NFDFN |
| P57087 | JAM2_HUMAN | 183-187 | 5 | QGNFK | QGDFK |
| Q9UQ53 | MGT4B_HUMAN | 677-681 | 5 | QTNSP | QTDSP |
| Q9UQ53 | MGT4B_HUMAN | 83-87 | 5 | VLPFN | VLPFD |
| Q9UQ53 | MGT4B_HUMAN | 872-876 | 5 | QYTSA | QYTSA |
| Q12767 | TMM94_HUMAN | 497-501 | 5 | FQPTN | FQPTD |
| Q9NX62 | IMPA3_HUMAN | 404-408 | 5 | GDEVR | GDEVR |
| P54802 | ANAG_HUMAN | 794-798 | 5 | IKDFG | IKEFG |
| P54802 | ANAG_HUMAN | 363-367 | 5 | ADYSV | ADFSV |
| Q6UXH1 | CREL2_HUMAN | 582-586 | 5 | LEILD | LEILE |
| P10586 | PTPRF_HUMAN | 209-213 | 5 | PINLV | PIDLV |
| P10586 | PTPRF_HUMAN | 921-925 | 5 | KLIAN | KLIAD |
| Q92545 | TM131_HUMAN | 603-607 | 5 | NTSNQ | NTSNH |
| Q8N8Z6 | DCBD1_HUMAN | 1131-1135 | 5 | GIVNN | GIVNN |
| Q8N8Z6 | DCBD1_HUMAN | 232-236 | 5 | GINIT | GINIT |
| Q9UHX3 | AGRE2_HUMAN | 1113-1117 | 5 | QIITT | EIITT |
| Q96RD7 | PANX1_HUMAN | 437-441 | 5 | NSNNL | NSNNL |
| Q9UIG8 | SO3A1_HUMAN | 650-654 | 5 | LIGAE | LIGAQ |
| Q8N766 | EMC1_HUMAN | 253-257 | 5 | DSSSG | ESSSG |
| Q6YHK3 | CD109_HUMAN | 282-286 | 5 | NGTIT | NGTIT |
| Q6YHK3 | CD109_HUMAN | 153-157 | 5 | MESEF | LESEF |
| Q6YHK3 | CD109_HUMAN | 401-405 | 5 | VIRGD | VIRGE |
| P00533 | EGFR_HUMAN | 890-894 | 5 | AGAAL | AGAAL |
| P00533 | EGFR_HUMAN | 1125-1129 | 5 | NCDVV | NCEVV |
| Q9BU23 | LMF2_HUMAN | 447-451 | 5 | GNYNY | GNYNF |
| P00533 | EGFR_HUMAN | 122-126 | 5 | NATNV | NATNI |
| P00533 | EGFR_HUMAN | 620-624 | 5 | VPVAI | IPVAI |
| P41440 | S19A1_HUMAN | 1072-1076 | 5 | EKNFT | DKNFT |
| Q13332 | PTPRS_HUMAN | 986-990 | 5 | KVEAE | KVEAE |
| O15439 | MRP4_HUMAN | 602-606 | 5 | TNTSN | TNTSN |
| P08473 | NEP_HUMAN | 821-825 | 5 | LLFNK | LLYNK |
| Q96PD2 | DCBD2_HUMAN | 68-72 | 5 | IHVSG | IHVSG |
| P21802 | FGFR2_HUMAN | 938-942 | 5 | LSSTA | LSSTA |
| P21802 | FGFR2_HUMAN | 417-421 | 5 | KIADY | KIADF |
| P21802 | FGFR2_HUMAN | 188-192 | 5 | NLREF | NLREY |
| Q9HCN3 | TMM8A_HUMAN | 348-352 | 5 | ASVYA | ASVYA |
| O75197 | LRP5_HUMAN | 74-78 | 5 | NGTKR | DGTKR |
| O75197 | LRP5_HUMAN | 1129-1133 | 5 | VIGIV | VIGII |
| O75197 | LRP5_HUMAN | 1138-1142 | 5 | YDPLQ | YDPLE |
| Q93050 | VPP1_HUMAN | 558-562 | 5 | KFLPF | KFLPF |
| Q9BXP2 | S12A9_HUMAN | 216-220 | 5 | LPQGF | LPQGY |
| Q12866 | MERTK_HUMAN | 361-365 | 5 | CVADY | CVADF |
| Q9BXP2 | S12A9_HUMAN | 347-351 | 5 | FASVY | FASVF |
| Q86SQ4 | AGRG6_HUMAN | 178-182 | 5 | DLEGK | NLEGK |
| O75051 | PLXA2_HUMAN | 414-418 | 5 | QTGKI | ETGKI |
| Q9C0H2 | TTYH3_HUMAN | 364-368 | 5 | DYSVL | EYSVL |
| Q9C0H2 | TTYH3_HUMAN | 481-485 | 5 | NGVEG | DGVEG |
| Q99571 | P2RX4_HUMAN | 1068-1072 | 5 | VPAQE | IPAQE |
| Q9UHW9 | S12A6_HUMAN | 987-991 | 5 | VEAEV | IEAEV |
| Q9UHW9 | S12A6_HUMAN | 988-992 | 5 | EAEVQ | EAEVE |
| Q5SZK8 | FREM2_HUMAN | 914-918 | 5 | NVLYE | DVLYE |
| Q5SZK8 | FREM2_HUMAN | 700-704 | 5 | GAENS | GAENS |
| Q5SZK8 | FREM2_HUMAN | 642-646 | 5 | VFQTR | VFETR |
| O60503 | ADCY9_HUMAN | 365-369 | 5 | YSVLY | YSVLF |
| P08069 | IGF1R_HUMAN | 80-84 | 5 | DNPVL | NNPVL |
| Q14517 | FAT1_HUMAN | 71-75 | 5 | SGTNG | SGTNG |
| Q14517 | FAT1_HUMAN | 446-450 | 5 | GGNYN | GGNYD |
| Q14517 | FAT1_HUMAN | 937-941 | 5 | SLSST | SLSST |
| Q14517 | FAT1_HUMAN | 938-942 | 5 | LSSTA | LSSTA |
| Q14517 | FAT1_HUMAN | 931-935 | 5 | IGKIQ | IGKIH |
| Q14517 | FAT1_HUMAN | 126-130 | 5 | VVIKV | VVVKV |
| Q6UVK1 | CSPG4_HUMAN | 619-623 | 5 | EVPVA | EVPVA |
| Q6UVK1 | CSPG4_HUMAN | 370-374 | 5 | NSASF | NSASY |
| Q6UVK1 | CSPG4_HUMAN | 269-273 | 5 | YLQPR | YLEPR |
| O43157 | PLXB1_HUMAN | 1141-1145 | 5 | LQPEL | LQPEL |
| Q32P28 | P3H1_HUMAN | 25-29 | 5 | PPAYT | PPAYT |
| Q92859 | NEO1_HUMAN | 805-809 | 5 | ILPDP | VLPDP |
| Q92859 | NEO1_HUMAN | 519-523 | 5 | HAPAT | HAPAT |
| Q96SJ8 | TSN18_HUMAN | 347-351 | 5 | FASVY | FASVF |
| O75882 | ATRN_HUMAN | 797-801 | 5 | FGGFN | FGGFN |
| Q9Y6A1 | POMT1_HUMAN | 55-59 | 5 | FLPFF | YLPFF |
| Q9UKA4 | AKA11_HUMAN | 458-462 | 5 | KSNLK | KSDLK |
| P48723 | HSP13_HUMAN | 1135-1139 | 5 | NTVYD | NTIYD |
| O43909 | EXTL3_HUMAN | 81-85 | 5 | NPVLP | DPVLP |
| Q969P0 | IGSF8_HUMAN | 473-477 | 5 | YQAGS | YQAGS |
| O43909 | EXTL3_HUMAN | 537-541 | 5 | KCVNF | KCINF |
| O43505 | B4GA1_HUMAN | 679-683 | 5 | NSPRR | NSPRR |
| O15440 | MRP5_HUMAN | 927-931 | 5 | FNSAI | FNSAI |
| O15440 | MRP5_HUMAN | 348-352 | 5 | ASVYA | ASIYA |
| Q9Y6M7 | S4A7_HUMAN | 618-622 | 5 | TEVPV | TEVPV |
| Q8NHS3 | MFSD8_HUMAN | 606-610 | 5 | NQVAV | DQVAV |
| O95427 | PIGN_HUMAN | 1127-1131 | 5 | DVVIG | NVVIG |
| Q9UBV2 | SE1L1_HUMAN | 110-114 | 5 | LDSKT | LDSKT |
| Q05707 | COEA1_HUMAN | 108-112 | 5 | TTLDS | TTLDS |
| Q05707 | COEA1_HUMAN | 72-76 | 5 | GTNGT | GTDGT |
| Q05707 | COEA1_HUMAN | 213-217 | 5 | VRDLP | VRNLP |
| Q05707 | COEA1_HUMAN | 1068-1072 | 5 | VPAQE | VPAQQ |
| Q05707 | COEA1_HUMAN | 113-117 | 5 | KTQSL | KTQSL |
| Q05707 | COEA1_HUMAN | 107-111 | 5 | GTTLD | GTTLD |
| Q9Y2C2 | UST_HUMAN | 175-179 | 5 | FLMDL | FLLDL |
| O43462 | MBTP2_HUMAN | 1110-1114 | 5 | YEPQI | YEPQI |
| Q13449 | LSAMP_HUMAN | 628-632 | 5 | QLTPT | HLTPT |
| Q16706 | MA2A1_HUMAN | 1137-1141 | 5 | VYDPL | VYNPL |
| Q96HE7 | ERO1A_HUMAN | 921-925 | 5 | KLIAN | KLIAN |
| Q13797 | ITA9_HUMAN | 216-220 | 5 | LPQGF | LPHGF |
| Q96HE7 | ERO1A_HUMAN | 920-924 | 5 | QKLIA | EKLIA |
| Q9Y6N7 | ROBO1_HUMAN | 418-422 | 5 | IADYN | IANYN |
| Q6YBV0 | S36A4_HUMAN | 1094-1098 | 5 | VFVSN | VFISN |
| Q6YBV0 | S36A4_HUMAN | 1067-1071 | 5 | YVPAQ | YVPAE |
| O95754 | SEM4F_HUMAN | 364-368 | 5 | DYSVL | NYSVL |
| O95754 | SEM4F_HUMAN | 619-623 | 5 | EVPVA | EVPVA |
| Q9H2H9 | S38A1_HUMAN | 105-109 | 5 | IFGTT | VFGTT |
| P27037 | AVR2A_HUMAN | 1114-1118 | 5 | IITTD | IITTE |
| P35052 | GPC1_HUMAN | 425-429 | 5 | LPDDF | LPDDY |
| O75054 | IGSF3_HUMAN | 320-324 | 5 | VQPTE | VQPTD |
| P35052 | GPC1_HUMAN | 406-410 | 5 | EVRQI | EVRQI |
| Q9Y274 | SIA10_HUMAN | 515-519 | 5 | FELLH | YELLH |
| Q14126 | DSG2_HUMAN | 109-113 | 5 | TLDSK | TLNSK |
| Q7Z5N4 | SDK1_HUMAN | 213-217 | 5 | VRDLP | VRELP |
| Q7Z5N4 | SDK1_HUMAN | 551-555 | 5 | VLTES | VLTES |
| Q7Z5N4 | SDK1_HUMAN | 1137-1141 | 5 | VYDPL | VYEPL |
| Q9UIQ6 | LCAP_HUMAN | 1097-1101 | 5 | SNGTH | SNGTQ |
| P50443 | S26A2_HUMAN | 500-504 | 5 | TNGVG | TNGVG |
| Q9Y219 | JAG2_HUMAN | 217-221 | 5 | PQGFS | PQGFS |
| P29317 | EPHA2_HUMAN | 619-623 | 5 | EVPVA | EVPVA |
| P29317 | EPHA2_HUMAN | 620-624 | 5 | VPVAI | VPVAI |
| P29317 | EPHA2_HUMAN | 338-342 | 5 | FGEVF | FGEVY |
| P55011 | S12A2_HUMAN | 935-939 | 5 | QDSLS | EDSLS |
| Q8N441 | FGRL1_HUMAN | 253-257 | 5 | DSSSG | DSSSG |
| Q14643 | ITPR1_HUMAN | 23-27 | 5 | QLPPA | ELPPA |
| Q14643 | ITPR1_HUMAN | 517-521 | 5 | LLHAP | LLQAP |
| Q8N441 | FGRL1_HUMAN | 1128-1132 | 5 | VVIGI | VVIGI |
| Q14643 | ITPR1_HUMAN | 846-850 | 5 | ARDLI | ARNLI |
| Q9H8M5 | CNNM2_HUMAN | 982-986 | 5 | SRLDK | SRMDK |
| Q86WK6 | AMGO1_HUMAN | 936-940 | 5 | DSLSS | DSLSS |
| Q14643 | ITPR1_HUMAN | 672-676 | 5 | ASYQT | ASFQT |
| Q9UIW2 | PLXA1_HUMAN | 469-473 | 5 | STEIY | STQIY |
| Q9UIW2 | PLXA1_HUMAN | 440-444 | 5 | NLDSK | NLESK |
| Q9UIW2 | PLXA1_HUMAN | 476-480 | 5 | GSTPC | GSTPC |
| Q8WTV0 | SCRB1_HUMAN | 413-417 | 5 | GQTGK | GQTGK |
| Q8WTV0 | SCRB1_HUMAN | 414-418 | 5 | QTGKI | QTGKI |
| Q9NQS3 | NECT3_HUMAN | 1144-1148 | 5 | ELDSF | ELDSY |
| P27487 | DPP4_HUMAN | 936-940 | 5 | DSLSS | DSLSS |
| Q5VUB5 | F1711_HUMAN | 170-174 | 5 | YVSQP | YVSQP |
| Q6V0I7 | FAT4_HUMAN | 72-76 | 5 | GTNGT | GTNGT |
| Q6V0I7 | FAT4_HUMAN | 253-257 | 5 | DSSSG | DSSSG |
| Q6V0I7 | FAT4_HUMAN | 549-553 | 5 | TGVLT | TGILT |
| Q6V0I7 | FAT4_HUMAN | 212-216 | 5 | LVRDL | LVRDL |
| P06213 | INSR_HUMAN | 580-584 | 5 | QTLEI | ETLEI |
| Q6V0I7 | FAT4_HUMAN | 339-343 | 5 | GEVFN | GEIFN |
| Q6V0I7 | FAT4_HUMAN | 1139-1143 | 5 | DPLQP | EPLQP |
| Q6V0I7 | FAT4_HUMAN | 273-277 | 5 | RTFLL | RTFLL |
| Q6V0I7 | FAT4_HUMAN | 494-498 | 5 | SYGFQ | SYGFE |
| Q86XX4 | FRAS1_HUMAN | 506-510 | 5 | QPYRV | QPFRV |
| Q86XX4 | FRAS1_HUMAN | 1096-1100 | 5 | VSNGT | VSDGT |
| Q86XX4 | FRAS1_HUMAN | 482-486 | 5 | GVEGF | GVDGF |
| Q86XX4 | FRAS1_HUMAN | 156-160 | 5 | EFRVY | QFRVY |
| Q86XX4 | FRAS1_HUMAN | 320-324 | 5 | VQPTE | VQPTQ |
| Q86XX4 | FRAS1_HUMAN | 473-477 | 5 | YQAGS | YQAGS |
| Q86XX4 | FRAS1_HUMAN | 1096-1100 | 5 | VSNGT | VSDGT |
| Q86XX4 | FRAS1_HUMAN | 818-822 | 5 | IEDLL | VEDLL |
| Q86XX4 | FRAS1_HUMAN | 819-823 | 5 | EDLLF | EDLLF |
| Q86XX4 | FRAS1_HUMAN | 826-830 | 5 | VTLAD | VTLAD |
| Q86XX4 | FRAS1_HUMAN | 828-832 | 5 | LADAG | LAEAG |
| Q9H6B4 | CLMP_HUMAN | 253-257 | 5 | DSSSG | ESSSG |
| Q04912 | RON_HUMAN | 576-580 | 5 | VRDPQ | VRDPQ |
| P43003 | EAA1_HUMAN | 1128-1132 | 5 | VVIGI | VVIGI |
| Q9HCM3 | K1549_HUMAN | 551-555 | 5 | VLTES | ILTES |
| Q7Z2K6 | ERMP1_HUMAN | 124-128 | 5 | TNVVI | TNVVV |
| P43007 | SATT_HUMAN | 253-257 | 5 | DSSSG | NSSSG |
| P22607 | FGFR3_HUMAN | 417-421 | 5 | KIADY | KIADF |
| P22607 | FGFR3_HUMAN | 188-192 | 5 | NLREF | NLREF |
| Q9P2B2 | FPRP_HUMAN | 401-405 | 5 | VIRGD | VIRGD |
| Q58EX2 | SDK2_HUMAN | 802-806 | 5 | FSQIL | FSEIL |
| Q58EX2 | SDK2_HUMAN | 683-687 | 5 | RARSV | RARSV |
| Q01974 | ROR2_HUMAN | 988-992 | 5 | EAEVQ | EAQVQ |
| Q58EX2 | SDK2_HUMAN | 701-705 | 5 | AENSV | AENSV |
| Q9BZC7 | ABCA2_HUMAN | 489-493 | 5 | YFPLQ | YFPLQ |
| Q9BZC7 | ABCA2_HUMAN | 802-806 | 5 | FSQIL | FSQIL |
| Q9BZC7 | ABCA2_HUMAN | 642-646 | 5 | VFQTR | IFQTR |
| Q9BZC7 | ABCA2_HUMAN | 916-920 | 5 | LYENQ | LYENQ |
| Q9BZC7 | ABCA2_HUMAN | 23-27 | 5 | QLPPA | ELPPA |
| P78357 | CNTP1_HUMAN | 233-237 | 5 | INITR | INITR |
| P08183 | MDR1_HUMAN | 177-181 | 5 | MDLEG | MDLEG |
| P08183 | MDR1_HUMAN | 106-110 | 5 | FGTTL | YGTTL |
| O43490 | PROM1_HUMAN | 186-190 | 5 | FKNLR | FKDLR |
| P54289 | CA2D1_HUMAN | 843-847 | 5 | DIAAR | EIAAR |
| P54289 | CA2D1_HUMAN | 844-848 | 5 | IAARD | IAARD |
| Q9NT99 | LRC4B_HUMAN | 1076-1080 | 5 | TTAPA | TTAPA |
| Q8TCT7 | SPP2B_HUMAN | 1128-1132 | 5 | VVIGI | VVIGI |
| Q8IWK6 | AGRA3_HUMAN | 474-478 | 5 | QAGST | QAGST |
| O75096 | LRP4_HUMAN | 171-175 | 5 | VSQPF | VSHPF |
| O75096 | LRP4_HUMAN | 638-642 | 5 | TGSNV | TGSNV |
| P69849 | NOMO3_HUMAN | 325-329 | 5 | SIVRF | SIIRF |
| P69849 | NOMO3_HUMAN | 644-648 | 5 | QTRAG | ETRAG |
| Q02487 | DSC2_HUMAN | 1130-1134 | 5 | IGIVN | IGVVN |
| P52569 | CTR2_HUMAN | 576-580 | 5 | VRDPQ | VRNPQ |
| Q06418 | TYRO3_HUMAN | 361-365 | 5 | CVADY | CVADF |
| Q6NSJ0 | MYORG_HUMAN | 56-60 | 5 | LPFFS | LPFFS |
| Q6NSJ0 | MYORG_HUMAN | 806-810 | 5 | LPDPS | LPDPS |
| P48029 | SC6A8_HUMAN | 482-486 | 5 | GVEGF | GVEGF |
| O60279 | SUSD5_HUMAN | 581-585 | 5 | TLEIL | TLEIL |
| O60279 | SUSD5_HUMAN | 513-517 | 5 | LSFEL | LSYEL |
| P29323 | EPHB2_HUMAN | 281-285 | 5 | ENGTI | ENGTV |
| Q07075 | AMPE_HUMAN | 356-360 | 5 | KRISN | KRISN |
| Q07075 | AMPE_HUMAN | 939-943 | 5 | SSTAS | SSTAS |
| O75121 | MFA3L_HUMAN | 24-28 | 5 | LPPAY | LPPAY |
| O75121 | MFA3L_HUMAN | 1133-1137 | 5 | VNNTV | VNNTV |
| Q92823 | NRCAM_HUMAN | 58-62 | 5 | FFSNV | YFSNV |
| Q92823 | NRCAM_HUMAN | 108-112 | 5 | TTLDS | TTLDS |
| O75121 | MFA3L_HUMAN | 141-145 | 5 | LGVYY | MGVYY |
| Q15223 | NECT1_HUMAN | 981-985 | 5 | LSRLD | LSRLE |
| Q99835 | SMO_HUMAN | 987-991 | 5 | VEAEV | VEAEI |
| Q9HC56 | PCDH9_HUMAN | 425-429 | 5 | LPDDF | LPNDF |
| Q9HC56 | PCDH9_HUMAN | 549-553 | 5 | TGVLT | TGVLT |
| O76082 | S22A5_HUMAN | 939-943 | 5 | SSTAS | SSTAS |
| Q9UN71 | PCDGG_HUMAN | 938-942 | 5 | LSSTA | LSSTA |
| Q9UN71 | PCDGG_HUMAN | 285-289 | 5 | ITDAV | VTDAV |
| Q9ULK0 | GRID1_HUMAN | 471-475 | 5 | EIYQA | EIYQA |
| P20023 | CR2_HUMAN | 376-380 | 5 | TFKCY | TFKCY |
| Q9P0K1 | ADA22_HUMAN | 176-180 | 5 | LMDLE | LMELE |
| P18825 | ADA2C_HUMAN | 682-686 | 5 | RRARS | RRARS |
| P20023 | CR2_HUMAN | 659-663 | 5 | SYECD | SYECD |
| P98164 | LRP2_HUMAN | 615-619 | 5 | VNCTE | INCTE |
| P98164 | LRP2_HUMAN | 616-620 | 5 | NCTEV | NCTEI |
| P98164 | LRP2_HUMAN | 614-618 | 5 | DVNCT | DVDCT |
| P98164 | LRP2_HUMAN | 480-484 | 5 | CNGVE | CNGVD |
| P98164 | LRP2_HUMAN | 819-823 | 5 | EDLLF | EDLLY |
| P98164 | LRP2_HUMAN | 480-484 | 5 | CNGVE | CNGVD |
| P98164 | LRP2_HUMAN | 480-484 | 5 | CNGVE | CNGVD |
| P98164 | LRP2_HUMAN | 154-158 | 5 | ESEFR | ESEFR |
| Q9HCU4 | CELR2_HUMAN | 83-87 | 5 | VLPFN | VLPFD |
| Q96T83 | SL9A7_HUMAN | 691-695 | 5 | SIIAY | SIVAY |
| Q8IZP9 | AGRG2_HUMAN | 69-73 | 5 | HVSGT | HVSGT |
| Q75V66 | ANO5_HUMAN | 206-210 | 5 | KHTPI | KHTPI |
| Q8TDW7 | FAT3_HUMAN | 937-941 | 5 | SLSST | SLSST |
| Q8TDW7 | FAT3_HUMAN | 938-942 | 5 | LSSTA | LSSTA |
| Q6N022 | TEN4_HUMAN | 107-111 | 5 | GTTLD | GTTLD |
| Q6N022 | TEN4_HUMAN | 106-110 | 5 | FGTTL | YGTTL |
| Q8TDW7 | FAT3_HUMAN | 938-942 | 5 | LSSTA | MSSTA |
| Q8TDW7 | FAT3_HUMAN | 445-449 | 5 | VGGNY | VGGNF |
| Q6N022 | TEN4_HUMAN | 532-536 | 5 | NLVKN | DLVKN |
| Q8TDW7 | FAT3_HUMAN | 984-988 | 5 | LDKVE | LDKVD |
| Q6N022 | TEN4_HUMAN | 454-458 | 5 | RLFRK | RLFRK |
| Q6N022 | TEN4_HUMAN | 622-626 | 5 | VAIHA | VAIHA |
| Q8TDW7 | FAT3_HUMAN | 931-935 | 5 | IGKIQ | IGKIH |
| Q8TDW7 | FAT3_HUMAN | 253-257 | 5 | DSSSG | DSSSG |
| Q8TDW7 | FAT3_HUMAN | 253-257 | 5 | DSSSG | DSSSG |
| Q8TDW7 | FAT3_HUMAN | 425-429 | 5 | LPDDF | LPEDF |
| Q6N022 | TEN4_HUMAN | 1113-1117 | 5 | QIITT | QIITT |
| Q15818 | NPTX1_HUMAN | 249-253 | 5 | LTPGD | LTPGE |
| Q8NFQ8 | TOIP2_HUMAN | 494-498 | 5 | SYGFQ | SYGFE |
| Q9P1W3 | CSC1_HUMAN | 385-389 | 5 | TKLND | TKLNE |
| Q5VU97 | CAHD1_HUMAN | 1130-1134 | 5 | IGIVN | VGIVN |
| P14672 | GLUT4_HUMAN | 1128-1132 | 5 | VVIGI | IVIGI |
| Q9P1W3 | CSC1_HUMAN | 384-388 | 5 | PTKLN | PTKLN |

1. **Supplementary Figures**

**(A)**
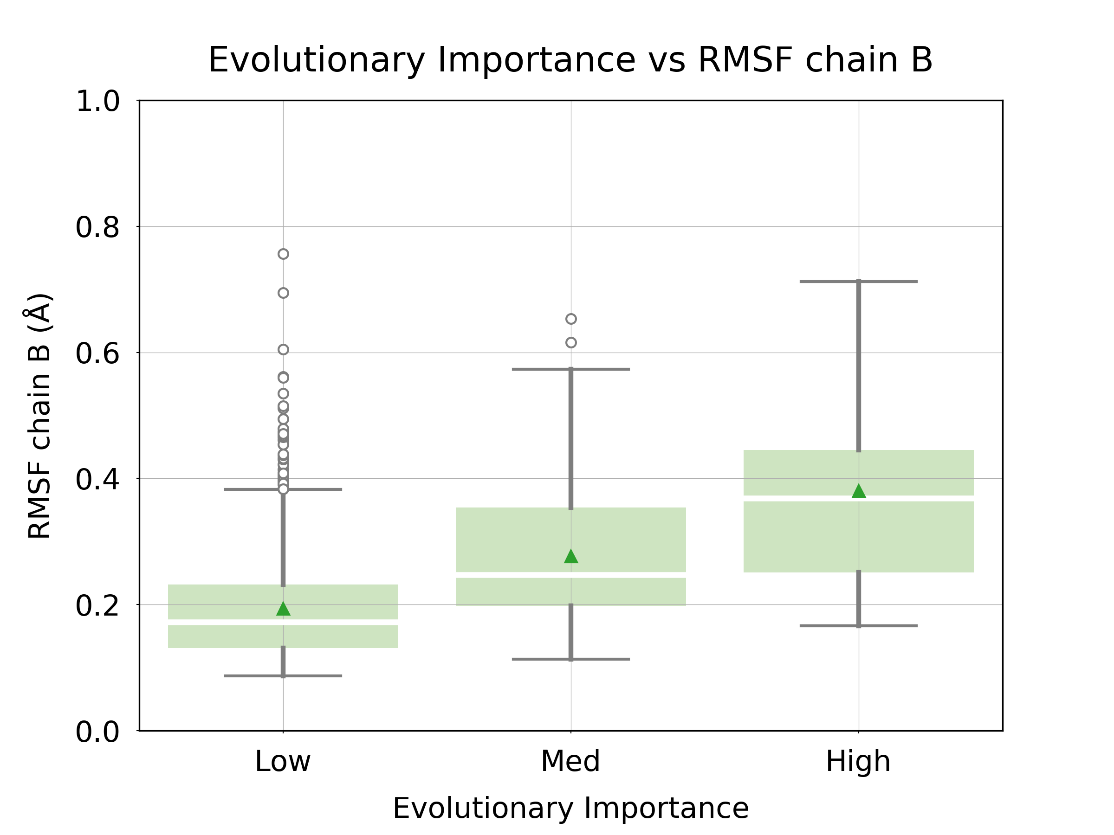


**(B)**
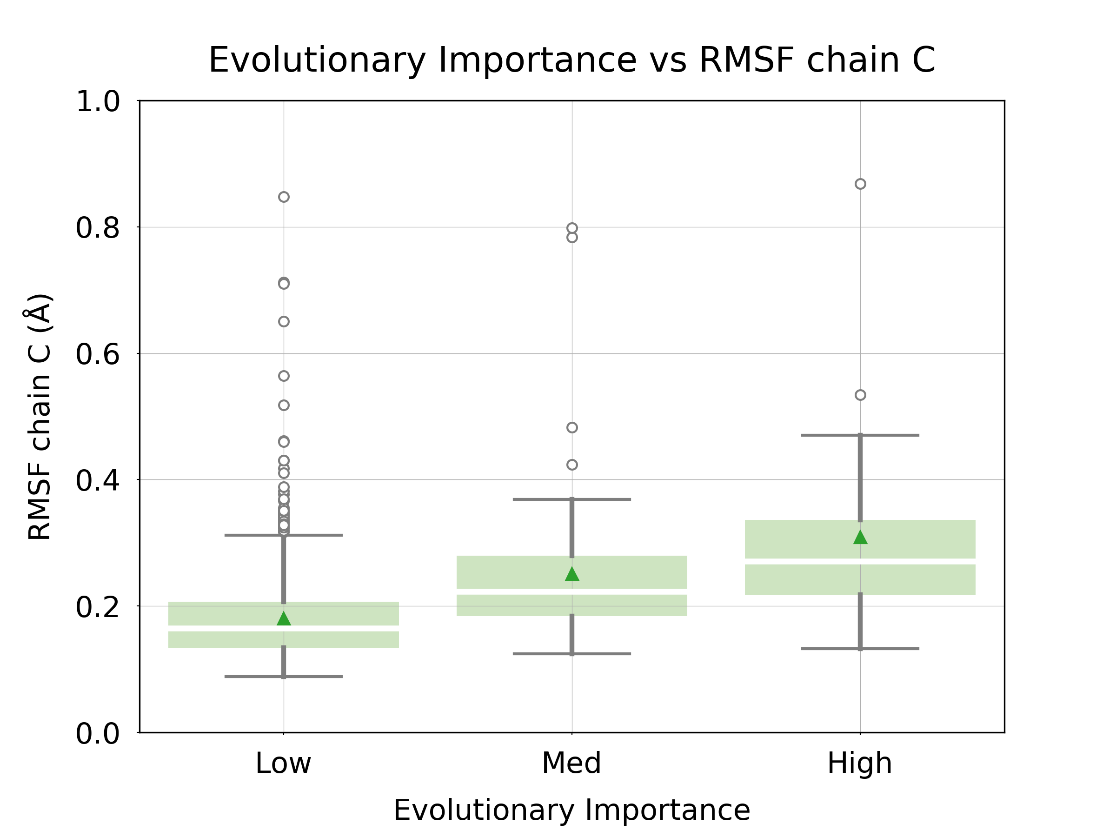


**Supplementary Figure 1. Box-plot of RMSF as a function of selection pressure indicating selection pressure is higher in flexible regions of two other spike protein chains. (A)** Chain A; **(B)** Chain B.
